# Supplementary material for: Iron dysregulation and inflammatory stress erythropoiesis associates with long-term outcome of COVID-19
Source: Nat Immunol. 2024 Mar 1;25(3):471–82. doi: 10.1038/s41590-024-01754-8 (PMC10907301; doi:10.1038/s41590-024-01754-8)
Supplement: Supplementary file 1 — Supplementary Table 1, Supplementary Figs. 1–10, Supplementary Materials, Supplementary Methods and Supplementary Note. [file 41590_2024_1754_MOESM1_ESM.pdf]

nature immunology

Article

https://doi.org/10.1038/s41590-024-01754-8

Iron dysregulation and inflammatory stress erythropoiesis associates with long-term outcome of COVID-19

In the format provided by the authors and unedited

Supplementary Table 1: Clinical and demographic features of study groups, stratified by severity A-E, and controls

	A	B	C	D	E	Hosp (late)	HC (clinical)	HC (RNASeq)
**n**	18	40	46	37	60	13	45	60
**Sex (% male)**	22.2%	22.5%	54.3%	64.9%	75.0%	64.3%	55.5%	56.7%
**Age (years, mean (SD))**	32.9 (12.7)	36.0 (11.8)	58.0 (16.9)	64.4 (15.1)	57.0 (14.9)	56.3 (11.4)	42.2 (15.2)	48.4 (19.5)
**Days from COVID-19 symptoms to enrollment (days, mean (SD))**	NA	6.5 (2.9)	11.4 (6.7)	10.6 (8.1)	24.6 (14.3)	149.2 (11.5)	NA	NA
**COVID-19 chest radiology**	NA	NA	50.0%	89.2%	100%	78.6%	NA	NA
**Non-COVID19 admissions**	NA	NA	30.2%	8.1%	6.7%	7.1%	NA	NA
**Haemoglobin (g/L, mean (SD))**	137.0 (8.1)	143.0 (15.6)	124.8 (16.0)	121.6 (18.0)	95.2 (16.8)	135.5 (17.4)	140.4 (12.6)	NA
**Serum creatinine (μmol/L, mean (SD))**	NA	NA	82.9 (40.1)	117.5 (154.7)	103.5 (129.3)	107.1 (124.7)	NA	NA
**Serum albumin (g/L, mean (SD))**	NA	NA	32.4 (7.1)	28.0 (6.3)	24.4 (7.2)	40.6 (2.5)	NA	NA
**LOS (days, median (IQR))**	NA	NA	4 (1.25-10)	10 (6-16)	44 (33.7-63.2)	20 (6.7-27)	NA	NA
**Admitted to ITU**	NA	NA	0%	13.5%	90.0%	64.3%	NA	NA
**Deceased in hospital**	NA	NA	2.2%	0.0%	30.0%	0.0%	NA	NA
**Hypertension**	NA	NA	47.8%	43.2%	48.3%	57.1%	NA	NA
**CAD**	NA	NA	8.7%	24.3%	16.7%	21.4%	NA	NA
**Other heart condition**	NA	NA	10.9%	18.9%	13.3%	7.1%	NA	NA
**Diabetes mellitus**	NA	NA	26.1%	29.7%	43.3%	21.4%	NA	NA
**CKD**	NA	NA	8.7%	16.2%	8.3%	7.1%	NA	NA
**PVD**	NA	NA	6.5%	8.1%	8.3%	0.0%	NA	NA
**CVA/TIA**	NA	NA	10.9%	2.7%	6.7%	0.0%	NA	NA
**COPD**	NA	NA	6.5%	18.9%	5.0%	0.0%	NA	NA
**Asthma**	NA	NA	21.7%	10.8%	10.0%	14.3%	NA	NA
**Other lung disease**	NA	NA	10.9%	16.2%	10.0%	0.0%	NA	NA
**Cancer**	NA	NA	4.4%	5.4%	1.7%	0.0%	NA	NA
**Haematological cancer**	NA	NA	2.2%	5.4%	0.0%	0.0%	NA	NA
**Corticosteroids**	NA	NA	19.6%	10.8%	10.0%	0.0%	NA	NA
**Immunosuppressive treatment**	NA	NA	17.4%	16.2%	5.0%	14.3%	NA	NA

This table is an update of Bergamaschi et al. 2021 (Immunity) Supplementary Table S1.
Measures from patient groups A-E are from the day of study enrolment during acute COVID-19; 13 prospectively recruited previously-hospitalized patients are grouped as Hosp (late), with measures taken from the day of study enrolment. HC (clinical) are those healthy controls included in statistical comparisons of all clinical and cellular parameters (serum cytokine, iron and reticulocyte measures, and immunophenotyping). A subset of these, and 28 additional historically collected control samples, were used for RNASeq analyses; HC (RNASeq).
SD: standard deviation, *IQR:* interquartile range
COVID-19 chest radiology: chest X-ray/ CT scan showed changes compatible with COVID-19, as opposed to normal findings or lung changes diagnostic of other conditions
Non-COVID19 admissions: cases where COVID-19 was diagnosed during the hospital stay in patients initially admitted to hospital for reasons unrelated to COVID-19
Haemoglobin, serum albumin and serum creatinine: results from routine lab tests on the day of study enrollment, or closest result up to 2 days before. The included test results are available for at least for 75% of each severity group.
LOS: length of hospital stay (days from hospital admission to discharge, transfer or death in hospital)
Hypertension: history of hypertension, defined as blood pressure ≥140/80 on multiple occasions, or on treatment with any medication explicitly employed to reduce blood pressure
CAD: history of coronary artery disease, defined as myocardial infarction, angina, coronary artery stenting or coronary artery bypass grafting
Other heart condition: history of any other chronic cardiac disease (not CAD/hypertension), e.g. heart failure, congenital heart disease, cardiomyopathy, rheumatic heart disease
CKD: history of chronic kidney disease, defined as any of estimated glomerular filtration rate < 60 mL/min/1.73m^2, dialysis or kidney transplant
PVD: history of peripheral vascular disease, defined as intermittent claudication or past bypass for chronic arterial insufficiency, history of gangrene or acute arterial insufficiency, or thoracic/abdominal aneurysm (≥6 cm)
CVA/TIA: history of a cerebrovascular accident or transient ischemic attacks
COPD: history of chronic obstructive pulmonary disease
Other lung disease: history of other chronic pulmonary disease (non asthma/COPD), e.g. cystic fibrosis, bronchiectasis, interstitial lung disease
Cancer: current solid organ malignancy (active or in the last 5 years), except non-melanoma skin cancers
Corticosteroids: history of treatment with systemic corticosteroids in the 14 days prior to hospital admission/presentation
Immunosuppressive treatment: history of treatment with immunosuppressants (excluding corticosteroids) in the 14 days prior to hospital admission/presentation, or chemotherapy/biologic drugs in the previous 6 months

Supplementary Figure 1

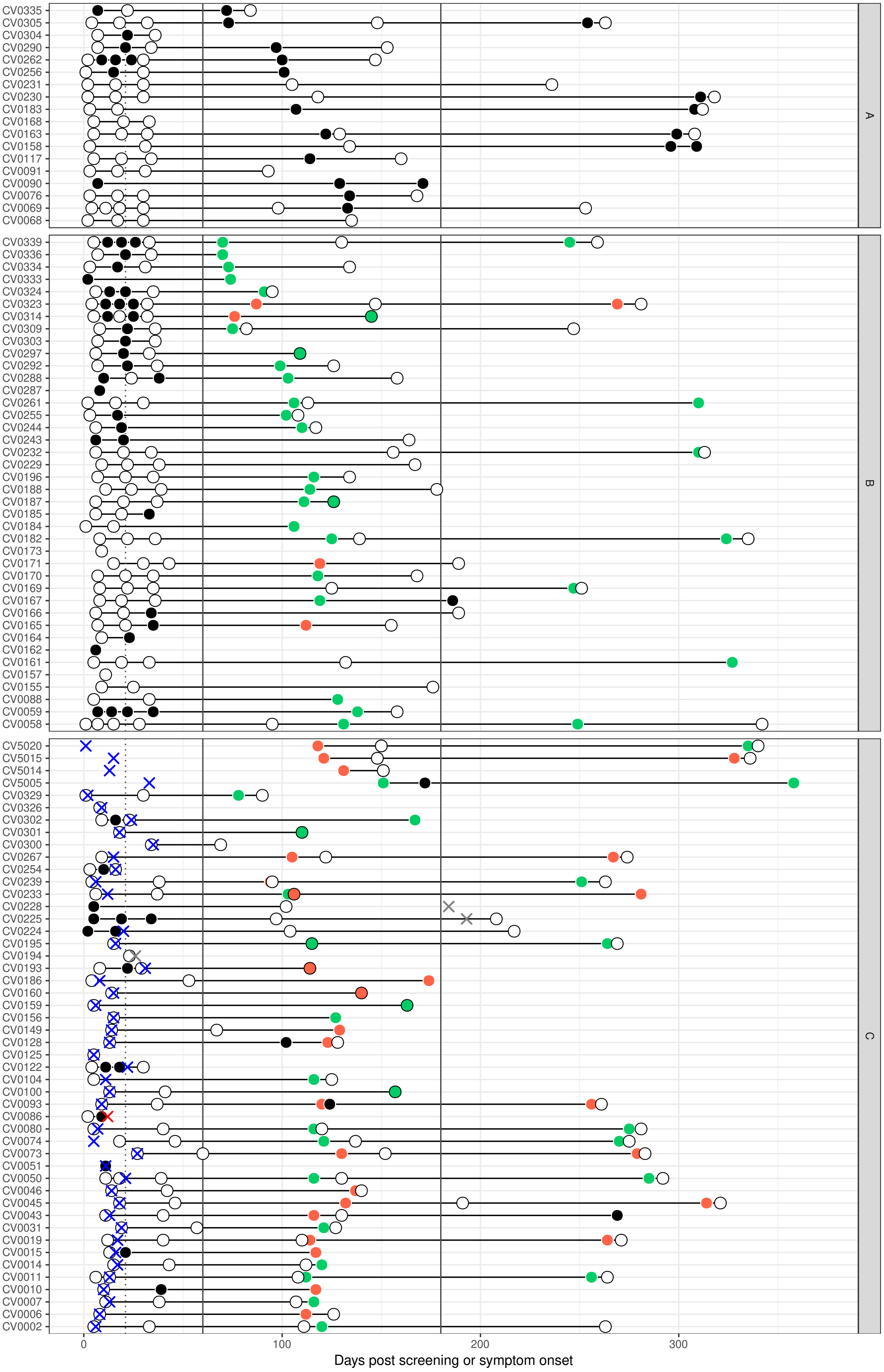

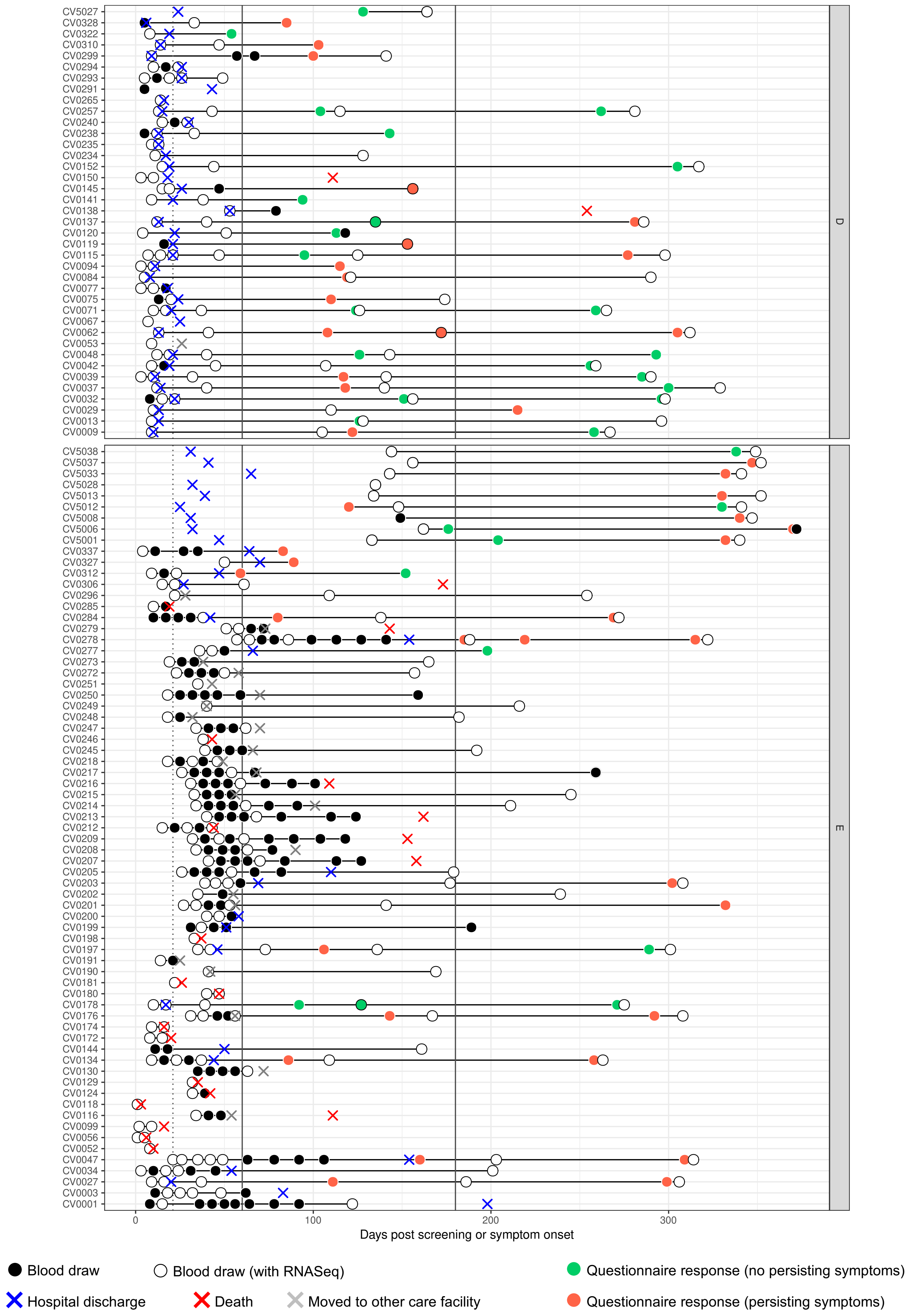
CV5027
CV0328
CV0322
CV0310
CV0299
CV0294
CV0293
CV0291
CV0265
CV0257
CV0240
CV0238
CV0235
CV0234
CV0152
CV0150
CV0145
CV0141
CV0138
CV0137
CV0120
CV0119
CV0115
CV0094
CV0084
CV0077
CV0075
CV0071
CV0067
CV0062
CV0053
CV0048
CV0042
CV0039
CV0037
CV0032
CV0029
CV0013
CV0009
D
CV5038
CV5037
CV5033
CV5028
CV5013
CV5012
CV5008
CV5006
CV5001
CV0337
CV0327
CV0312
CV0306
CV0296
CV0285
CV0284
CV0279
CV0278
CV0277
CV0273
CV0272
CV0251
CV0250
CV0249
CV0248
CV0247
CV0246
CV0245
CV0218
CV0217
CV0216
CV0215
CV0214
CV0213
CV0212
CV0209
CV0208
CV0207
CV0205
CV0203
CV0202
CV0201
CV0200
CV0199
CV0198
CV0197
CV0191
CV0190
CV0181
CV0180
CV0178
CV0176
CV0174
CV0172
CV0144
CV0134
CV0130
CV0129
CV0124
CV0118
CV0116
CV0099
CV0056
CV0052
CV0047
CV0034
CV0027
CV0003
CV0001
E
0
100
200
300
Days post screening or symptom onset
Blood draw
Blood draw (with RNASeq)
Questionnaire response (no persisting symptoms)
Hospital discharge
Death
Moved to other care facility
Questionnaire response (persisting symptoms)

Supplementary Figure 1: COVID-19 patient timelines. Timelines of sample and questionnaire collection for recruited participants across 5 COVID-19 severity groups. Black and white points represent timepoints of blood draw, without or with whole blood RNA sequencing respectively. Red and green points represent timepoint of follow-up questionnaire response, coloured according to symptom group (red = persisting symptoms, green = no persisting symptoms). Q1 questionnaires were completed between 60 and 180 DPSO (as indicated by vertical black lines), and Q2 questionnaires beyond 180 DPSO. In instances of multiple responses, the latest was used. Crosses indicate hospital discharge (blue), relocation to other care facility (grey) or death (red).

Supplementary Figure 2

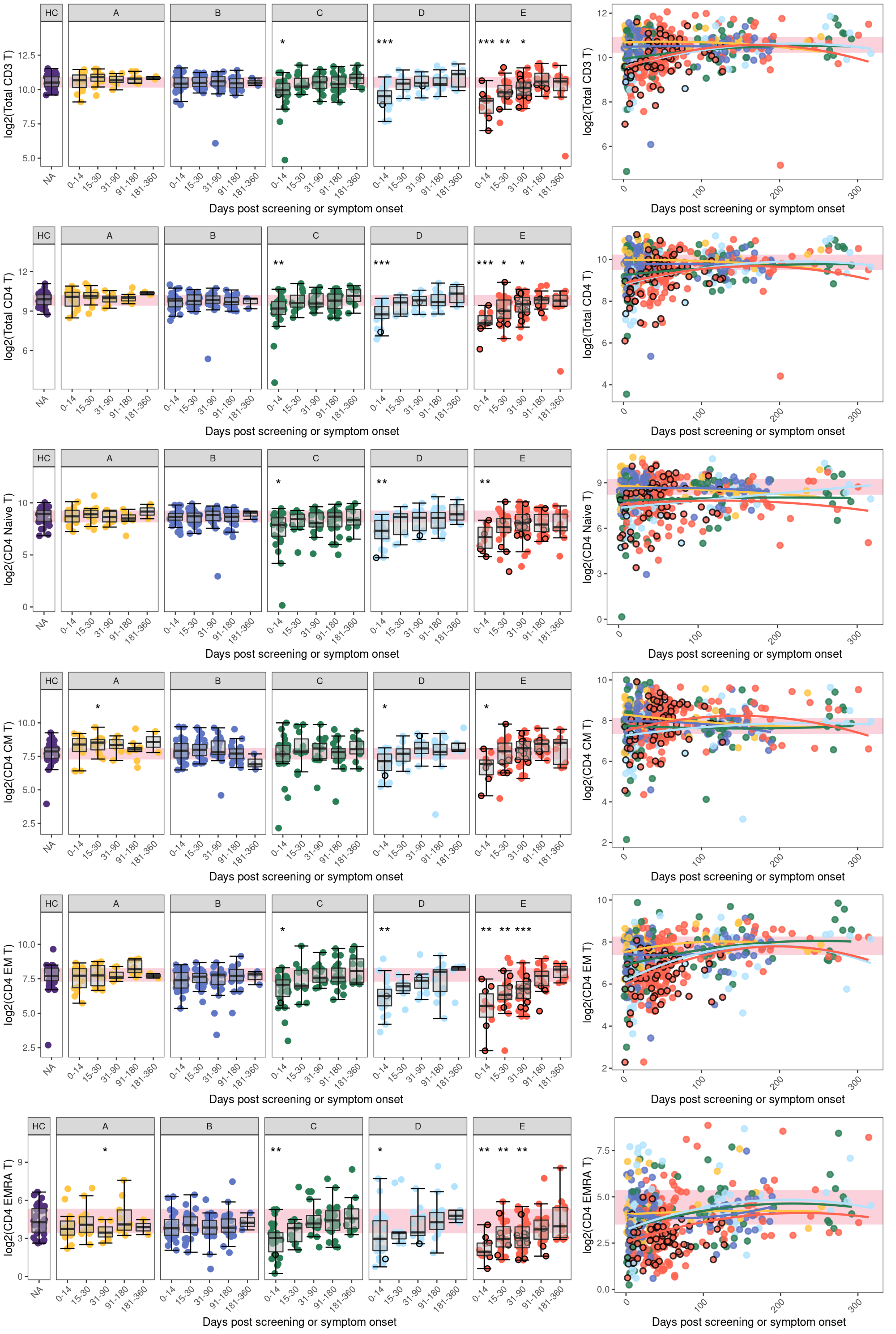

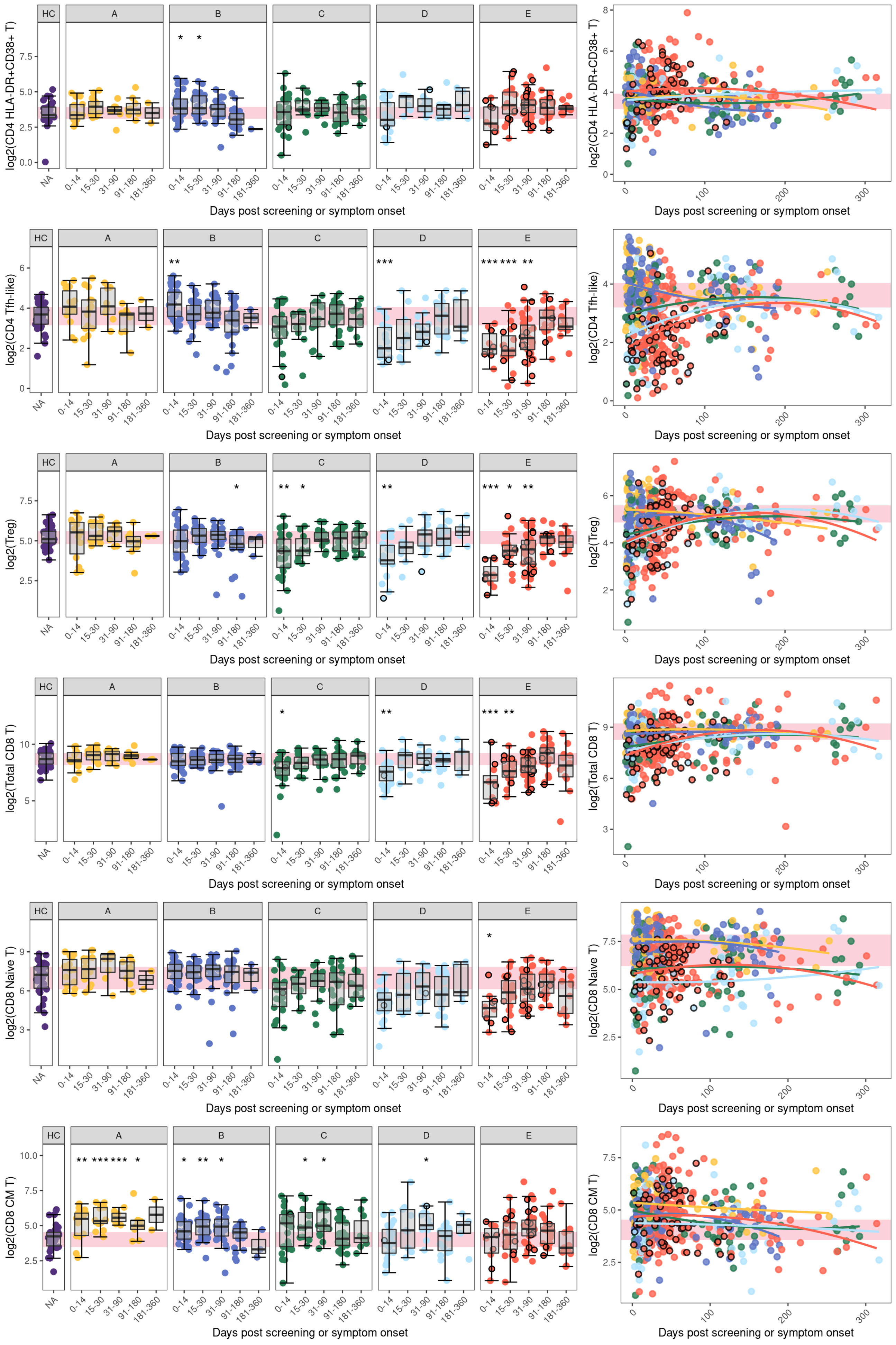

HC
A
B
C
D
E
NA
0-14
15-30
31-90
91-180
181-360
Days post screening or symptom onset
log2(CD4 HLA-DR+CD38+ T)
log2(CD4 Tfh-like)
log2(Treg)
log2(Total CD8 T)
log2(CD8 Naive T)
log2(CD8 CM T)

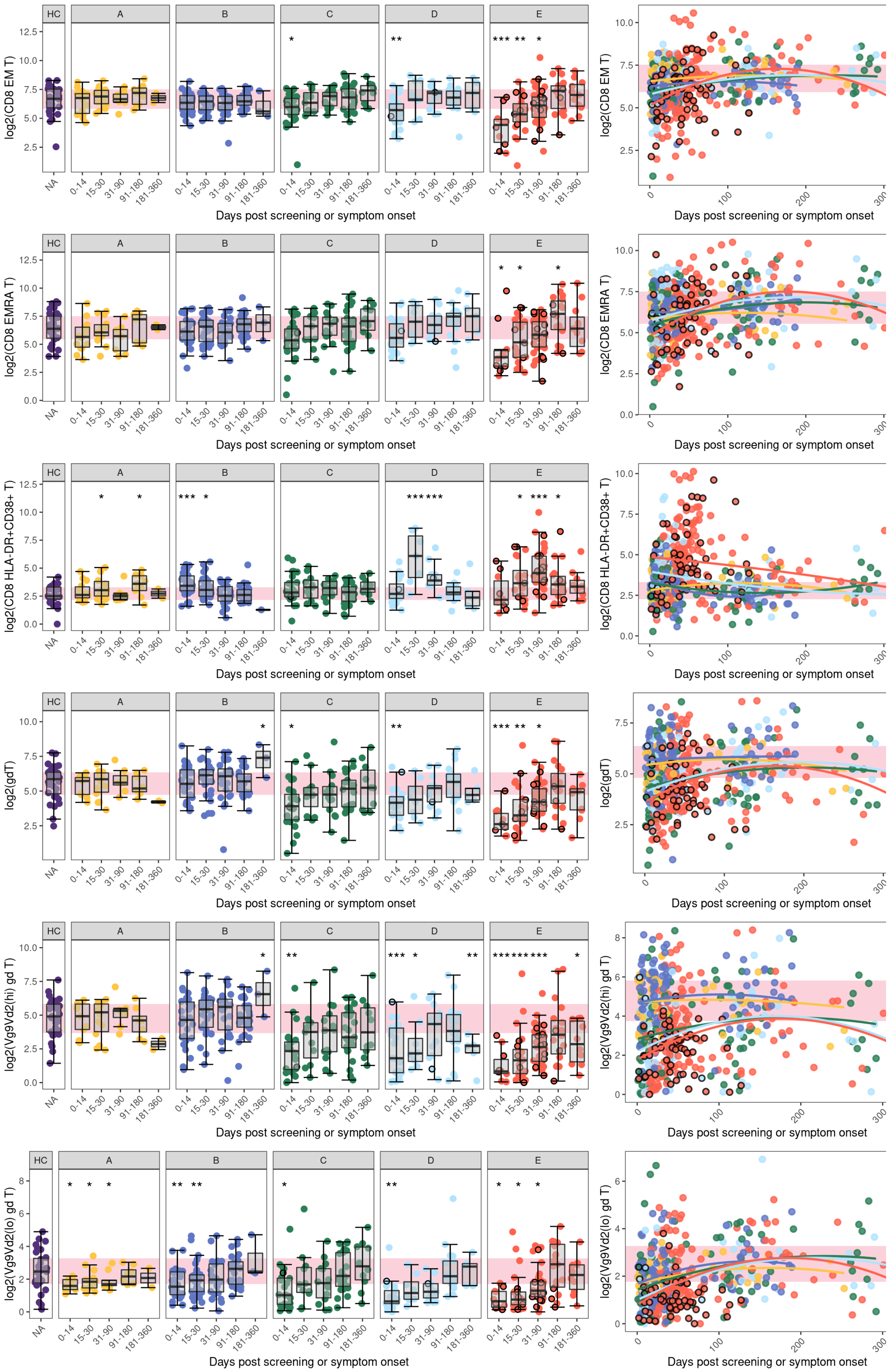

HC
A
B
C
D
E
NA
0-14
15-30
31-90
91-180
181-360
log2(CD8 EM T)
log2(CD8 EMRA T)
log2(CD8 HLA-DR+CD38+ T)
log2(gdT)
log2(Vg9Vd2(hi) gd T)
log2(Vg9Vd2(lo) gd T)
Days post screening or symptom onset

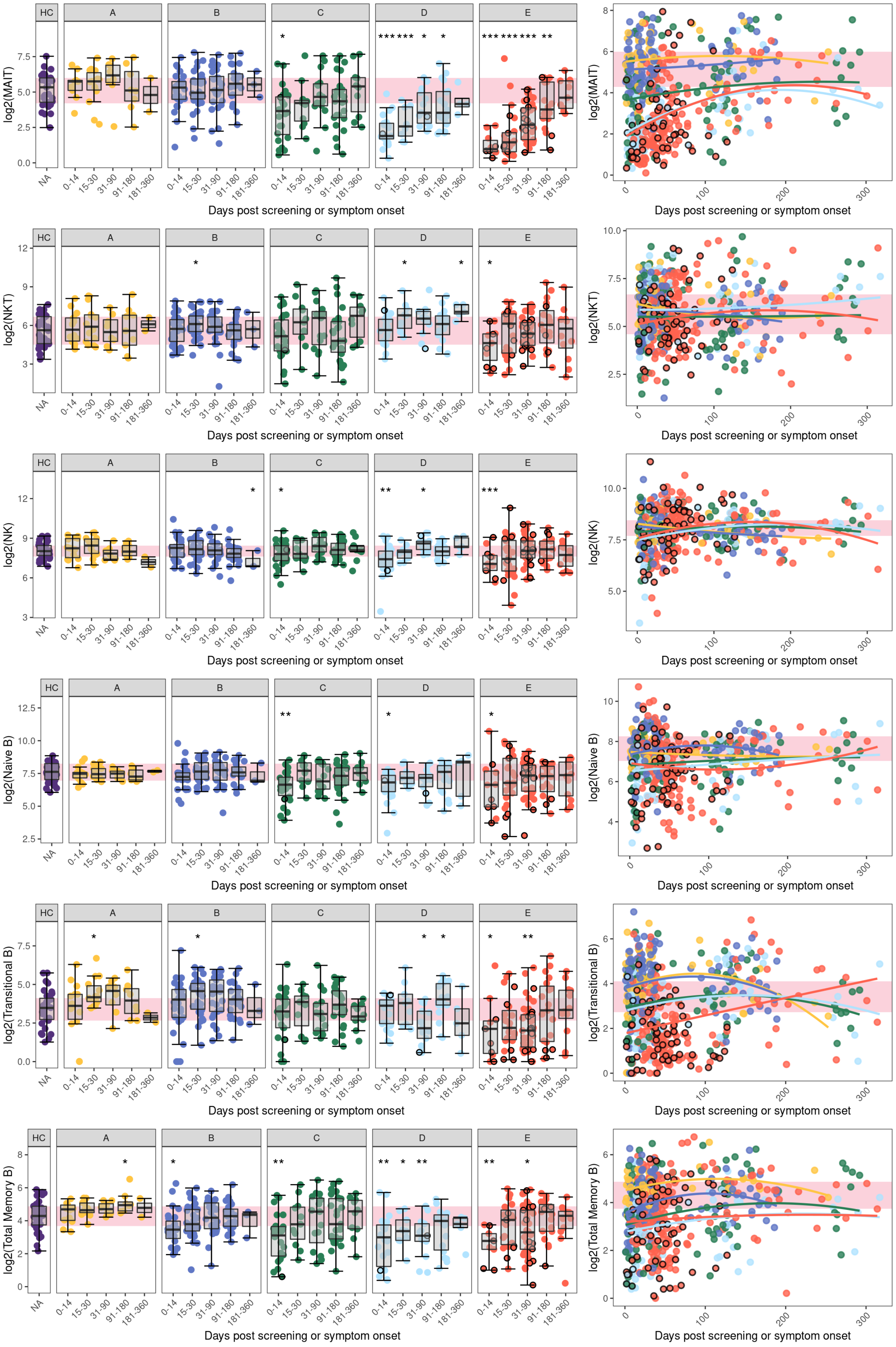
HC
A
B
C
D
E
NA
0-14
15-30
31-90
91-180
181-360
log2(MAIT)
log2(NKT)
log2(NK)
log2(Naive B)
log2(Transitional B)
log2(Total Memory B)
Days post screening or symptom onset

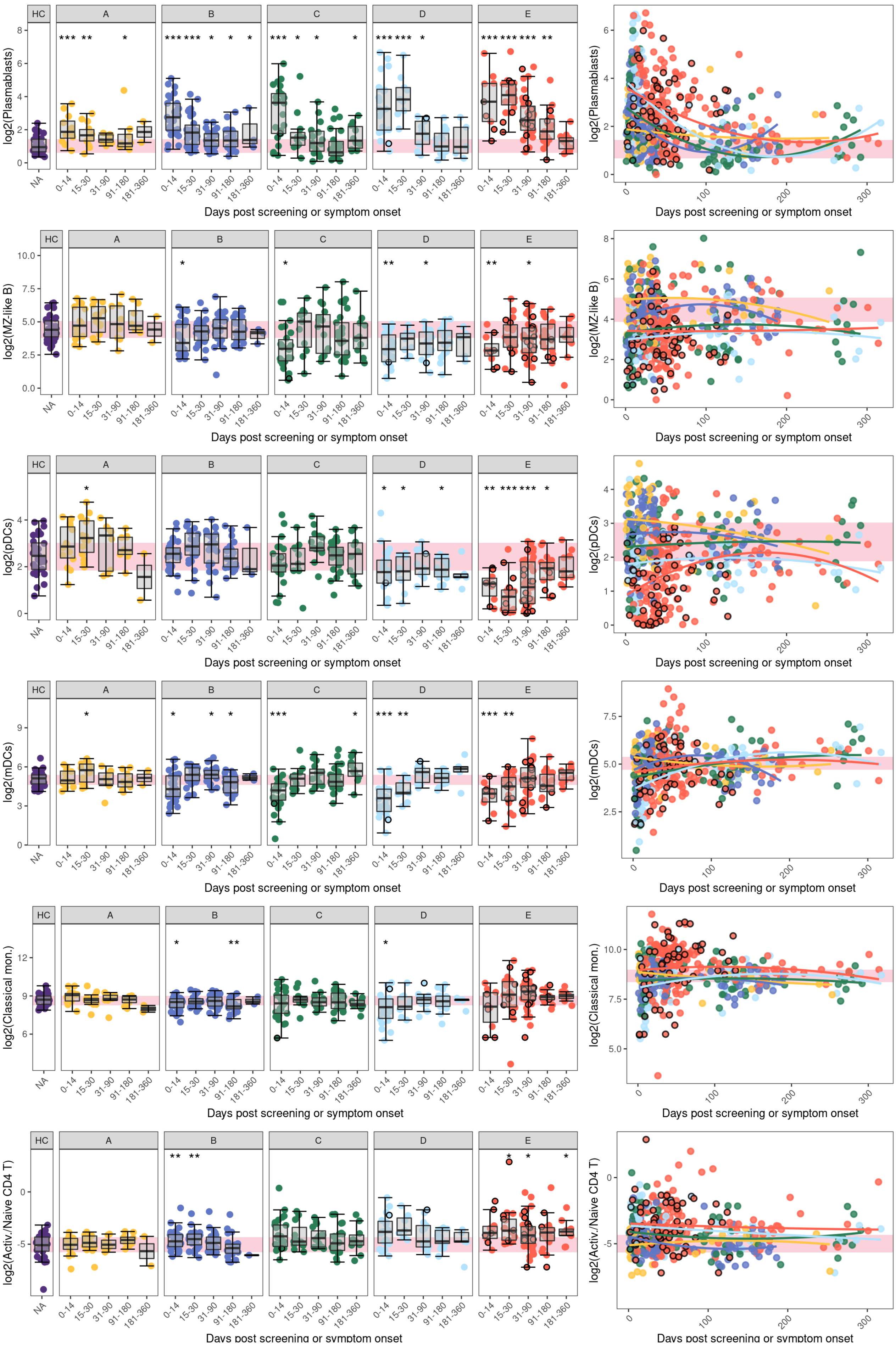
HC
A
B
C
D
E
NA
0-14
15-30
31-90
91-180
181-360
log2(Plasmablasts)
log2(MZ-like B)
log2(pDCs)
log2(mDCs)
log2(Classical mon.)
log2(Activ./Naive CD4 T)
Days post screening or symptom onset

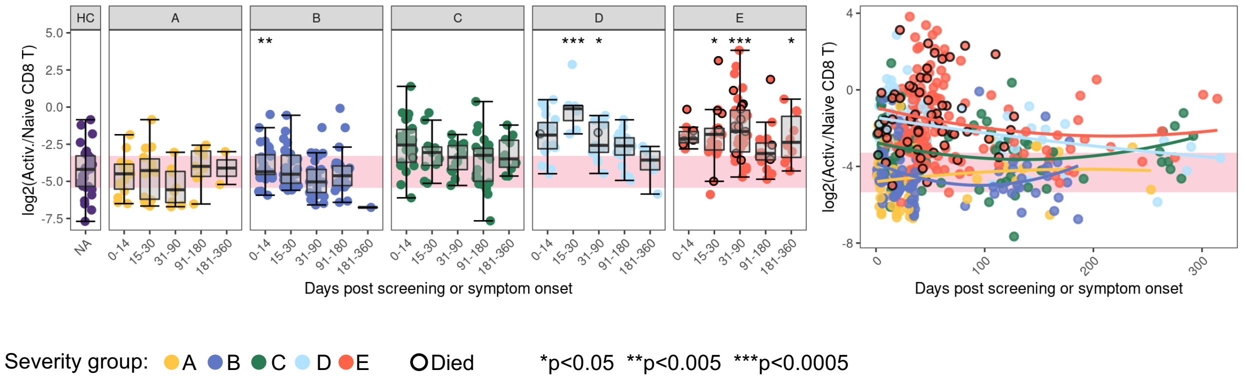

Supplementary Figure 2: Boxplots and longitudinal plots showing changes in absolute cell counts over time for each COVID-19 severity group. Cell populations and ratios summarized in Figure 1D are shown. Pink band indicates the interquartile range of the HCs. P-values are calculated by linear model of $\log_2$(count) ~ severity group + age + sex (*p<0.05, **p<0.005, ***p<0.0005). Patients who passed away over the study period are rimmed in black. Boxplots show minimum, 25th percentile, median, 75th percentile and maximum, and outliers beyond 1.5 times the interquartile range.

Supplementary Figure 3

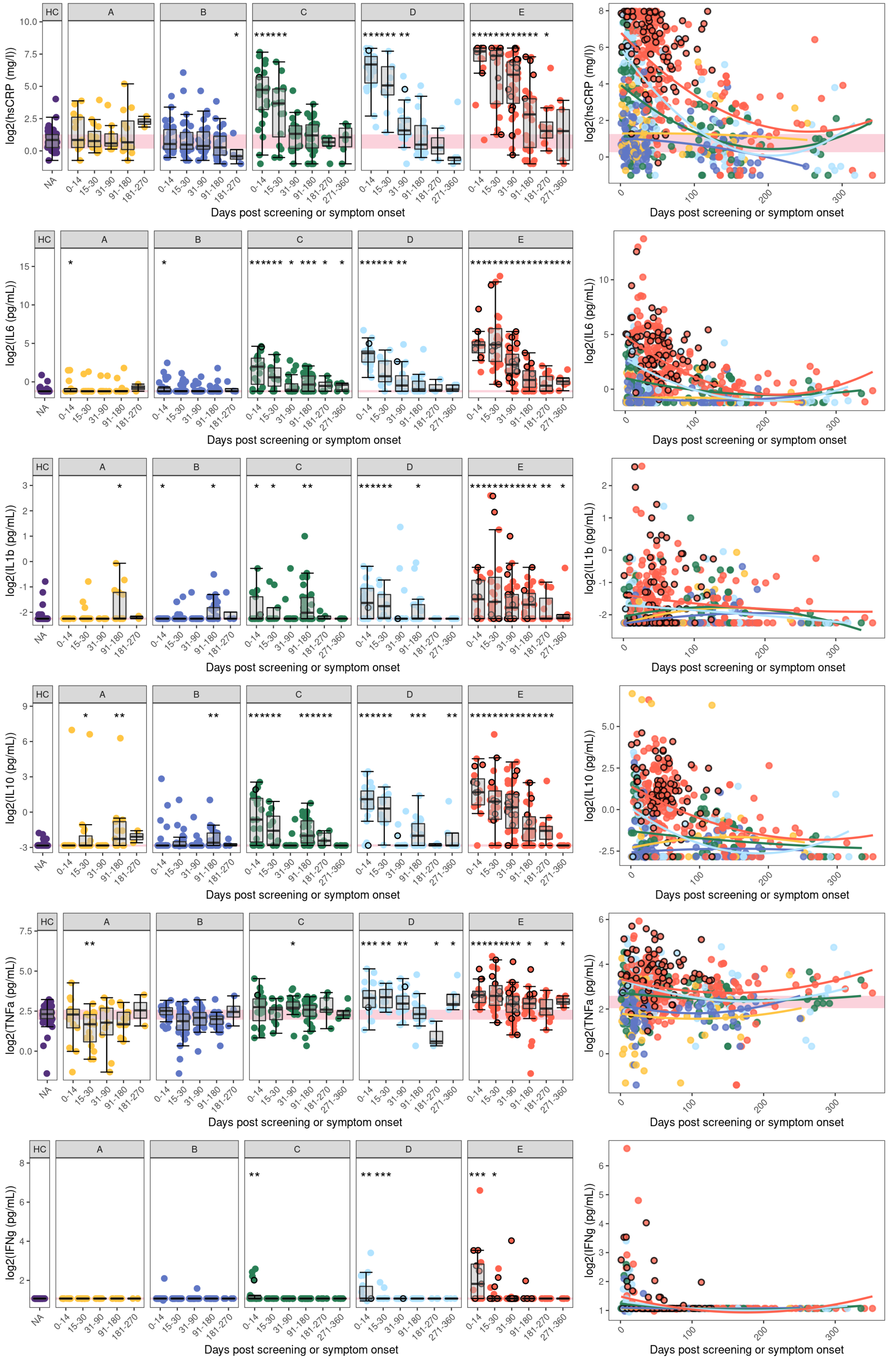

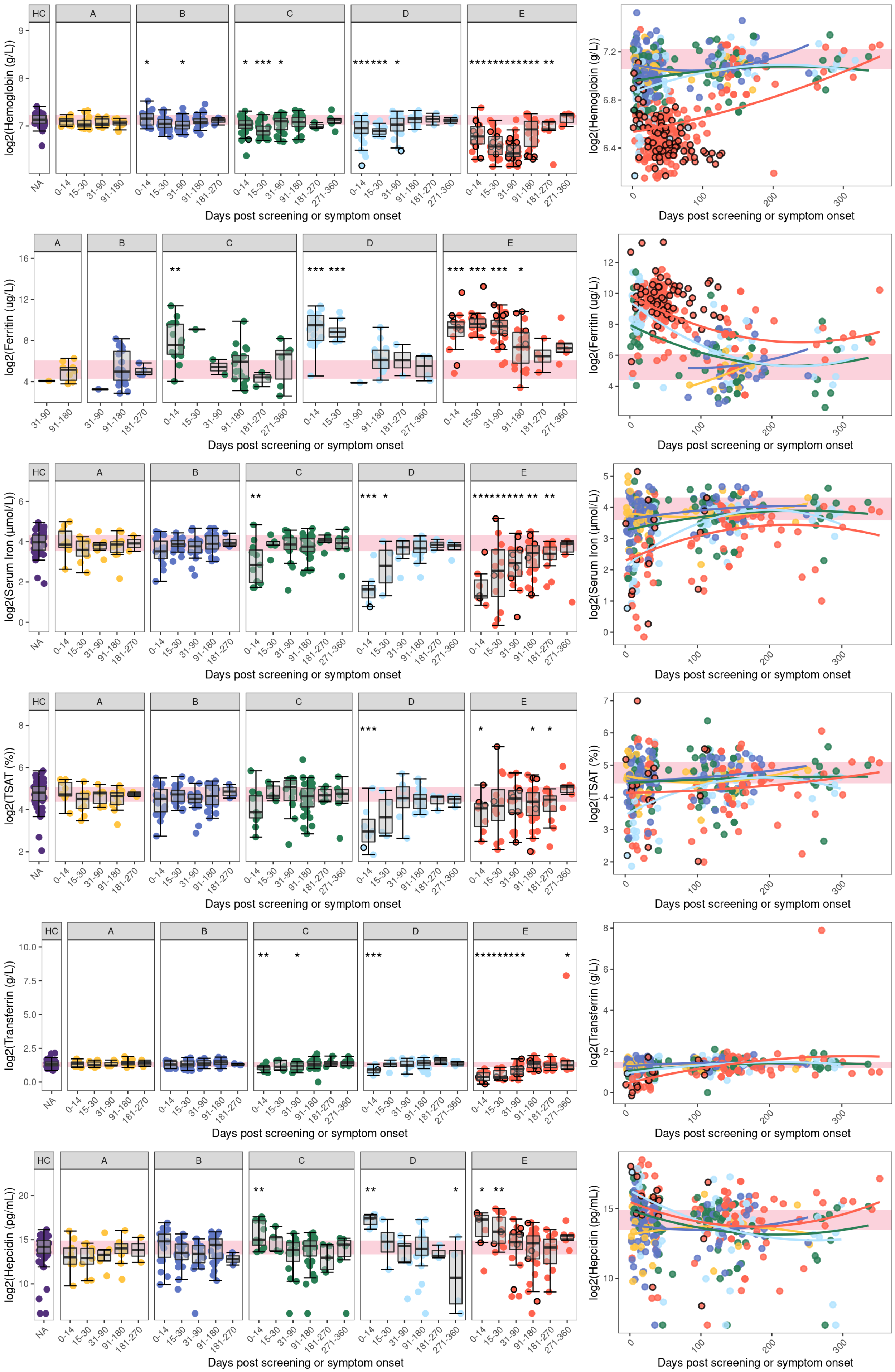
log2(Hemoglobin (g/L))
HC
A
B
C
D
E
NA
0-14
15-30
31-90
91-180
181-270
271-360
Days post screening or symptom onset
log2(Ferritin (ug/L))
log2(Serum Iron (µmol/L))
log2(TSAT (%))
log2(Transferrin (g/L))
log2(Hepcidin (pg/mL))

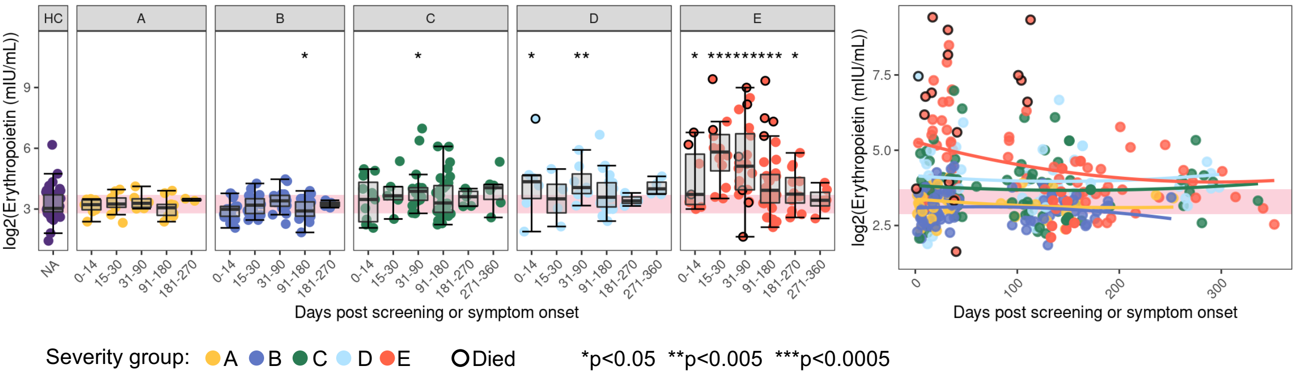

Supplementary Figure 3: Boxplots and longitudinal plots showing changes in serum iron and inflammatory measures over time for each COVID-19 severity group. Pink band indicates the interquartile range of the HCs, or samples collected from group A and B at >90 DPSO when HC measures were not obtained (ferritin). P-values are calculated by linear model of $\log_2$(measure) ~ severity group + age + sex (*p<0.05, **p<0.005, ***p<0.0005). Patients who passed away over the study period are rimmed in black. Boxplots show minimum, 25th percentile, median, 75th percentile and maximum, and outliers beyond 1.5 times the interquartile range.

Supplementary Figure 4

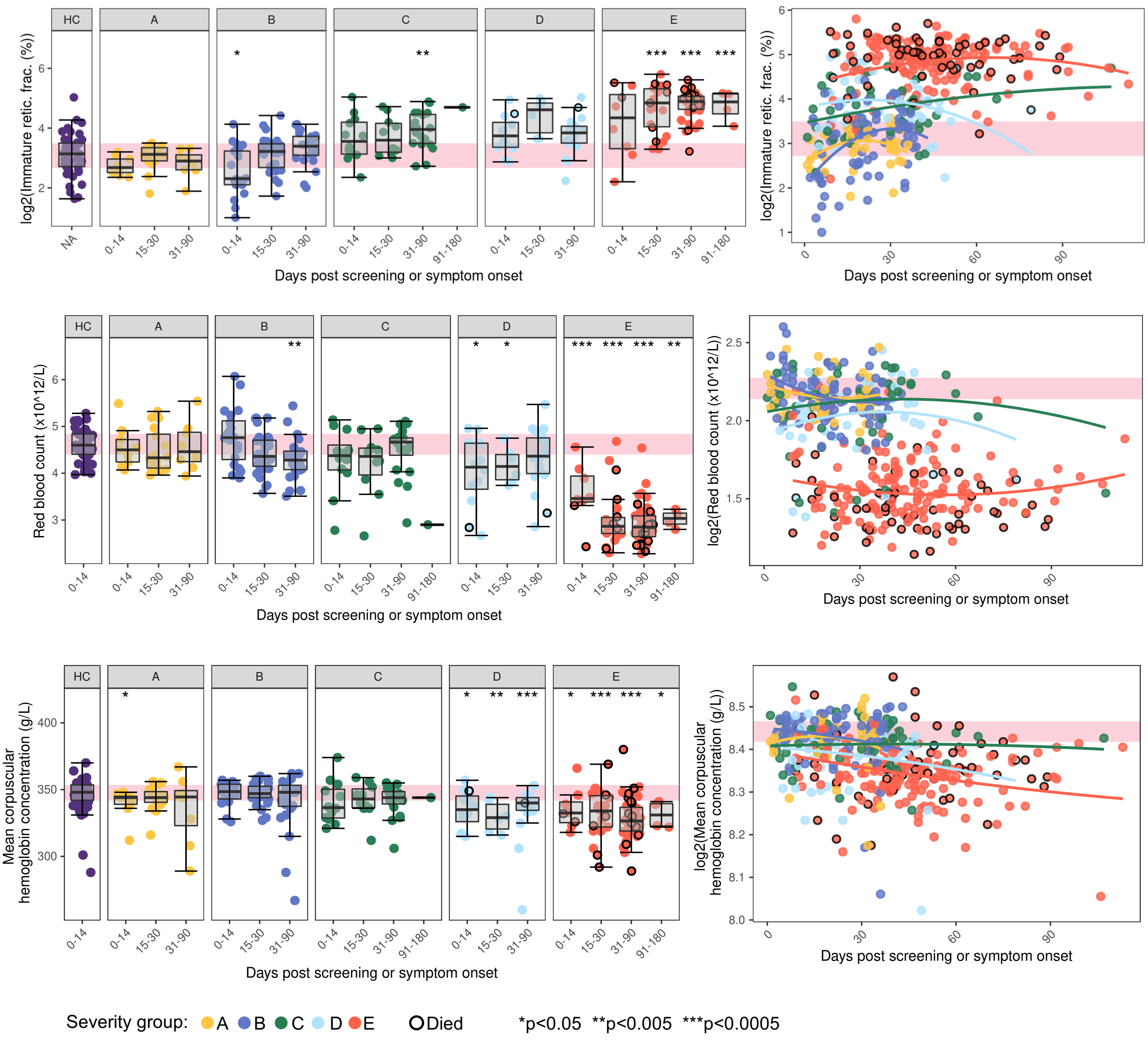

Supplementary Figure 4: Boxplots and longitudinal plots showing changes in hematological parameters over time for each COVID-19 severity group. Pink band indicates the interquartile range of the HCs. P-values are calculated by linear model of $\log_2$(count) ~ severity group + age + sex (*p<0.05, **p<0.005, ***p<0.0005). Patients who passed away over the study period are rimmed in black. Boxplots show minimum, 25th percentile, median, 75th percentile and maximum, and outliers beyond 1.5 times the interquartile range.

Supplementary Figure 5

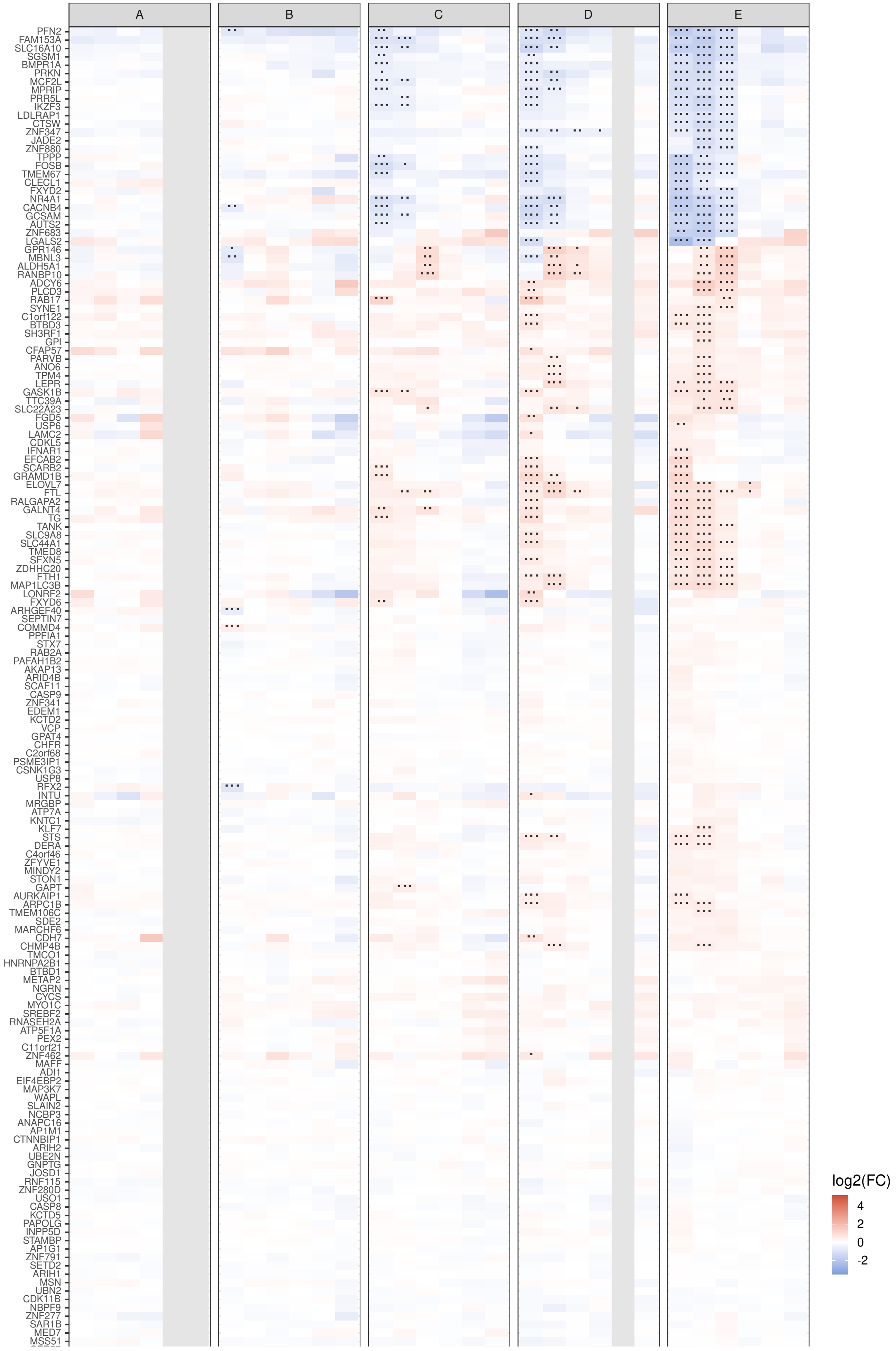

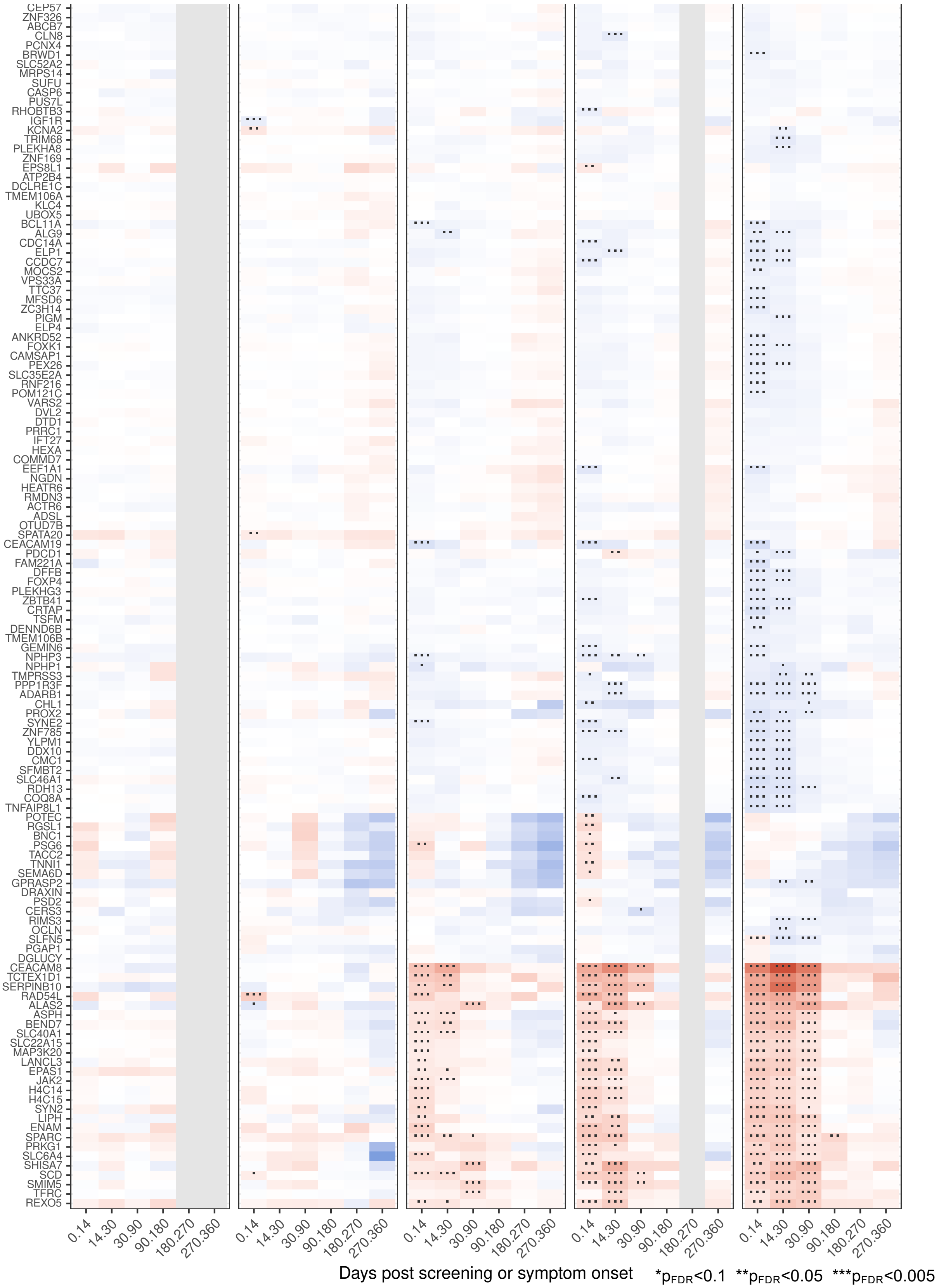

Supplementary Figure 5: Differential expression of 324 measured genes with high-quality conserved iron response elements (IREs) in their 3' or 5' UTR. Heat map showing log_2FC of each gene detail. Significantly differentially expressed genes from linear model fit ($P_{FDR}<0.1$, abs(log_2FC) >0.5) are indicated with asterisks: * $P_{FDR}<0.1$, ** $P_{FDR}<0.05$, ***$P_{FDR}<0.005$.

Supplementary Figure 6

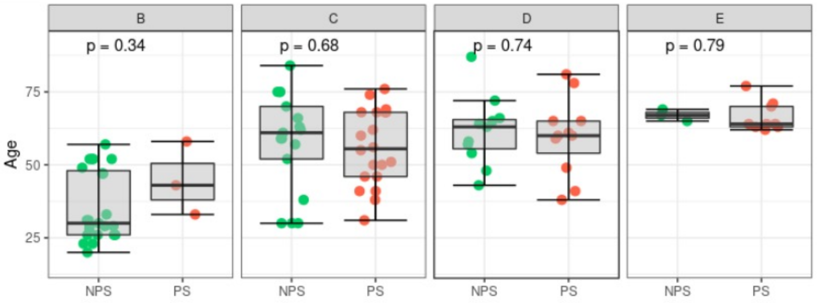

Supplementary Figure 6: Age comparison between individuals with PS or NPS at Q1, stratified by initial disease severity group. P-values calculated by T-test. PS = persisting symptoms, NPS = no persisting symptoms. Boxplots show minimum, 25th percentile, median, 75th percentile and maximum, and outliers beyond 1.5 times the interquartile range.

Supplementary Figure 7

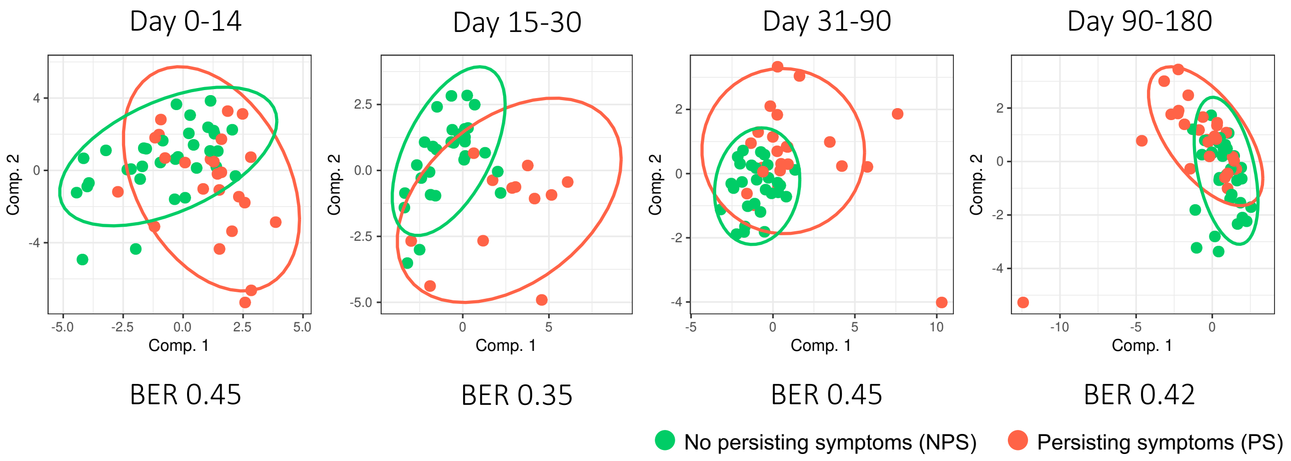

Supplementary Figure 7: Partial least squares discriminant analysis (PLS-DA) of "long-COVID" symptom groups conducted on immune-cell counts, serum parameters and reticulocyte data collected across sequential time windows from day 0-180. Classification performance based on 30 iterations of 4-fold cross validation is shown beneath cluster plots as balanced error rate (BER; minimum across 10 components). Low BER indicates high classification accuracy based on provided variables.

Supplementary Figure 8

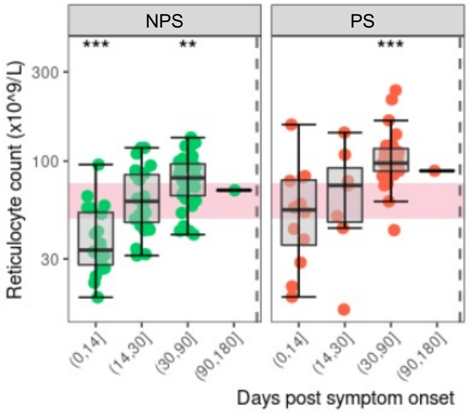

Supplementary Figure 8: Reticulocyte counts over time in patients reporting PS or NPS at Q1. P-values calculated by Wilcoxon test relative to HC (*p<0.05, **p<0.005, ***p<0.0005). Pink bar indicates the HC interquartile range. PS = persisting symptoms, NPS = no persisting symptoms. Boxplots show minimum, 25th percentile, median, 75th percentile and maximum, and outliers beyond 1.5 times the interquartile range.

Supplementary Figure 9

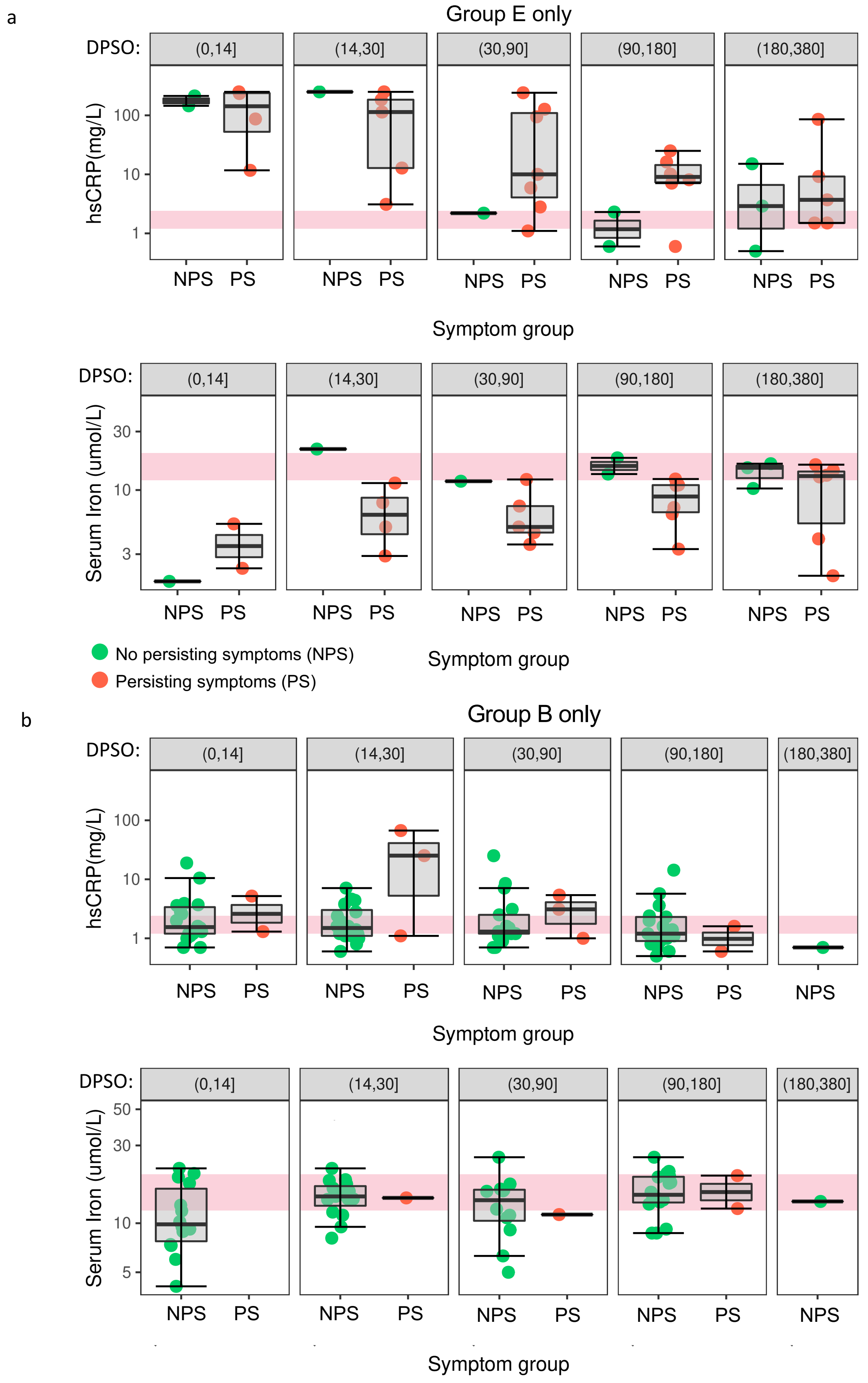

Supplementary Figure 9: Boxplots showing changes in CRP and serum iron over time in "long-COVID" symptom groups, within disease severity groups. Shown are **a,** group E (ventilated) patients and **b**, group B (mild symptomatic) patients who provided follow up questionnaire responses. Pink band indicates the interquartile range of the HCs. Y-axis is shown on a $\log_{10}$ scale. There was insufficient power to compare symptom groups by T-test. Boxplots show minimum, 25th percentile, median, 75th percentile and maximum, and outliers beyond 1.5 times the interquartile range.

Supplementary Figure 10

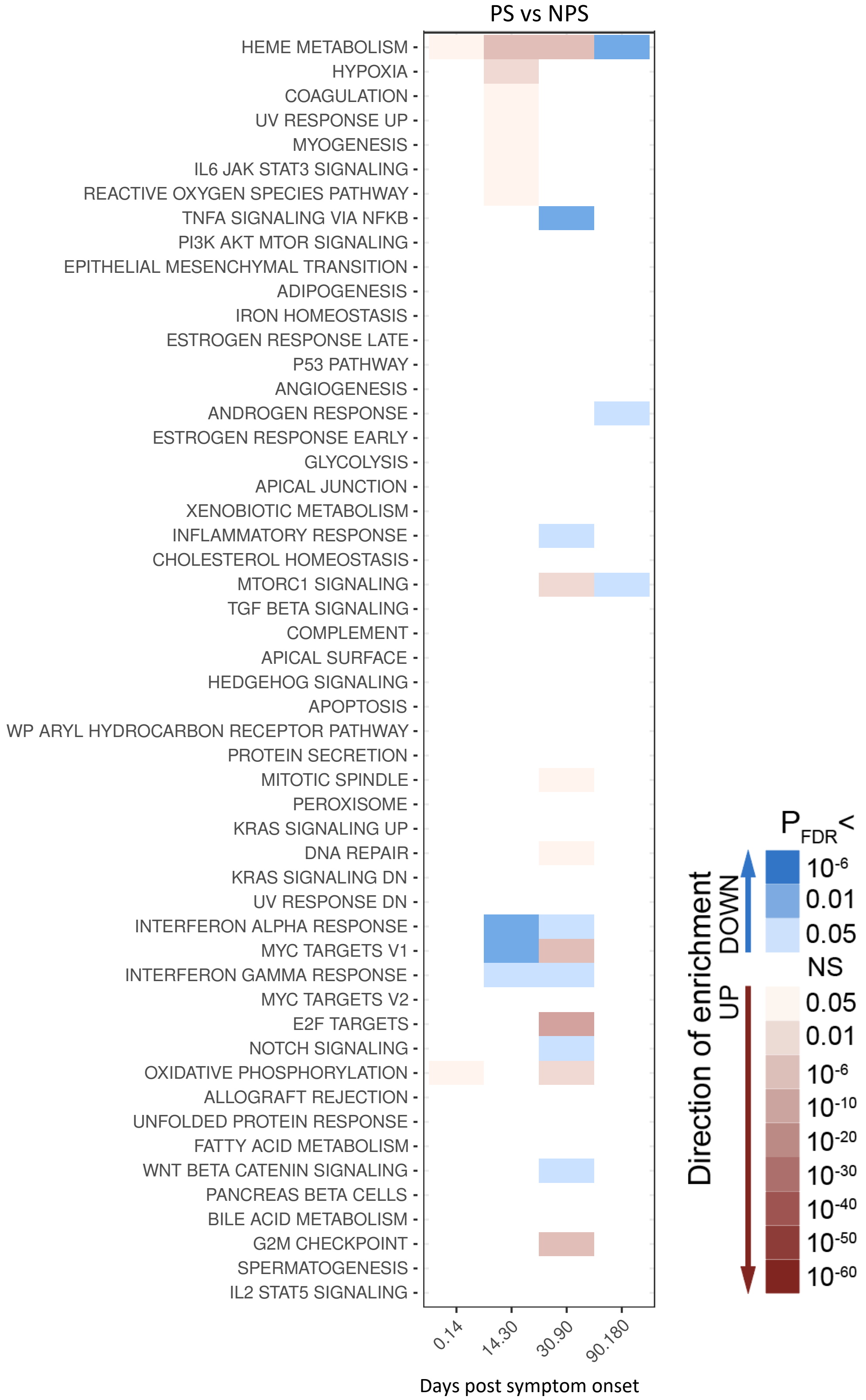

Supplementary Figure 10: GSEA using MSigDB HALLMARK gene-sets run on the log_2FC ranked gene lists from the comparison of PS vs NPS symptom groups, across time-windows Shade represents FDR adjusted p-value, with gene sets up- or down-regulated coloured red or blue respectively.

Supplementary Materials

Cambridge Institute of Therapeutic Immunology and Infectious Disease- National Institute of Health Research (CITIID-NIHR) COVID BioResource Collaboration

The members of the Cambridge Institute of Therapeutic Immunology and Infectious Disease-National Institute of Health Research (CITIID-NIHR) COVID BioResource Collaboration are Stephen Baker, John Bradley, Patrick Chinnery, Daniel Cooper, Gordon Dougan, Ian Goodfellow, Ravindra Gupta, Nathalie Kingston, Paul J. Lehner, Paul A. Lyons, Nicholas J. Matheson, Caroline Saunders, Kenneth G. C. Smith, Charlotte Summers, James Thaventhiran, M. Estee Torok, Mark R. Toshner, Michael P. Weekes, Gisele Alvio, Sharon Baker, Areti Bermperi, Karen Brookes, Ashlea Bucke, Jo Calder, Laura Canna, Cherry Crucusio, Isabel Cruz, Ranalie de Jesus, Katie Dempsey, Giovanni Di Stephano, Jason Domingo, Anne Elmer, Nicholas Gleadall, Julie Harris, Sarah Hewitt, Christoph Hess, Heather Jones, Sherly Jose, Jane Kennet, Yvonne King, Prasanti Kotagiri, Jenny Kourampa, Emily Li, Caroline McMahon, Anne Meadows, Vivien Mendoza, Criona O'Brien, Charmain Ocaya, Ciro Pasquale, Marlyn Perales, Jane Price, Rebecca Rastall, Carla Ribeiro, Jane Rowlands, Hélène Ruffieux, Valentina Ruffolo, Hugo Tordesillas, Phoebe Vargas, Bensi Vergese, Laura Watson, Jieniean Worsley, Julie-Ann Zerrudo, Laura Bergamashi, Ariana Betancourt, Georgie Bower, Ben Bullman, Chiara Cossetti, Aloka De Sa, Benjamin J. Dunmore, Maddie Epping, Stuart Fawke, Stefan Gräf, Richard Grenfell, Andrew Hinch, Josh Hodgson, Christopher Huang, Oisin Huhn, Kelvin Hunter, Isobel Jarvis, Emma Jones, Maša Josipović, Ekaterina Legchenko, Daniel Lewis, Joe Marsden, Jennifer Martin, Federica Mescia, Ciara O'Donnell, Ommar Omarjee, Marianne Perera, Linda Pointon, Nicole Pond, Nathan Richoz, Nika Romashova, Natalia Savoinykh, Rahul Sharma, Joy Shih, Mateusz Strezlecki, Rachel Sutcliffe, Tobias Tilly, Zhen Tong, Carmen Treacy, Lorinda Turner, Jennifer Wood, Marta Wylot, John Allison, Heather Biggs, John R. Bradley, Helen Butcher, Daniela Caputo, Matt Chandler, Patrick Chinnery, Debbie Clapham-Riley, Eleanor Dewhurst, Christian Fernandez, Anita Furlong, Barbara Graves, Jennifer Gray, Sabine Hein, Tasmin Ivers, Emma Le Gresley, Rachel Linger, Mary Kasanicki, Rebecca King, Nathalie Kingston, Sarah Meloy, Alexei Moulton, Francesca Muldoon, Nigel Ovington, Sofia Papadia, Christopher J. Penkett, Isabel Phelan, Venkatesh Ranganath, Roxana Paraschiv, Abigail Sage, Jennifer Sambrook, Ingrid Scholtes, Katherine Schon, Hannah Stark, Kathleen E. Stirrups, Paul Townsend, Neil Walker, Jennifer Webster, Mayurun Selvan, Petra, Polgarova, Sarah L. Caddy, Laura G. Caller, Yasmin Chaudhry, Martin D. Curran, Theresa Feltwell, Stewart Fuller, Iliana Georgana, Grant Hall, William L. Hamilton, Myra Hosmillo, Charlotte J. Houldcroft, Rhys Izuagbe, Aminu S. Jahun, Fahad A. Khokhar, Anna G. Kovalenko, Luke W. Meredith, Surendra Parmar, Malte L. Pinckert, Anna Yakovleva, Emily C. Horner, Lucy Booth, Alexander Ferreira, Rebecca Boston, Robert Hughes, Juan Carlos Yam Puc, Nonantzin Beristain-Covarrubias, Maria Rust, Thevinya Gurugama, Lihinya Gurugama, Thomas Mulroney, Sarah Spencer, Zhaleh Hosseini, Kate Williamson.

Supplementary Methods

Correction for age in the statistical comparison of COVID-19 severity groups and HCs, and PASC symptom groups with persisting symptoms (PS) or no persisting symptoms (NPS), was conducted by incorporation of age as a linear covariate in multivariate linear regression (as described in the **Methods**). To confirm the validity of this method of age correction, we tested the assumption that measured parameters varied linearly with respect to age. To test for an effect of age on measured clinical and cellular parameters in this study we assessed age associations in HCs, treating age as either a linear or quadratic predictor in a linear model:

$$\log_2(\text{measure}) \sim \text{age (or age + age}^2)$$

and in HC combined with late timepoint COVID-19 patient samples (beyond day 180 post-COVID-19 onset) with correction for acute disease severity group:

$$\log_2(\text{measure}) \sim \text{age + severity (or age + age}^2 + \text{severity)}$$

See **Table SM1** and **SM2** for results. We further tested for age associations (treating age as a linear or quadratic predictor) in per COVID-19 severity group-time window comparisons with HCs (Table **SM3** and **SM4**). Pearson correlations between age and each measured parameter in HC, and in HC plus COVID-19 severity group samples from acute disease (day 0-14) or late-stage disease (beyond day 180 DPSO) are shown in **Figure SM1** and **SM2**. For most parameters age correlations induced during acute disease are entirely driven by acute disease severity and cannot be detected at late timepoints. The remainder of age-dependent parameters exhibit age associations that are linear in nature and can be appropriately corrected by inclusion of a linear predictor in the model. Clinical parameters were compared by Wilcoxon test in an age matched subset of PS and NPS individuals >50 years of age (as shown in **Figure SM3**). Corresponding correlation plots are shown for PASC symptom groups in **Figure SM4**.

Table SM1: Age associations with measured clinical parameters in HCs and late stage COVID-19 with correction for acute disease severity

	HC only			HC and COVID-19 day 180-360		
	p.lm.age	p.lm.age1	p.lm.age2	p.lm.age	p.lm.age1	p.lm.age2
hsCRP	0.866	0.589	0.603	0.433	0.251	0.307
IL6	0.592	0.311	0.348	0.011	0.954	0.596
IL1b	0.345	0.919	0.795	0.850	0.444	0.457
IL10	0.031	0.085	0.166	0.820	0.360	0.373
TNFa	0.467	0.416	0.345	0.400	0.472	0.376
IFNg	0.547	0.68	0.604	0.334	0.275	0.198
HGB	0.434	0.749	0.844	0.968	0.243	0.237
Serum iron	0.640	0.706	0.761	0.926	0.829	0.840
TSAT	0.920	0.769	0.778	0.401	0.966	0.911
Transferrin	0.472	0.725	0.814	0.978	0.746	0.738
Hepcidin	0.715	0.500	0.533	0.421	0.304	0.370
Reticulocyte HGB	0.810	0.344	0.318			
Reticulocyte count	0.483	0.141	0.168		Not measured late	
Immmature retic. fraction	0.003	0.037	0.106			
Erythropoietin	0.296	0.511	0.399	0.548	0.624	0.699

p.lm.age = p-value for age effect in model log2(measure) ~ age (+ severity)
p.lm.age1 = p-value for linear age effect in model log2(measure) ~ age + age^2 (+ severity)
p.lm.age2 = p-value for quadratic age effect in model log2(measure) ~ age + age^2 (+ severity)

Table SM2: Age associations with measured immune cell counts in HCs and late stage COVID-19 with correction for acute disease severity

	HC only			HC and COVID-19 day 180-360		
	p.lm.age	p.lm.age1	p.lm.age2	p.lm.age	p.lm.age1	p.lm.age2
abs_CD3 +	0.156	0.666	0.499	0.988	0.233	0.225
abs_CD4+	0.373	0.588	0.485	0.831	0.351	0.325
CD4+/Naive	0.112	0.771	0.978	0.041	0.683	0.954
CD4+/CM	0.488	0.584	0.659	0.164	0.510	0.669
CD4+/EM	0.604	0.866	0.931	0.852	0.521	0.536
CD4+/EMRA	0.117	0.428	0.286	0.847	0.213	0.218
CD4+/NonNaive/HLADR+CD38+	0.332	0.929	0.807	0.045	0.936	0.661
CD4+/Tfh-like	0.627	0.619	0.559	0.113	0.573	0.397
CD25pCD127lo	0.174	0.301	0.407	0.788	0.020	0.016
abs_CD8+	0.038	0.266	0.139	0.334	0.766	0.893
CD8+/Naive	0.0004	0.88	0.621	5.32E-09	0.958	0.275
CD8+/CM	0.570	0.297	0.335	0.167	0.575	0.742
CD8+/EM	0.607	0.89	0.956	0.483	0.826	0.919
CD8+/EMRA	0.244	0.163	0.109	0.716	0.733	0.683
CD8+/NonNaive/HLADR+CD38+	0.798	0.237	0.247	0.958	0.362	0.35
gdTcells	0.004	0.230	0.475	0.011	0.010	0.030
gdTcells/TCR Vg9+ TCR-DV2high	0.002	0.086	0.223	0.00012	0.183	0.506
gdTcells/TCR Vg9+ TCR-DV2lo	0.017	0.474	0.748	0.113	0.149	0.233
abTcells/Q4: TCR Vg9- TCR-DV1-/MAIT	0.354	0.976	0.901	0.003	0.580	0.277
abs_NKT	0.855	0.984	0.992	0.606	0.514	0.453
abs_NK	0.449	0.966	0.866	0.209	0.754	0.921
CD19+/CD19 naive	0.545	0.540	0.602	0.059	0.581	0.373
CD19+/CD19 naive/Transitional	0.844	0.173	0.178	0.362	0.721	0.838
CD19+/IgD-CD27+ memory/CD24+CD38+	0.429	0.392	0.461	0.397	0.588	0.687
Plasmablasts	0.294	0.999	0.860	0.392	0.967	0.848
CD19+/MZ_like	0.326	0.536	0.430	0.135	0.538	0.376
pDC	0.727	0.263	0.234	0.744	0.370	0.334
mDC	0.483	0.234	0.275	0.474	0.563	0.643
classical	0.851	0.101	0.102	0.159	0.047	0.024
CD4activated/naive	0.070	0.704	0.498	0.001	0.916	0.625
CD8activated/naive	0.0002	0.691	0.800	3.97E-07	0.351	0.973

p.lm.age = p-value for age effect in model log2(measure) ~ age (+ severity)
p.lm.age1 = p-value for linear age effect in model log2(measure) ~ age + age^2 (+ severity)
p.lm.age2 = p-value for quadratic age effect in model log2(measure) ~ age + age^2 (+ severity)

Table SM3: COVID-19 severity group associations with clinical parameters under two models for age correction

Model 1: log2(measure) ~ severity group + age + sex **Model 2:** log2(measure) ~ severity group + age + age^2 + sex
Severity group effects are relative to healthy controls, tested independently for each severity (A-E) and time window

p.lm1.sev = p-value for Mod1 severity effect, **p.lm1.age** = p-value for Mod1 age effect, **p.lm2.sev** = p-value for Mod2 severity effect, **p.lm2.age** = p-value for Mod2 age effect, **p.lm2.age** = p-value for Mod2 age^2 effect. Stars are based on significance thresholding of p.lm1.sev as shown in primary figures. *p<0.05, **p<0.005, ***p<0.0005

measure	severity	time.window	p.lm1.sev	p.lm1.age	p.lm2.sev	p.lm2.age	p.lm2.age2	p.stars
hsCRP	A	(0,14]	0.0934	0.239	0.098	0.681	0.54	
hsCRP	A	(14,30]	0.535	0.0477	0.542	0.8	0.937	
hsCRP	A	(30,90]	0.785	0.683	0.8	0.64	0.684	
hsCRP	A	(90,180]	0.29	0.126	0.314	0.567	0.403	
hsCRP	B	(0,14]	0.993	0.44	0.995	0.999	0.898	
hsCRP	B	(14,30]	0.684	0.64	0.736	0.522	0.568	
hsCRP	B	(30,90]	0.71	0.754	0.69	0.841	0.799	
hsCRP	B	(90,180]	0.0649	0.398	0.0785	0.64	0.739	
hsCRP	B	(180,270]	0.0311	0.383	0.0429	0.418	0.498	*
hsCRP	C	(0,14]	5.96E-10	0.0731	1.40E-09	0.821	0.537	***
hsCRP	C	(14,30]	2.00E-05	0.71	2.25E-05	0.645	0.69	***
hsCRP	C	(30,90]	0.455	0.065	0.458	0.431	0.254	
hsCRP	C	(90,180]	0.83	0.164	0.86	0.608	0.414	
hsCRP	C	(180,270]	0.222	0.664	0.232	0.729	0.782	
hsCRP	C	(270,360]	0.682	0.702	0.681	0.528	0.565	
hsCRP	D	(0,14]	2.09E-24	0.202	4.25E-25	0.0632	0.0284	***
hsCRP	D	(14,30]	7.99E-12	0.803	4.21E-11	0.555	0.509	***
hsCRP	D	(30,90]	0.00323	0.451	0.00429	0.795	0.667	**
hsCRP	D	(90,180]	0.542	0.553	0.53	0.682	0.772	
hsCRP	D	(180,270]	0.307	0.959	0.261	0.393	0.391	
hsCRP	D	(270,360]	0.332	0.925	0.695	0.291	0.291	
hsCRP	E	(0,14]	4.38E-18	0.462	3.58E-17	0.784	0.644	***
hsCRP	E	(14,30]	1.81E-16	0.237	2.33E-16	0.626	0.448	***
hsCRP	E	(30,90]	1.73E-19	0.0618	1.41E-19	0.058	0.0225	***
hsCRP	E	(90,180]	8.63E-05	0.538	0.000102	0.301	0.246	***
hsCRP	E	(180,270]	0.00848	0.538	0.00899	0.782	0.863	*
hsCRP	E	(270,360]	0.278	0.824	0.202	0.415	0.43	
IL6	A	(0,14]	0.00819	0.0259	0.0089	0.798	0.525	*
IL6	A	(14,30]	0.0868	0.0109	0.0915	0.778	0.879	
IL6	A	(30,90]	0.0632	0.362	0.0689	0.441	0.529	
IL6	A	(90,180]	0.0501	0.0543	0.057	0.398	0.238	
IL6	B	(0,14]	0.0263	0.646	0.0339	0.46	0.501	*
IL6	B	(14,30]	0.0547	0.0768	0.0767	0.109	0.183	
IL6	B	(30,90]	0.221	0.223	0.279	0.23	0.309	
IL6	B	(90,180]	0.38	0.681	0.455	0.427	0.461	
IL6	B	(180,270]	0.189	0.294	0.144	0.209	0.272	
IL6	C	(0,14]	2.81E-10	0.0632	2.68E-09	0.905	0.63	***
IL6	C	(14,30]	4.31E-07	0.444	7.60E-07	0.413	0.488	***
IL6	C	(30,90]	0.0174	0.0376	0.0158	0.15	0.0647	*
IL6	C	(90,180]	7.83E-06	0.191	9.61E-06	0.434	0.283	***
IL6	C	(180,270]	0.00503	0.857	0.00442	0.283	0.289	*
IL6	C	(270,360]	0.0121	0.718	0.0124	0.298	0.32	*
IL6	D	(0,14]	3.63E-25	0.642	6.45E-25	0.101	0.0725	***
IL6	D	(14,30]	4.54E-09	0.712	6.83E-08	0.428	0.371	***
IL6	D	(30,90]	0.00392	0.0843	0.0067	0.141	0.0594	**
IL6	D	(90,180]	0.0731	0.029	0.1	0.0645	0.0178	
IL6	D	(180,270]	0.1	0.411	0.119	0.603	0.696	
IL6	D	(270,360]	0.355	0.607	0.272	0.479	0.531	
IL6	E	(0,14]	1.57E-23	0.692	1.15E-22	0.447	0.386	***
IL6	E	(14,30]	1.40E-14	0.927	2.19E-14	0.743	0.723	***
IL6	E	(30,90]	1.26E-18	0.245	1.37E-18	0.0767	0.0434	***
IL6	E	(90,180]	1.22E-08	0.918	1.63E-08	0.461	0.465	***
IL6	E	(180,270]	0.000161	0.0974	0.000222	0.496	0.324	***
IL6	E	(270,360]	2.87E-06	0.565	2.99E-06	0.391	0.439	***
IL1b	A	(0,14]	0.117	0.351	0.121	0.94	0.818	
IL1b	A	(14,30]	0.87	0.248	0.862	0.976	0.823	
IL1b	A	(30,90]	0.162	0.385	0.162	0.824	0.711	
IL1b	A	(90,180]	0.0181	0.889	0.0202	0.692	0.705	*
IL1b	B	(0,14]	0.0281	0.34	0.0255	0.682	0.566	*
IL1b	B	(14,30]	0.525	0.509	0.475	0.529	0.456	
IL1b	B	(30,90]	0.187	0.29	0.173	0.757	0.626	
IL1b	B	(90,180]	0.0403	0.166	0.044	0.796	0.977	*
IL1b	B	(180,270]	0.447	0.5	0.394	0.556	0.478	
IL1b	C	(0,14]	0.0209	0.408	0.0433	0.67	0.554	*
IL1b	C	(14,30]	0.0155	0.358	0.00701	0.275	0.202	*
IL1b	C	(30,90]	0.734	0.402	0.735	0.0115	0.00661	
IL1b	C	(90,180]	0.000592	0.49	7.00E-04	0.233	0.282	**
IL1b	C	(180,270]	0.848	0.597	0.857	0.772	0.839	
IL1b	C	(270,360]	0.554	0.391	0.557	0.893	0.777	

IL1b	D	(0,14]	1.56E-05	0.566	1.49E-05	0.478	0.386	***
IL1b	D	(14,30]	3.75E-05	0.187	1.37E-05	0.237	0.132	***
IL1b	D	(30,90]	0.267	0.292	0.402	0.0236	0.0111	
IL1b	D	(90,180]	0.0386	0.324	0.0535	0.0143	0.00665	*
IL1b	D	(180,270]	0.599	0.413	0.576	0.858	0.75	
IL1b	D	(270,360]	0.81	0.414	0.742	0.912	0.796	
IL1b	E	(0,14]	2.10E-05	0.738	4.47E-05	0.63	0.675	***
IL1b	E	(14,30]	1.69E-05	0.576	1.80E-05	0.442	0.364	***
IL1b	E	(30,90]	7.03E-06	0.161	1.02E-05	0.426	0.282	***
IL1b	E	(90,180]	4.55E-06	0.429	5.33E-06	0.244	0.185	***
IL1b	E	(180,270]	0.00277	0.345	0.00221	0.376	0.287	**
IL1b	E	(270,360]	0.0225	0.761	0.0225	0.736	0.695	*
IL10	A	(0,14]	0.159	0.692	0.159	0.662	0.611	
IL10	A	(14,30]	0.0471	0.845	0.0491	0.978	0.952	*
IL10	A	(30,90]	0.218	0.0191	0.236	0.0793	0.166	
IL10	A	(90,180]	0.00238	0.908	0.00268	0.926	0.94	**
IL10	B	(0,14]	0.0684	0.882	0.0734	0.976	0.956	
IL10	B	(14,30]	0.0711	0.337	0.0729	0.786	0.907	
IL10	B	(30,90]	0.649	0.0208	0.535	0.101	0.2	
IL10	B	(90,180]	0.000727	0.159	0.000587	0.316	0.436	**
IL10	B	(180,270]	0.778	0.0166	0.944	0.0765	0.163	
IL10	C	(0,14]	1.94E-09	0.774	1.88E-08	0.572	0.528	***
IL10	C	(14,30]	2.08E-06	0.843	2.87E-05	0.218	0.197	***
IL10	C	(30,90]	0.281	0.727	0.264	0.00704	0.00746	
IL10	C	(90,180]	1.62E-06	0.0214	1.49E-07	2.73E-05	2.11E-06	***
IL10	C	(180,270]	0.000404	0.153	0.00036	0.0217	0.0369	***
IL10	C	(270,360]	0.867	0.02	0.856	0.0515	0.115	
IL10	D	(0,14]	2.58E-15	0.49	5.52E-14	0.552	0.655	***
IL10	D	(14,30]	5.19E-16	0.0191	1.41E-15	0.514	0.228	***
IL10	D	(30,90]	0.103	0.697	0.143	0.14	0.153	
IL10	D	(90,180]	4.87E-05	1	7.07E-05	0.0996	0.092	***
IL10	D	(180,270]	0.778	0.0204	0.628	0.0697	0.147	
IL10	D	(270,360]	0.00161	0.392	0.0137	0.289	0.355	**
IL10	E	(0,14]	3.94E-17	0.993	2.20E-16	0.247	0.235	***
IL10	E	(14,30]	3.58E-12	0.853	4.69E-12	0.528	0.545	***
IL10	E	(30,90]	7.37E-17	0.00235	4.40E-17	0.0422	0.008	***
IL10	E	(90,180]	3.30E-08	0.547	4.40E-08	0.776	0.691	***
IL10	E	(180,270]	1.95E-07	0.164	2.28E-07	0.831	0.643	***
IL10	E	(270,360]	0.322	0.0884	0.649	0.0189	0.0357	
TNFa	A	(0,14]	0.438	0.481	0.441	0.974	0.933	
TNFa	A	(14,30]	0.00246	0.601	0.00283	0.689	0.75	**
TNFa	A	(30,90]	0.0628	0.94	0.06	0.462	0.448	
TNFa	A	(90,180]	0.202	0.921	0.212	0.808	0.792	
TNFa	B	(0,14]	0.222	0.402	0.271	0.429	0.346	
TNFa	B	(14,30]	0.0638	0.968	0.0527	0.378	0.375	
TNFa	B	(30,90]	0.591	0.849	0.481	0.211	0.195	
TNFa	B	(90,180]	0.117	0.397	0.0891	0.399	0.318	
TNFa	B	(180,270]	0.552	0.462	0.44	0.287	0.23	
TNFa	C	(0,14]	0.288	0.472	0.512	0.305	0.237	
TNFa	C	(14,30]	0.433	0.775	0.472	0.938	0.978	
TNFa	C	(30,90]	0.0111	0.661	0.0117	0.686	0.623	*
TNFa	C	(90,180]	0.519	0.507	0.548	0.429	0.343	
TNFa	C	(180,270]	0.207	0.712	0.208	0.856	0.804	
TNFa	C	(270,360]	0.387	0.565	0.393	0.711	0.635	
TNFa	D	(0,14]	0.000498	0.723	0.000744	0.828	0.758	***
TNFa	D	(14,30]	0.000501	0.407	0.000567	0.683	0.546	**
TNFa	D	(30,90]	0.00244	0.878	0.00378	0.334	0.307	**
TNFa	D	(90,180]	0.494	0.779	0.571	0.216	0.185	
TNFa	D	(180,270]	0.0169	0.376	0.0127	0.393	0.311	*
TNFa	D	(270,360]	0.00789	0.47	0.00813	0.594	0.502	*
TNFa	E	(0,14]	1.35E-05	0.492	1.63E-05	0.789	0.668	***
TNFa	E	(14,30]	1.38E-07	0.188	1.32E-07	0.328	0.197	***
TNFa	E	(30,90]	0.000133	0.116	0.000184	0.477	0.306	***
TNFa	E	(90,180]	0.0389	0.579	0.0435	0.259	0.213	*
TNFa	E	(180,270]	0.0407	0.356	0.0365	0.539	0.432	*
TNFa	E	(270,360]	0.0125	0.529	0.0099	0.532	0.46	*
IFNg	B	(0,14]	0.432	0.604	0.439	0.942	0.99	
IFNg	B	(30,90]	0.33	0.572	0.339	0.937	0.99	
IFNg	C	(0,14]	0.000737	0.099	6.19E-05	0.0348	0.0138	**
IFNg	D	(0,14]	0.00123	0.596	0.000599	0.28	0.217	**
IFNg	D	(14,30]	8.99E-05	0.0143	1.16E-06	0.00991	0.00144	***
IFNg	E	(0,14]	2.00E-04	0.42	0.00058	0.221	0.152	***
IFNg	E	(14,30]	0.0123	0.741	0.0126	0.485	0.52	*
IFNg	E	(30,90]	0.276	0.443	0.305	0.54	0.442	
IFNg	E	(90,180]	0.297	0.804	0.315	0.456	0.423	

HGB	A	(0,14]	0.229	0.106	0.235	0.257	0.38	
HGB	A	(14,30]	0.709	0.142	0.702	0.628	0.805	
HGB	A	(30,90]	0.585	0.127	0.596	0.679	0.867	
HGB	A	(90,180]	0.854	0.176	0.855	0.827	0.993	
HGB	B	(0,14]	0.0297	0.0361	0.0296	0.526	0.764	*
HGB	B	(14,30]	0.309	0.139	0.302	0.984	0.825	
HGB	B	(30,90]	0.0475	0.0498	0.0529	0.718	0.966	*
HGB	B	(90,180]	0.49	0.175	0.543	0.789	0.618	
HGB	B	(180,270]	0.862	0.112	0.871	0.755	0.958	
HGB	C	(0,14]	0.00938	0.0186	0.0163	0.641	0.326	*
HGB	C	(14,30]	6.79E-05	0.225	7.80E-05	0.587	0.741	***
HGB	C	(30,90]	0.043	0.279	0.0447	0.762	0.61	*
HGB	C	(90,180]	0.293	0.53	0.301	0.957	0.948	
HGB	C	(180,270]	0.27	0.0743	0.277	0.902	0.855	
HGB	C	(270,360]	0.387	0.196	0.386	0.415	0.543	
HGB	D	(0,14]	0.000451	0.256	0.000473	0.589	0.772	***
HGB	D	(14,30]	0.000197	0.0434	0.00037	0.635	0.977	***
HGB	D	(30,90]	0.0116	0.125	0.0113	0.452	0.667	*
HGB	D	(90,180]	0.626	0.0472	0.681	0.491	0.787	
HGB	D	(270,360]	0.523	0.127	0.489	0.585	0.776	
HGB	E	(0,14]	1.22E-06	0.69	5.87E-07	0.148	0.164	***
HGB	E	(14,30]	1.44E-17	0.862	1.08E-17	0.0715	0.06	***
HGB	E	(30,90]	8.92E-32	0.2	1.97E-31	0.246	0.156	***
HGB	E	(90,180]	3.77E-06	0.774	3.36E-06	0.194	0.169	***
HGB	E	(180,270]	0.00273	0.749	0.00109	0.127	0.135	**
HGB	E	(270,360]	0.718	0.0795	0.701	0.889	0.879	
FERRITIN	C	(0,14]	0.000666	0.652	0.000414	0.346	0.286	**
FERRITIN	C	(90,180]	0.762	0.151	0.723	0.635	0.41	
FERRITIN	C	(180,270]	0.122	0.964	0.23	0.174	0.17	
FERRITIN	C	(270,360]	0.883	0.728	0.817	0.77	0.81	
FERRITIN	D	(0,14]	5.89E-07	0.686	5.89E-07	0.551	0.468	***
FERRITIN	D	(14,30]	6.84E-06	0.751	1.09E-05	0.506	0.545	***
FERRITIN	D	(90,180]	0.948	0.335	0.744	0.496	0.336	
FERRITIN	D	(270,360]	0.123	0.727	0.736	0.361	0.386	
FERRITIN	E	(0,14]	0.000284	0.822	0.000108	0.176	0.136	***
FERRITIN	E	(14,30]	5.48E-09	0.399	7.92E-09	0.548	0.676	***
FERRITIN	E	(30,90]	5.83E-10	0.62	7.68E-10	0.0955	0.109	***
FERRITIN	E	(90,180]	0.0199	0.445	0.0157	0.319	0.236	*
FERRITIN	E	(180,270]	0.635	0.596	0.71	0.853	0.911	
FERRITIN	E	(270,360]	0.0641	0.612	0.00531	0.0357	0.0272	
Iron	A	(0,14]	0.696	0.617	0.682	0.753	0.815	
Iron	A	(14,30]	0.246	0.703	0.26	0.841	0.889	
Iron	A	(30,90]	0.788	0.617	0.799	0.722	0.783	
Iron	A	(90,180]	0.536	0.133	0.544	0.959	0.759	
Iron	B	(0,14]	0.0561	0.629	0.0704	0.55	0.599	
Iron	B	(14,30]	0.879	0.601	0.91	0.738	0.799	
Iron	B	(30,90]	0.439	0.898	0.435	0.854	0.836	
Iron	B	(90,180]	0.605	0.844	0.64	0.831	0.803	
Iron	B	(180,270]	0.749	0.548	0.792	0.651	0.719	
Iron	C	(0,14]	0.000937	0.138	0.00122	0.917	0.677	**
Iron	C	(14,30]	0.987	0.64	0.99	0.561	0.609	
Iron	C	(30,90]	0.559	0.464	0.54	0.448	0.526	
Iron	C	(90,180]	0.294	0.678	0.305	0.721	0.651	
Iron	C	(180,270]	0.667	0.463	0.672	0.89	0.99	
Iron	C	(270,360]	0.826	0.675	0.829	0.489	0.529	
Iron	D	(0,14]	6.97E-10	0.465	1.07E-09	0.953	0.946	***
Iron	D	(14,30]	0.00707	0.507	0.0081	0.985	0.911	*
Iron	D	(30,90]	0.203	0.288	0.22	0.69	0.539	
Iron	D	(90,180]	0.286	0.319	0.29	0.798	0.966	
Iron	D	(180,270]	0.992	0.603	0.946	0.624	0.682	
Iron	D	(270,360]	0.513	0.621	0.408	0.537	0.59	
Iron	E	(0,14]	2.01E-08	0.779	3.18E-08	0.505	0.531	***
Iron	E	(14,30]	7.85E-05	0.816	6.64E-05	0.324	0.296	***
Iron	E	(30,90]	8.46E-05	0.192	0.000106	0.855	0.664	***
Iron	E	(90,180]	0.000728	0.698	0.000842	0.485	0.523	**
Iron	E	(180,270]	0.00476	0.982	0.00462	0.598	0.589	**
Iron	E	(270,360]	0.0838	0.542	0.0693	0.472	0.53	
TSAT	A	(0,14]	0.373	0.876	0.364	0.764	0.781	
TSAT	A	(14,30]	0.559	0.973	0.578	0.855	0.857	
TSAT	A	(30,90]	0.874	0.869	0.865	0.736	0.753	
TSAT	A	(90,180]	0.693	0.413	0.698	0.991	0.9	
TSAT	B	(0,14]	0.381	0.958	0.427	0.625	0.626	
TSAT	B	(14,30]	0.644	0.983	0.619	0.768	0.763	
TSAT	B	(30,90]	0.637	0.671	0.633	0.837	0.889	
TSAT	B	(90,180]	0.752	0.847	0.771	0.886	0.91	
TSAT	B	(180,270]	0.528	0.803	0.572	0.684	0.711	

TSAT	C	(0,14]	0.0746	0.347	0.0834	0.919	0.765	
TSAT	C	(14,30]	0.515	0.936	0.5	0.577	0.581	
TSAT	C	(30,90]	0.578	0.612	0.597	0.585	0.645	
TSAT	C	(90,180]	0.427	0.865	0.431	0.946	0.973	
TSAT	C	(180,270]	0.936	0.759	0.945	0.794	0.833	
TSAT	C	(270,360]	0.8	0.891	0.802	0.523	0.533	
TSAT	D	(0,14]	1.43E-05	0.716	1.78E-05	0.947	0.997	***
TSAT	D	(14,30]	0.0637	0.564	0.0717	0.752	0.663	
TSAT	D	(30,90]	0.449	0.418	0.478	0.546	0.439	
TSAT	D	(90,180]	0.227	0.823	0.211	0.505	0.526	
TSAT	D	(180,270]	0.629	0.888	0.684	0.62	0.631	
TSAT	D	(270,360]	0.408	0.834	0.362	0.661	0.683	
TSAT	E	(0,14]	0.0158	0.985	0.0129	0.496	0.486	*
TSAT	E	(14,30]	0.126	0.795	0.116	0.435	0.399	
TSAT	E	(30,90]	0.205	0.217	0.216	0.942	0.758	
TSAT	E	(90,180]	0.0138	0.774	0.0152	0.566	0.596	*
TSAT	E	(180,270]	0.0298	0.563	0.0275	0.582	0.508	*
TSAT	E	(270,360]	0.409	0.836	0.469	0.787	0.811	
Transferrin	A	(0,14]	0.231	0.435	0.246	0.753	0.853	
Transferrin	A	(14,30]	0.281	0.447	0.297	0.777	0.875	
Transferrin	A	(30,90]	0.342	0.525	0.35	0.841	0.923	
Transferrin	A	(90,180]	0.795	0.203	0.799	0.962	0.869	
Transferrin	B	(0,14]	0.0831	0.325	0.0925	0.765	0.887	
Transferrin	B	(14,30]	0.175	0.299	0.187	0.805	0.934	
Transferrin	B	(30,90]	0.71	0.158	0.726	0.66	0.828	
Transferrin	B	(90,180]	0.448	0.442	0.456	0.893	0.994	
Transferrin	B	(180,270]	0.397	0.603	0.399	0.852	0.918	
Transferrin	C	(0,14]	0.000746	0.222	0.000938	0.972	0.827	**
Transferrin	C	(14,30]	0.196	0.409	0.199	0.998	0.891	
Transferrin	C	(30,90]	0.0245	0.732	0.0258	0.881	0.929	*
Transferrin	C	(90,180]	0.977	0.203	0.997	0.721	0.524	
Transferrin	C	(180,270]	0.346	0.421	0.344	0.899	0.788	
Transferrin	C	(270,360]	0.535	0.561	0.54	0.901	0.98	
Transferrin	D	(0,14]	0.000271	0.495	0.000317	0.832	0.924	***
Transferrin	D	(14,30]	0.258	0.983	0.225	0.271	0.26	
Transferrin	D	(30,90]	0.686	0.935	0.645	0.314	0.313	
Transferrin	D	(90,180]	0.282	0.138	0.234	0.34	0.191	
Transferrin	D	(180,270]	0.169	0.428	0.182	0.974	0.92	
Transferrin	D	(270,360]	0.347	0.585	0.515	0.666	0.733	
Transferrin	E	(0,14]	2.09E-09	0.431	9.42E-09	0.829	0.717	***
Transferrin	E	(14,30]	4.38E-11	0.897	5.37E-11	0.482	0.489	***
Transferrin	E	(30,90]	6.84E-05	0.353	8.59E-05	0.775	0.64	***
Transferrin	E	(90,180]	0.641	0.123	0.654	0.925	0.704	
Transferrin	E	(180,270]	0.682	0.135	0.709	0.903	0.695	
Transferrin	E	(270,360]	0.0143	0.839	0.0159	0.747	0.77	*
Hepcidin	A	(0,14]	0.913	0.911	0.944	0.714	0.724	
Hepcidin	A	(14,30]	0.8	0.728	0.755	0.524	0.557	
Hepcidin	A	(30,90]	0.853	0.88	0.868	0.666	0.68	
Hepcidin	A	(90,180]	0.377	0.593	0.383	0.998	0.927	
Hepcidin	B	(0,14]	0.0614	0.319	0.0851	0.268	0.337	
Hepcidin	B	(14,30]	0.693	0.517	0.794	0.326	0.373	
Hepcidin	B	(30,90]	0.873	0.487	0.787	0.148	0.176	
Hepcidin	B	(90,180]	0.208	0.65	0.266	0.367	0.402	
Hepcidin	B	(180,270]	0.682	0.832	0.745	0.57	0.589	
Hepcidin	C	(0,14]	0.00438	0.659	0.00453	0.685	0.746	**
Hepcidin	C	(14,30]	0.137	0.778	0.149	0.558	0.585	
Hepcidin	C	(30,90]	0.412	0.702	0.42	0.843	0.896	
Hepcidin	C	(90,180]	0.666	0.249	0.665	0.914	0.892	
Hepcidin	C	(180,270]	0.413	0.92	0.439	0.439	0.442	
Hepcidin	C	(270,360]	0.715	0.828	0.717	0.725	0.749	
Hepcidin	D	(0,14]	0.000834	0.835	0.000925	0.571	0.59	**
Hepcidin	D	(14,30]	0.241	0.78	0.202	0.215	0.188	
Hepcidin	D	(30,90]	0.5	0.58	0.556	0.104	0.0783	
Hepcidin	D	(90,180]	0.656	0.595	0.64	0.846	0.753	
Hepcidin	D	(180,270]	0.968	0.826	0.971	0.569	0.589	
Hepcidin	D	(270,360]	0.0181	0.925	0.0949	0.374	0.376	*
Hepcidin	E	(0,14]	0.029	0.571	0.0389	0.994	0.912	*
Hepcidin	E	(14,30]	0.00447	0.89	0.00436	0.535	0.545	**
Hepcidin	E	(30,90]	0.7	0.858	0.684	0.69	0.66	
Hepcidin	E	(90,180]	0.602	0.633	0.613	0.83	0.76	
Hepcidin	E	(180,270]	0.55	0.346	0.562	0.772	0.9	
Hepcidin	E	(270,360]	0.157	0.975	0.115	0.421	0.411	
RET_HE	A	(0,14]	0.706	0.61	0.715	0.241	0.27	
RET_HE	A	(14,30]	0.577	0.669	0.622	0.195	0.214	
RET_HE	A	(30,90]	0.595	0.615	0.639	0.22	0.245	

RET_HE	B	(0,14]	0.112	0.443	0.136	0.557	0.472	
RET_HE	B	(14,30]	0.7	0.55	0.64	0.647	0.575	
RET_HE	B	(30,90]	0.483	0.654	0.4	0.381	0.337	
RET_HE	C	(0,14]	0.313	0.959	0.326	0.887	0.876	
RET_HE	C	(14,30]	0.736	0.944	0.965	0.402	0.386	
RET_HE	C	(30,90]	0.0231	0.172	0.0272	0.477	0.337	*
RET_HE	D	(0,14]	0.000166	0.909	0.000554	0.966	0.945	***
RET_HE	D	(14,30]	0.0326	0.788	0.149	0.754	0.707	*
RET_HE	D	(30,90]	0.512	0.495	0.55	0.785	0.667	
RET_HE	E	(0,14]	8.50E-06	0.858	1.12E-05	0.711	0.729	***
RET_HE	E	(14,30]	0.00258	0.133	0.00235	0.36	0.205	**
RET_HE	E	(30,90]	2.58E-05	0.233	4.48E-05	0.363	0.251	***
RET_HE	E	(90,180]	0.0333	0.644	0.0166	0.11	0.089	*
RET_A	A	(0,14]	0.0971	0.585	0.0928	0.054	0.0624	
RET_A	A	(14,30]	0.755	0.663	0.691	0.119	0.132	
RET_A	A	(30,90]	0.809	0.397	0.744	0.117	0.147	
RET_A	B	(0,14]	3.43E-06	0.916	3.61E-06	0.592	0.599	***
RET_A	B	(14,30]	0.788	0.649	0.661	0.252	0.278	
RET_A	B	(30,90]	0.0868	0.44	0.143	0.0838	0.104	
RET_A	C	(0,14]	0.0521	0.421	0.234	0.0679	0.0894	
RET_A	C	(14,30]	0.415	0.445	0.274	0.317	0.382	
RET_A	C	(30,90]	0.0024	0.63	0.00293	0.409	0.45	**
RET_A	D	(0,14]	0.000126	0.399	0.000659	0.541	0.659	***
RET_A	D	(14,30]	0.00534	0.626	0.00153	0.0879	0.1	*
RET_A	D	(30,90]	0.0022	0.305	0.00176	0.272	0.368	**
RET_A	E	(0,14]	0.0157	0.555	0.0251	0.422	0.476	*
RET_A	E	(14,30]	0.26	0.693	0.264	0.121	0.133	
RET_A	E	(30,90]	1.89E-13	0.742	3.16E-13	0.0395	0.0312	***
RET_A	E	(90,180]	0.0151	0.63	0.00721	0.0824	0.0928	*
IRF	A	(0,14]	0.15	0.00221	0.145	0.0184	0.0615	
IRF	A	(14,30]	0.776	0.00832	0.729	0.121	0.259	
IRF	A	(30,90]	0.558	0.00291	0.506	0.0691	0.18	
IRF	B	(0,14]	0.0191	0.00555	0.0175	0.311	0.57	*
IRF	B	(14,30]	0.494	0.00491	0.611	0.137	0.296	
IRF	B	(30,90]	0.0994	0.00479	0.156	0.0579	0.145	
IRF	C	(0,14]	0.775	0.00111	0.675	0.016	0.0779	
IRF	C	(14,30]	0.635	0.00233	0.294	0.0399	0.132	
IRF	C	(30,90]	0.000976	0.011	0.00121	0.0808	0.185	**
IRF	D	(0,14]	0.57	0.00684	0.191	0.0118	0.0474	
IRF	D	(14,30]	0.0717	0.00557	0.0121	0.0211	0.075	
IRF	D	(30,90]	0.102	0.00928	0.0515	0.00506	0.0214	
IRF	E	(0,14]	0.0984	0.0251	0.0465	0.0302	0.0691	
IRF	E	(14,30]	3.89E-09	0.0553	5.48E-10	0.000409	0.00132	***
IRF	E	(30,90]	5.61E-19	0.204	4.06E-20	2.22E-05	4.43E-05	***
IRF	E	(90,180]	0.000336	0.012	6.15E-05	0.00592	0.0182	***
Erythropoietin	A	(0,14]	0.572	0.204	0.632	0.598	0.451	
Erythropoietin	A	(14,30]	0.848	0.411	0.948	0.303	0.236	
Erythropoietin	A	(30,90]	0.77	0.26	0.744	0.509	0.388	
Erythropoietin	A	(90,180]	0.341	0.184	0.326	0.312	0.212	
Erythropoietin	B	(0,14]	0.0899	0.202	0.128	0.328	0.23	
Erythropoietin	B	(14,30]	0.419	0.292	0.533	0.289	0.214	
Erythropoietin	B	(30,90]	0.81	0.212	0.726	0.261	0.18	
Erythropoietin	B	(90,180]	0.0491	0.214	0.0785	0.286	0.197	*
Erythropoietin	B	(180,270]	0.94	0.203	0.849	0.55	0.412	
Erythropoietin	C	(0,14]	0.757	0.00965	0.606	0.145	0.0416	
Erythropoietin	C	(14,30]	0.448	0.184	0.43	0.673	0.513	
Erythropoietin	C	(30,90]	0.0422	0.0507	0.0475	0.142	0.0625	*
Erythropoietin	C	(90,180]	0.249	0.416	0.253	0.888	0.977	
Erythropoietin	C	(180,270]	0.62	0.175	0.654	0.535	0.386	
Erythropoietin	C	(270,360]	0.201	0.355	0.206	0.983	0.855	
Erythropoietin	D	(0,14]	0.0239	0.269	0.027	0.542	0.417	*
Erythropoietin	D	(14,30]	0.977	0.0912	0.887	0.245	0.129	
Erythropoietin	D	(30,90]	0.00484	0.0625	0.00538	0.173	0.0795	**
Erythropoietin	D	(90,180]	0.133	0.182	0.142	0.934	0.833	
Erythropoietin	D	(180,270]	0.915	0.275	0.847	0.662	0.53	
Erythropoietin	D	(270,360]	0.115	0.231	0.295	0.644	0.493	
Erythropoietin	E	(0,14]	0.0109	0.582	0.0131	0.796	0.871	*
Erythropoietin	E	(14,30]	1.96E-08	0.676	2.52E-08	0.66	0.711	***
Erythropoietin	E	(30,90]	2.74E-07	0.78	2.17E-07	0.28	0.249	***
Erythropoietin	E	(90,180]	0.000366	0.743	0.000417	0.234	0.204	***
Erythropoietin	E	(180,270]	0.0178	0.771	0.0172	0.581	0.61	*
Erythropoietin	E	(270,360]	0.787	0.132	0.983	0.535	0.375	

Table SM4: COVID-19 severity group associations with immune cell counts under two models for age correction

Model 1: log2(measure) ~ severity group + age + sex **Model 2:** log2(measure) ~ severity group + age + age^2 + sex
Severity group effects are relative to healthy controls, tested independently for each severity (A-E) and time window

p.lm1.sev = p-value for Mod1 severity effect, **p.lm1.age** = p-value for Mod1 age effect, **p.lm2.sev** = p-value for Mod2 severity effect, **p.lm2.age** = p-value for Mod2 age effect, **p.lm2.age** = p-value for Mod2 age^2 effect. Stars are based on significance thresholding of p.lm1.sev as shown in primary figures. *p<0.05, **p<0.005, ***p<0.0005

Measure (absolute counts)	severity_group	bin_custom	p.lm1.sev	p.lm1.age	p.lm2.sev	p.lm2.age	p.lm2.age2	stars
abs_CD3 +	A	(0,14]	0.644	0.513	0.72	0.407	0.344	
abs_CD3 +	A	(14,30]	0.439	0.143	0.413	0.652	0.483	
abs_CD3 +	A	(30,90]	0.988	0.115	1	0.786	0.591	
abs_CD3 +	A	(90,180]	0.387	0.415	0.387	0.914	0.809	
abs_CD3 +	B	(0,14]	0.338	0.31	0.282	0.622	0.503	
abs_CD3 +	B	(14,30]	0.673	0.223	0.502	0.303	0.214	
abs_CD3 +	B	(30,90]	0.44	0.0604	0.457	0.732	0.972	
abs_CD3 +	B	(90,180]	0.185	0.0439	0.147	0.656	0.425	
abs_CD3 +	B	(180,360]	0.848	0.26	0.87	0.551	0.431	
abs_CD3 +	C	(0,14]	0.00851	0.679	0.00895	0.788	0.85	*
abs_CD3 +	C	(14,30]	0.873	0.347	0.997	0.459	0.355	
abs_CD3 +	C	(30,90]	0.909	0.0726	0.974	0.261	0.144	
abs_CD3 +	C	(90,180]	0.735	0.041	0.723	0.37	0.178	
abs_CD3 +	C	(180,360]	0.0707	0.159	0.0867	0.599	0.437	
abs_CD3 +	D	(0,14]	4.73E-05	0.445	5.02E-05	0.491	0.592	***
abs_CD3 +	D	(14,30]	0.975	0.0547	0.945	0.796	0.481	
abs_CD3 +	D	(30,90]	0.552	0.0771	0.586	0.653	0.428	
abs_CD3 +	D	(90,180]	0.551	0.0738	0.556	0.77	0.924	
abs_CD3 +	D	(180,360]	0.0596	0.0759	0.0565	0.323	0.191	
abs_CD3 +	E	(0,14]	5.47E-06	0.137	1.54E-05	0.984	0.699	***
abs_CD3 +	E	(14,30]	0.00247	0.196	0.00221	0.687	0.483	**
abs_CD3 +	E	(30,90]	0.019	0.563	0.0262	0.454	0.512	*
abs_CD3 +	E	(90,180]	0.295	0.225	0.283	0.447	0.579	
abs_CD3 +	E	(180,360]	0.293	0.823	0.272	0.413	0.428	
abs_CD4+	A	(0,14]	0.699	0.986	0.775	0.36	0.355	
abs_CD4+	A	(14,30]	0.561	0.391	0.539	0.7	0.595	
abs_CD4+	A	(30,90]	0.752	0.396	0.74	0.597	0.499	
abs_CD4+	A	(90,180]	0.786	0.808	0.784	0.941	0.909	
abs_CD4+	B	(0,14]	0.292	0.849	0.227	0.422	0.398	
abs_CD4+	B	(14,30]	0.462	0.6	0.324	0.24	0.202	
abs_CD4+	B	(30,90]	0.405	0.171	0.407	0.903	0.918	
abs_CD4+	B	(90,180]	0.121	0.273	0.0893	0.462	0.352	
abs_CD4+	B	(180,360]	0.481	0.572	0.502	0.432	0.375	
abs_CD4+	C	(0,14]	0.00486	0.768	0.00486	0.736	0.685	**
abs_CD4+	C	(14,30]	0.528	0.774	0.599	0.62	0.578	
abs_CD4+	C	(30,90]	0.729	0.328	0.683	0.362	0.272	
abs_CD4+	C	(90,180]	0.828	0.191	0.836	0.365	0.231	
abs_CD4+	C	(180,360]	0.226	0.476	0.257	0.643	0.554	
abs_CD4+	D	(0,14]	3.57E-05	0.531	4.37E-05	0.924	0.968	***
abs_CD4+	D	(14,30]	0.259	0.0783	0.36	0.237	0.107	
abs_CD4+	D	(30,90]	0.948	0.193	0.993	0.486	0.341	
abs_CD4+	D	(90,180]	0.961	0.431	0.956	0.527	0.634	
abs_CD4+	D	(180,360]	0.054	0.198	0.051	0.28	0.188	
abs_CD4+	E	(0,14]	3.67E-06	0.0629	1.64E-05	0.152	0.0533	***
abs_CD4+	E	(14,30]	0.00969	0.17	0.00549	0.176	0.091	*
abs_CD4+	E	(30,90]	0.039	0.5	0.0407	0.997	0.898	*
abs_CD4+	E	(90,180]	0.826	0.39	0.822	0.787	0.905	
abs_CD4+	E	(180,360]	0.167	0.994	0.158	0.544	0.537	
CD4+/Naive	A	(0,14]	0.496	0.151	0.483	0.578	0.742	
CD4+/Naive	A	(14,30]	0.893	0.0611	0.862	0.361	0.538	
CD4+/Naive	A	(30,90]	0.323	0.0987	0.333	0.661	0.865	
CD4+/Naive	A	(90,180]	0.206	0.197	0.19	0.247	0.335	
CD4+/Naive	B	(0,14]	0.312	0.11	0.328	0.808	0.982	
CD4+/Naive	B	(14,30]	0.301	0.0495	0.301	0.865	0.879	
CD4+/Naive	B	(30,90]	0.393	0.0309	0.474	0.362	0.572	
CD4+/Naive	B	(90,180]	0.0647	0.0121	0.0824	0.496	0.797	
CD4+/Naive	B	(180,360]	0.849	0.181	0.849	0.905	0.922	
CD4+/Naive	C	(0,14]	0.00925	0.718	0.00804	0.35	0.379	*
CD4+/Naive	C	(14,30]	0.188	0.213	0.17	0.447	0.586	
CD4+/Naive	C	(30,90]	0.256	0.0686	0.247	0.849	0.602	
CD4+/Naive	C	(90,180]	0.538	0.0448	0.541	0.841	0.529	
CD4+/Naive	C	(180,360]	0.973	0.107	0.98	0.728	0.941	
CD4+/Naive	D	(0,14]	0.00225	0.0318	0.00264	0.946	0.578	**
CD4+/Naive	D	(14,30]	0.385	0.00837	0.516	0.284	0.0862	
CD4+/Naive	D	(30,90]	0.709	0.0634	0.718	0.663	0.928	
CD4+/Naive	D	(90,180]	0.777	0.192	0.794	0.128	0.202	
CD4+/Naive	D	(180,360]	0.0795	0.0523	0.0839	0.87	0.615	

CD4+/Naive	E	(0,14]	0.00202	0.00891	0.0106	0.266	0.0707	**
CD4+/Naive	E	(14,30]	0.0898	0.00683	0.0501	0.121	0.0272	
CD4+/Naive	E	(30,90]	0.145	0.0342	0.121	0.637	0.38	
CD4+/Naive	E	(90,180]	0.0927	0.139	0.0978	0.738	0.941	
CD4+/Naive	E	(180,360]	0.056	0.131	0.0655	0.898	0.693	
CD4+/CM	A	(0,14]	0.184	0.145	0.166	0.342	0.467	
CD4+/CM	A	(14,30]	0.0297	0.354	0.0301	0.603	0.708	*
CD4+/CM	A	(30,90]	0.0868	0.37	0.0939	0.513	0.605	
CD4+/CM	A	(90,180]	0.394	0.238	0.407	0.964	0.812	
CD4+/CM	B	(0,14]	0.197	0.0921	0.318	0.169	0.266	
CD4+/CM	B	(14,30]	0.11	0.227	0.175	0.254	0.337	
CD4+/CM	B	(30,90]	0.27	0.676	0.327	0.603	0.646	
CD4+/CM	B	(90,180]	0.79	0.213	0.939	0.294	0.392	
CD4+/CM	B	(180,360]	0.249	0.364	0.26	0.516	0.61	
CD4+/CM	C	(0,14]	0.225	0.225	0.22	0.936	0.737	
CD4+/CM	C	(14,30]	0.683	0.405	0.595	0.345	0.42	
CD4+/CM	C	(30,90]	0.659	0.688	0.712	0.243	0.265	
CD4+/CM	C	(90,180]	0.979	0.97	0.981	0.413	0.407	
CD4+/CM	C	(180,360]	0.548	0.471	0.596	0.52	0.596	
CD4+/CM	D	(0,14]	0.00598	0.231	0.00634	0.984	0.806	*
CD4+/CM	D	(14,30]	0.724	0.559	0.809	0.375	0.436	
CD4+/CM	D	(30,90]	0.27	0.522	0.298	0.373	0.431	
CD4+/CM	D	(90,180]	0.465	0.17	0.474	0.925	0.691	
CD4+/CM	D	(180,360]	0.252	0.594	0.258	0.411	0.456	
CD4+/CM	E	(0,14]	0.0241	0.846	0.0896	0.0902	0.0737	*
CD4+/CM	E	(14,30]	0.716	0.897	0.626	0.195	0.194	
CD4+/CM	E	(30,90]	0.748	0.325	0.733	0.987	0.842	
CD4+/CM	E	(90,180]	0.0676	0.546	0.0704	0.985	0.929	
CD4+/CM	E	(180,360]	0.872	0.509	0.806	0.357	0.412	
CD4+/EM	A	(0,14]	0.646	0.724	0.683	0.621	0.657	
CD4+/EM	A	(14,30]	0.887	0.774	0.873	0.794	0.755	
CD4+/EM	A	(30,90]	0.863	0.544	0.862	0.836	0.913	
CD4+/EM	A	(90,180]	0.104	0.692	0.109	0.959	0.99	
CD4+/EM	B	(0,14]	0.354	0.964	0.365	0.965	0.959	
CD4+/EM	B	(14,30]	0.71	0.795	0.688	0.884	0.849	
CD4+/EM	B	(30,90]	0.327	0.362	0.359	0.756	0.868	
CD4+/EM	B	(90,180]	0.982	0.921	0.917	0.716	0.7	
CD4+/EM	B	(180,360]	0.992	0.669	0.993	0.952	0.994	
CD4+/EM	C	(0,14]	0.0252	0.684	0.0279	0.925	0.857	*
CD4+/EM	C	(14,30]	0.724	0.857	0.751	0.893	0.866	
CD4+/EM	C	(30,90]	0.529	0.468	0.542	0.955	0.852	
CD4+/EM	C	(90,180]	0.371	0.334	0.375	0.888	0.729	
CD4+/EM	C	(180,360]	0.146	0.688	0.156	0.98	0.965	
CD4+/EM	D	(0,14]	0.00128	0.805	0.0011	0.312	0.276	**
CD4+/EM	D	(14,30]	0.13	0.528	0.137	0.84	0.948	
CD4+/EM	D	(30,90]	0.634	0.467	0.629	0.946	0.837	
CD4+/EM	D	(90,180]	0.825	0.634	0.829	0.851	0.93	
CD4+/EM	D	(180,360]	0.594	0.475	0.601	0.918	0.822	
CD4+/EM	E	(0,14]	0.000854	0.441	0.00223	0.844	0.697	**
CD4+/EM	E	(14,30]	0.00198	0.906	0.00262	0.554	0.527	**
CD4+/EM	E	(30,90]	0.000324	0.628	0.000595	0.152	0.122	***
CD4+/EM	E	(90,180]	0.893	0.609	0.94	0.259	0.291	
CD4+/EM	E	(180,360]	0.179	0.516	0.186	0.94	0.971	
CD4+/EMRA	A	(0,14]	0.104	0.413	0.134	0.154	0.115	
CD4+/EMRA	A	(14,30]	0.382	0.227	0.438	0.105	0.0656	
CD4+/EMRA	A	(30,90]	0.0138	0.0918	0.0125	0.436	0.285	*
CD4+/EMRA	A	(90,180]	0.909	0.243	0.836	0.122	0.0794	
CD4+/EMRA	B	(0,14]	0.0776	0.25	0.0462	0.322	0.231	
CD4+/EMRA	B	(14,30]	0.116	0.43	0.0595	0.16	0.121	
CD4+/EMRA	B	(30,90]	0.0535	0.0855	0.036	0.446	0.291	
CD4+/EMRA	B	(90,180]	0.141	0.171	0.0881	0.316	0.212	
CD4+/EMRA	B	(180,360]	0.732	0.159	0.755	0.344	0.235	
CD4+/EMRA	C	(0,14]	0.000809	0.554	0.000955	0.978	0.924	**
CD4+/EMRA	C	(14,30]	0.272	0.243	0.326	0.722	0.563	
CD4+/EMRA	C	(30,90]	0.76	0.331	0.78	0.871	0.736	
CD4+/EMRA	C	(90,180]	0.817	0.407	0.818	0.934	0.797	
CD4+/EMRA	C	(180,360]	0.185	0.322	0.202	0.962	0.827	
CD4+/EMRA	D	(0,14]	0.0386	0.941	0.039	0.712	0.718	*
CD4+/EMRA	D	(14,30]	0.956	0.274	0.972	0.38	0.512	
CD4+/EMRA	D	(30,90]	0.901	0.204	0.876	0.838	0.654	
CD4+/EMRA	D	(90,180]	0.258	0.0834	0.266	0.619	0.372	
CD4+/EMRA	D	(180,360]	0.0712	0.193	0.075	0.72	0.557	
CD4+/EMRA	E	(0,14]	0.00352	0.16	0.00632	0.799	0.936	**
CD4+/EMRA	E	(14,30]	0.0034	0.489	0.00397	0.816	0.932	**
CD4+/EMRA	E	(30,90]	0.000778	0.895	0.00123	0.368	0.373	**
CD4+/EMRA	E	(90,180]	0.478	0.341	0.493	0.66	0.782	
CD4+/EMRA	E	(180,360]	0.163	0.369	0.173	0.819	0.939	

CD4+/NonNaive/HLADR+CD38+	A	(0,14]	0.41	0.0692	0.434	0.915	0.853	
CD4+/NonNaive/HLADR+CD38+	A	(14,30]	0.058	0.112	0.0687	0.788	0.595	
CD4+/NonNaive/HLADR+CD38+	A	(30,90]	0.408	0.213	0.409	0.949	0.794	
CD4+/NonNaive/HLADR+CD38+	A	(90,180]	0.222	0.281	0.245	0.685	0.561	
CD4+/NonNaive/HLADR+CD38+	B	(0,14]	0.0102	0.0534	0.0123	0.824	0.936	*
CD4+/NonNaive/HLADR+CD38+	B	(14,30]	0.00659	0.0327	0.00816	0.77	0.973	*
CD4+/NonNaive/HLADR+CD38+	B	(30,90]	0.281	0.325	0.238	0.592	0.488	
CD4+/NonNaive/HLADR+CD38+	B	(90,180]	0.21	0.115	0.209	0.651	0.838	
CD4+/NonNaive/HLADR+CD38+	C	(0,14]	0.517	0.202	0.539	0.653	0.848	
CD4+/NonNaive/HLADR+CD38+	C	(14,30]	0.684	0.127	0.752	0.959	0.734	
CD4+/NonNaive/HLADR+CD38+	C	(30,90]	0.342	0.489	0.345	0.466	0.536	
CD4+/NonNaive/HLADR+CD38+	C	(90,180]	0.761	0.292	0.768	0.757	0.924	
CD4+/NonNaive/HLADR+CD38+	C	(180,360]	0.43	0.258	0.437	0.858	0.992	
CD4+/NonNaive/HLADR+CD38+	D	(0,14]	0.247	0.287	0.26	0.817	0.988	
CD4+/NonNaive/HLADR+CD38+	D	(14,30]	0.0788	0.485	0.0637	0.402	0.484	
CD4+/NonNaive/HLADR+CD38+	D	(30,90]	0.0944	0.245	0.0988	0.724	0.892	
CD4+/NonNaive/HLADR+CD38+	D	(90,180]	0.656	0.168	0.677	0.534	0.341	
CD4+/NonNaive/HLADR+CD38+	D	(180,360]	0.27	0.295	0.286	0.978	0.839	
CD4+/NonNaive/HLADR+CD38+	E	(0,14]	0.372	0.93	0.673	0.205	0.183	
CD4+/NonNaive/HLADR+CD38+	E	(14,30]	0.109	0.695	0.118	0.22	0.242	
CD4+/NonNaive/HLADR+CD38+	E	(30,90]	0.0693	0.338	0.0607	0.511	0.396	
CD4+/NonNaive/HLADR+CD38+	E	(90,180]	0.305	0.229	0.301	0.289	0.195	
CD4+/NonNaive/HLADR+CD38+	E	(180,360]	0.303	0.238	0.321	0.899	0.939	
CD4+/Tfh-like	A	(0,14]	0.081	0.612	0.0711	0.473	0.418	
CD4+/Tfh-like	A	(14,30]	0.872	0.303	0.855	0.841	0.71	
CD4+/Tfh-like	A	(30,90]	0.0672	0.377	0.0733	0.674	0.567	
CD4+/Tfh-like	A	(90,180]	0.452	0.749	0.466	0.863	0.821	
CD4+/Tfh-like	B	(0,14]	0.00109	0.667	0.00315	0.239	0.262	**
CD4+/Tfh-like	B	(14,30]	0.665	0.675	0.671	0.925	0.978	
CD4+/Tfh-like	B	(30,90]	0.253	0.577	0.285	0.909	0.836	
CD4+/Tfh-like	B	(90,180]	0.167	0.359	0.132	0.545	0.443	
CD4+/Tfh-like	B	(180,360]	0.82	0.784	0.836	0.517	0.483	
CD4+/Tfh-like	C	(0,14]	0.0771	0.555	0.0759	0.657	0.746	
CD4+/Tfh-like	C	(14,30]	0.121	0.706	0.161	0.688	0.632	
CD4+/Tfh-like	C	(30,90]	0.971	0.0901	0.934	0.139	0.0714	
CD4+/Tfh-like	C	(90,180]	0.696	0.271	0.687	0.156	0.0938	
CD4+/Tfh-like	C	(180,360]	0.805	0.443	0.727	0.443	0.363	
CD4+/Tfh-like	D	(0,14]	0.000245	0.175	0.000295	0.18	0.0929	***
CD4+/Tfh-like	D	(14,30]	0.126	0.101	0.192	0.0798	0.0306	
CD4+/Tfh-like	D	(30,90]	0.056	0.201	0.0444	0.293	0.19	
CD4+/Tfh-like	D	(90,180]	0.72	0.172	0.728	0.899	0.668	
CD4+/Tfh-like	D	(180,360]	0.807	0.302	0.814	0.238	0.171	
CD4+/Tfh-like	E	(0,14]	0.000292	0.444	0.000784	0.732	0.597	***
CD4+/Tfh-like	E	(14,30]	1.49E-05	0.219	9.23E-06	0.29	0.175	***
CD4+/Tfh-like	E	(30,90]	0.00117	0.359	0.0012	0.86	0.727	**
CD4+/Tfh-like	E	(90,180]	0.997	0.122	0.964	0.573	0.395	
CD4+/Tfh-like	E	(180,360]	0.199	0.339	0.229	0.635	0.517	
CD25pCD127lo	A	(0,14]	0.44	0.0654	0.483	0.913	0.683	
CD25pCD127lo	A	(14,30]	0.304	0.42	0.369	0.158	0.193	
CD25pCD127lo	A	(30,90]	0.675	0.224	0.653	0.256	0.337	
CD25pCD127lo	A	(90,180]	0.223	0.39	0.181	0.137	0.17	
CD25pCD127lo	B	(0,14]	0.393	0.968	0.415	0.948	0.952	
CD25pCD127lo	B	(14,30]	0.932	0.178	0.908	0.709	0.868	
CD25pCD127lo	B	(30,90]	0.705	0.0371	0.825	0.197	0.328	
CD25pCD127lo	B	(90,180]	0.0132	0.281	0.0161	0.846	0.98	*
CD25pCD127lo	B	(180,360]	0.272	0.29	0.26	0.415	0.507	
CD25pCD127lo	C	(0,14]	0.00313	0.979	0.00271	0.451	0.445	**
CD25pCD127lo	C	(14,30]	0.0478	0.806	0.0192	0.0829	0.0857	*
CD25pCD127lo	C	(30,90]	0.535	0.066	0.542	0.487	0.708	
CD25pCD127lo	C	(90,180]	0.448	0.433	0.438	0.407	0.492	
CD25pCD127lo	C	(180,360]	0.995	0.196	0.987	0.289	0.392	
CD25pCD127lo	D	(0,14]	0.00353	0.0166	0.00427	0.808	0.753	**
CD25pCD127lo	D	(14,30]	0.362	0.0503	0.381	0.687	0.958	
CD25pCD127lo	D	(30,90]	0.848	0.155	0.854	0.311	0.448	
CD25pCD127lo	D	(90,180]	0.636	0.262	0.65	0.0337	0.0539	
CD25pCD127lo	D	(180,360]	0.118	0.0977	0.132	0.525	0.715	
CD25pCD127lo	E	(0,14]	8.03E-09	0.0909	2.23E-08	0.519	0.821	***
CD25pCD127lo	E	(14,30]	0.022	0.0723	0.0227	0.854	0.818	*
CD25pCD127lo	E	(30,90]	0.00078	0.734	0.00114	0.382	0.408	**
CD25pCD127lo	E	(90,180]	0.636	0.0339	0.644	0.257	0.447	
CD25pCD127lo	E	(180,360]	0.0508	0.885	0.0192	0.0464	0.0459	
abs_CD8+	A	(0,14]	0.589	0.0975	0.708	0.188	0.108	
abs_CD8+	A	(14,30]	0.506	0.0205	0.435	0.286	0.141	
abs_CD8+	A	(30,90]	0.601	0.0157	0.623	0.404	0.209	
abs_CD8+	A	(90,180]	0.232	0.0666	0.213	0.491	0.318	
abs_CD8+	B	(0,14]	0.542	0.0282	0.378	0.37	0.199	
abs_CD8+	B	(14,30]	0.739	0.0194	0.435	0.085	0.033	

abs_CD8+	B	(30,90]	0.584	0.00725	0.494	0.748	0.435	
abs_CD8+	B	(90,180]	0.894	0.00229	0.655	0.311	0.118	
abs_CD8+	B	(180,360]	0.982	0.0704	0.976	0.218	0.123	
abs_CD8+	C	(0,14]	0.0355	0.158	0.0384	0.828	0.942	*
abs_CD8+	C	(14,30]	0.78	0.0565	0.985	0.156	0.0726	
abs_CD8+	C	(30,90]	0.665	0.00749	0.749	0.0638	0.018	
abs_CD8+	C	(90,180]	0.442	0.00492	0.435	0.634	0.275	
abs_CD8+	C	(180,360]	0.13	0.021	0.172	0.176	0.0755	
abs_CD8+	D	(0,14]	0.00132	0.273	0.00122	0.288	0.398	**
abs_CD8+	D	(14,30]	0.436	0.0682	0.444	0.705	0.978	
abs_CD8+	D	(30,90]	0.425	0.0226	0.461	0.579	0.315	
abs_CD8+	D	(90,180]	0.365	0.0029	0.372	0.486	0.164	
abs_CD8+	D	(180,360]	0.234	0.0201	0.211	0.118	0.0468	
abs_CD8+	E	(0,14]	9.38E-05	0.224	0.000102	0.379	0.542	***
abs_CD8+	E	(14,30]	0.000682	0.228	0.000957	0.373	0.519	**
abs_CD8+	E	(30,90]	0.0663	0.162	0.0865	0.351	0.491	
abs_CD8+	E	(90,180]	0.274	0.0762	0.281	0.773	0.976	
abs_CD8+	E	(180,360]	0.383	0.342	0.385	0.767	0.894	
CD8+/Naive	A	(0,14]	0.917	3.55E-05	0.877	0.833	0.596	
CD8+/Naive	A	(14,30]	0.437	1.67E-05	0.434	0.579	0.819	
CD8+/Naive	A	(30,90]	0.339	4.13E-05	0.35	0.586	0.837	
CD8+/Naive	A	(90,180]	0.577	0.000165	0.586	0.462	0.937	
CD8+/Naive	B	(0,14]	0.384	4.64E-06	0.495	0.878	0.494	
CD8+/Naive	B	(14,30]	0.38	2.38E-07	0.552	0.798	0.225	
CD8+/Naive	B	(30,90]	0.401	3.98E-06	0.456	0.63	0.728	
CD8+/Naive	B	(90,180]	0.855	1.41E-06	0.81	0.547	0.766	
CD8+/Naive	B	(180,360]	0.98	0.000205	0.99	0.84	0.638	
CD8+/Naive	C	(0,14]	0.554	9.50E-06	0.591	0.723	0.515	
CD8+/Naive	C	(14,30]	0.715	5.66E-05	0.641	0.873	0.507	
CD8+/Naive	C	(30,90]	0.393	8.70E-06	0.441	0.597	0.154	
CD8+/Naive	C	(90,180]	0.308	1.14E-08	0.297	0.761	0.0852	
CD8+/Naive	C	(180,360]	0.597	5.59E-05	0.668	0.964	0.414	
CD8+/Naive	D	(0,14]	0.0516	6.95E-06	0.0556	0.403	0.805	
CD8+/Naive	D	(14,30]	0.838	2.25E-05	0.791	0.561	0.629	
CD8+/Naive	D	(30,90]	0.551	2.95E-05	0.588	0.86	0.486	
CD8+/Naive	D	(90,180]	0.599	5.17E-07	0.588	0.712	0.373	
CD8+/Naive	D	(180,360]	0.379	9.53E-05	0.384	0.811	0.323	
CD8+/Naive	E	(0,14]	0.0423	0.00242	0.0182	0.0355	0.158	*
CD8+/Naive	E	(14,30]	0.265	1.24E-06	0.249	0.516	0.58	
CD8+/Naive	E	(30,90]	0.805	4.62E-08	0.772	0.449	0.714	
CD8+/Naive	E	(90,180]	0.731	4.70E-05	0.714	0.214	0.636	
CD8+/Naive	E	(180,360]	0.248	9.27E-06	0.302	0.75	0.23	
CD8+/CM	A	(0,14]	0.00484	0.131	0.00299	0.0876	0.137	**
CD8+/CM	A	(14,30]	5.47E-05	0.274	5.74E-05	0.433	0.538	***
CD8+/CM	A	(30,90]	0.000325	0.477	0.000368	0.229	0.27	***
CD8+/CM	A	(90,180]	0.00875	0.212	0.00969	0.777	0.933	*
CD8+/CM	B	(0,14]	0.0108	0.443	0.0248	0.228	0.273	*
CD8+/CM	B	(14,30]	0.0021	0.614	0.00552	0.196	0.219	**
CD8+/CM	B	(30,90]	0.00801	0.923	0.0161	0.3	0.301	*
CD8+/CM	B	(90,180]	0.469	0.961	0.613	0.361	0.35	
CD8+/CM	B	(180,360]	0.447	0.412	0.463	0.227	0.275	
CD8+/CM	C	(0,14]	0.491	0.322	0.533	0.577	0.437	
CD8+/CM	C	(14,30]	0.0446	0.545	0.0277	0.157	0.184	*
CD8+/CM	C	(30,90]	0.00518	0.792	0.00596	0.0797	0.083	*
CD8+/CM	C	(90,180]	0.878	0.701	0.877	0.497	0.539	
CD8+/CM	C	(180,360]	0.387	0.879	0.481	0.144	0.145	
CD8+/CM	D	(0,14]	0.0672	0.0828	0.0655	0.85	0.559	
CD8+/CM	D	(14,30]	0.174	0.699	0.163	0.579	0.629	
CD8+/CM	D	(30,90]	0.00844	0.942	0.01	0.0862	0.0833	*
CD8+/CM	D	(90,180]	0.585	0.802	0.567	0.208	0.217	
CD8+/CM	D	(180,360]	0.52	0.681	0.525	0.266	0.29	
CD8+/CM	E	(0,14]	0.154	0.748	0.235	0.557	0.598	
CD8+/CM	E	(14,30]	0.82	0.342	0.82	0.811	0.973	
CD8+/CM	E	(30,90]	0.0891	0.106	0.0702	0.482	0.309	
CD8+/CM	E	(90,180]	0.359	0.728	0.357	0.83	0.779	
CD8+/CM	E	(180,360]	0.161	0.391	0.192	0.322	0.391	
CD8+/EM	A	(0,14]	0.861	0.23	0.815	0.422	0.536	
CD8+/EM	A	(14,30]	0.341	0.389	0.331	0.533	0.624	
CD8+/EM	A	(30,90]	0.533	0.513	0.543	0.78	0.86	
CD8+/EM	A	(90,180]	0.123	0.48	0.129	0.901	0.991	
CD8+/EM	B	(0,14]	0.672	0.449	0.657	0.781	0.876	
CD8+/EM	B	(14,30]	0.734	0.372	0.721	0.788	0.899	
CD8+/EM	B	(30,90]	0.419	0.784	0.442	0.977	0.941	
CD8+/EM	B	(90,180]	0.933	0.942	0.931	0.993	0.983	
CD8+/EM	B	(180,360]	0.593	0.486	0.601	0.821	0.907	
CD8+/EM	C	(0,14]	0.0295	0.496	0.0297	0.87	0.757	*
CD8+/EM	C	(14,30]	0.718	0.562	0.812	0.422	0.477	

CD8+/EM	C	(30,90]	0.594	0.888	0.638	0.37	0.35	
CD8+/EM	C	(90,180]	0.887	0.537	0.888	0.871	0.97	
CD8+/EM	C	(180,360]	0.197	0.857	0.229	0.523	0.537	
CD8+/EM	D	(0,14]	0.00267	0.182	0.00227	0.42	0.261	**
CD8+/EM	D	(14,30]	0.803	0.7	0.775	0.699	0.756	
CD8+/EM	D	(30,90]	0.569	0.871	0.598	0.6	0.615	
CD8+/EM	D	(90,180]	0.803	0.518	0.803	0.776	0.88	
CD8+/EM	D	(180,360]	0.621	0.875	0.628	0.562	0.574	
CD8+/EM	E	(0,14]	4.87E-05	0.727	0.000116	0.964	0.97	***
CD8+/EM	E	(14,30]	0.000572	0.153	0.000801	0.639	0.423	**
CD8+/EM	E	(30,90]	0.0431	0.135	0.0611	0.382	0.243	*
CD8+/EM	E	(90,180]	0.774	0.737	0.751	0.604	0.557	
CD8+/EM	E	(180,360]	0.596	0.821	0.559	0.535	0.555	
CD8+/EMRA	A	(0,14]	0.0905	0.584	0.118	0.143	0.116	
CD8+/EMRA	A	(14,30]	0.301	0.418	0.348	0.0664	0.0466	
CD8+/EMRA	A	(30,90]	0.0584	0.254	0.0475	0.18	0.122	
CD8+/EMRA	A	(90,180]	0.93	0.539	0.988	0.171	0.138	
CD8+/EMRA	B	(0,14]	0.384	0.693	0.176	0.05	0.0402	
CD8+/EMRA	B	(14,30]	0.661	0.933	0.35	0.0283	0.0272	
CD8+/EMRA	B	(30,90]	0.333	0.454	0.2	0.151	0.115	
CD8+/EMRA	B	(90,180]	0.531	0.591	0.903	0.025	0.0183	
CD8+/EMRA	B	(180,360]	0.689	0.482	0.643	0.108	0.0817	
CD8+/EMRA	C	(0,14]	0.0502	0.708	0.0563	0.768	0.704	
CD8+/EMRA	C	(14,30]	0.922	0.448	0.736	0.195	0.145	
CD8+/EMRA	C	(30,90]	0.636	0.178	0.719	0.0965	0.0543	
CD8+/EMRA	C	(90,180]	0.602	0.337	0.6	0.416	0.302	
CD8+/EMRA	C	(180,360]	0.114	0.23	0.155	0.178	0.116	
CD8+/EMRA	D	(0,14]	0.326	0.628	0.324	0.667	0.739	
CD8+/EMRA	D	(14,30]	0.245	0.426	0.271	0.659	0.784	
CD8+/EMRA	D	(30,90]	0.244	0.277	0.268	0.66	0.514	
CD8+/EMRA	D	(90,180]	0.0607	0.109	0.0629	0.454	0.263	
CD8+/EMRA	D	(180,360]	0.0909	0.171	0.0819	0.104	0.0601	
CD8+/EMRA	E	(0,14]	0.0144	0.723	0.011	0.388	0.422	*
CD8+/EMRA	E	(14,30]	0.0172	0.716	0.0223	0.387	0.334	*
CD8+/EMRA	E	(30,90]	0.0759	0.992	0.0947	0.5	0.491	
CD8+/EMRA	E	(90,180]	0.0393	0.255	0.0444	0.663	0.518	*
CD8+/EMRA	E	(180,360]	0.296	0.385	0.275	0.666	0.556	
CD8+/NonNaive/HLADR+CD38+	A	(0,14]	0.24	0.294	0.21	0.368	0.455	
CD8+/NonNaive/HLADR+CD38+	A	(14,30]	0.0329	0.286	0.0267	0.236	0.303	*
CD8+/NonNaive/HLADR+CD38+	A	(30,90]	0.76	0.761	0.794	0.244	0.258	
CD8+/NonNaive/HLADR+CD38+	A	(90,180]	0.0186	0.522	0.0182	0.512	0.573	*
CD8+/NonNaive/HLADR+CD38+	B	(0,14]	0.000145	0.151	0.000445	0.175	0.25	***
CD8+/NonNaive/HLADR+CD38+	B	(14,30]	0.00691	0.193	0.0162	0.114	0.161	*
CD8+/NonNaive/HLADR+CD38+	B	(30,90]	0.931	0.812	0.893	0.231	0.239	
CD8+/NonNaive/HLADR+CD38+	B	(90,180]	0.703	0.942	0.951	0.12	0.118	
CD8+/NonNaive/HLADR+CD38+	C	(0,14]	0.215	0.718	0.191	0.444	0.48	
CD8+/NonNaive/HLADR+CD38+	C	(14,30]	0.21	0.681	0.158	0.341	0.372	
CD8+/NonNaive/HLADR+CD38+	C	(30,90]	0.0818	0.407	0.0687	0.0523	0.0345	
CD8+/NonNaive/HLADR+CD38+	C	(90,180]	0.921	0.731	0.868	0.294	0.317	
CD8+/NonNaive/HLADR+CD38+	C	(180,360]	0.0804	0.885	0.082	0.07	0.0628	
CD8+/NonNaive/HLADR+CD38+	D	(0,14]	0.756	0.519	0.71	0.606	0.705	
CD8+/NonNaive/HLADR+CD38+	D	(14,30]	2.52E-06	0.28	1.56E-07	0.025	0.0109	***
CD8+/NonNaive/HLADR+CD38+	D	(30,90]	0.000487	0.787	0.000527	0.39	0.41	***
CD8+/NonNaive/HLADR+CD38+	D	(90,180]	0.231	0.487	0.2	0.116	0.0777	
CD8+/NonNaive/HLADR+CD38+	D	(180,360]	0.9	0.828	0.783	0.0763	0.0671	
CD8+/NonNaive/HLADR+CD38+	E	(0,14]	0.735	0.866	0.754	0.963	0.995	
CD8+/NonNaive/HLADR+CD38+	E	(14,30]	0.0383	0.595	0.0421	0.466	0.531	*
CD8+/NonNaive/HLADR+CD38+	E	(30,90]	4.16E-06	0.461	5.09E-06	0.984	0.906	***
CD8+/NonNaive/HLADR+CD38+	E	(90,180]	0.0384	0.675	0.039	0.429	0.379	*
CD8+/NonNaive/HLADR+CD38+	E	(180,360]	0.0411	0.799	0.0332	0.386	0.403	*
gdTcells	A	(0,14]	0.351	0.000473	0.315	0.202	0.487	
gdTcells	A	(14,30]	0.279	0.000146	0.261	0.151	0.426	
gdTcells	A	(30,90]	0.656	0.0011	0.685	0.151	0.369	
gdTcells	A	(90,180]	0.575	0.000666	0.548	0.155	0.387	
gdTcells	B	(0,14]	0.353	2.08E-05	0.512	0.0493	0.215	
gdTcells	B	(14,30]	0.987	4.41E-05	0.8	0.0925	0.32	
gdTcells	B	(30,90]	0.739	0.000245	0.58	0.0853	0.262	
gdTcells	B	(90,180]	0.934	0.000211	0.986	0.262	0.63	
gdTcells	B	(180,360]	0.0275	0.00348	0.03	0.229	0.466	*
gdTcells	C	(0,14]	0.0228	0.000527	0.0184	0.0878	0.314	*
gdTcells	C	(14,30]	0.9	0.00125	0.905	0.526	0.983	
gdTcells	C	(30,90]	0.869	0.00016	0.869	0.505	0.968	
gdTcells	C	(90,180]	0.975	0.000812	0.988	0.0807	0.285	
gdTcells	C	(180,360]	0.443	0.00114	0.428	0.383	0.761	
gdTcells	D	(0,14]	0.00186	0.16	0.000859	0.00676	0.0135	**
gdTcells	D	(14,30]	0.305	0.0426	0.149	0.0129	0.0358	
gdTcells	D	(30,90]	0.651	0.00909	0.773	0.028	0.0802	

gdTcells	D	(90,180]	0.551	0.0623	0.508	0.0542	0.117	
gdTcells	D	(180,360]	0.766	0.00401	0.789	0.156	0.348	
gdTcells	E	(0,14]	0.000204	0.0271	2.29E-05	0.00449	0.0164	***
gdTcells	E	(14,30]	0.000827	0.173	0.00142	0.111	0.183	**
gdTcells	E	(30,90]	0.00674	0.151	0.0136	0.0325	0.056	*
gdTcells	E	(90,180]	0.837	0.00626	0.937	0.101	0.241	
gdTcells	E	(180,360]	0.141	0.00247	0.129	0.121	0.298	
gdTcells/TCR Vg9+ TCR-DV2high	A	(0,14]	0.639	0.000125	0.523	0.0493	0.182	
gdTcells/TCR Vg9+ TCR-DV2high	A	(14,30]	0.38	8.27E-05	0.329	0.0367	0.15	
gdTcells/TCR Vg9+ TCR-DV2high	A	(30,90]	0.989	0.000455	0.938	0.0427	0.145	
gdTcells/TCR Vg9+ TCR-DV2high	A	(90,180]	0.535	0.000158	0.487	0.0488	0.176	
gdTcells/TCR Vg9+ TCR-DV2high	B	(0,14]	0.147	4.83E-06	0.291	0.00649	0.0507	
gdTcells/TCR Vg9+ TCR-DV2high	B	(14,30]	0.884	5.67E-06	0.588	0.0158	0.0979	
gdTcells/TCR Vg9+ TCR-DV2high	B	(30,90]	0.491	2.95E-05	0.306	0.0183	0.0932	
gdTcells/TCR Vg9+ TCR-DV2high	B	(90,180]	0.829	4.96E-05	0.995	0.0905	0.319	
gdTcells/TCR Vg9+ TCR-DV2high	B	(180,360]	0.0284	0.00122	0.0288	0.0725	0.202	*
gdTcells/TCR Vg9+ TCR-DV2high	C	(0,14]	0.000744	1.26E-05	0.000724	0.137	0.579	**
gdTcells/TCR Vg9+ TCR-DV2high	C	(14,30]	0.481	0.000712	0.452	0.293	0.678	
gdTcells/TCR Vg9+ TCR-DV2high	C	(30,90]	0.808	5.42E-05	0.804	0.571	0.839	
gdTcells/TCR Vg9+ TCR-DV2high	C	(90,180]	0.594	3.79E-05	0.603	0.121	0.498	
gdTcells/TCR Vg9+ TCR-DV2high	C	(180,360]	0.956	0.000657	0.893	0.24	0.56	
gdTcells/TCR Vg9+ TCR-DV2high	D	(0,14]	0.000176	0.254	5.90E-05	0.00537	0.00918	***
gdTcells/TCR Vg9+ TCR-DV2high	D	(14,30]	0.0419	0.0312	0.0155	0.0161	0.0468	*
gdTcells/TCR Vg9+ TCR-DV2high	D	(30,90]	0.193	0.0139	0.248	0.0198	0.0553	
gdTcells/TCR Vg9+ TCR-DV2high	D	(90,180]	0.251	0.182	0.259	0.00611	0.0116	
gdTcells/TCR Vg9+ TCR-DV2high	D	(180,360]	0.00316	0.000765	0.00353	0.124	0.334	**
gdTcells/TCR Vg9+ TCR-DV2high	E	(0,14]	5.89E-06	0.0315	8.11E-08	0.000253	0.00108	***
gdTcells/TCR Vg9+ TCR-DV2high	E	(14,30]	8.66E-06	0.0849	1.65E-05	0.0921	0.178	***
gdTcells/TCR Vg9+ TCR-DV2high	E	(30,90]	8.05E-06	0.0179	1.97E-05	0.0243	0.064	***
gdTcells/TCR Vg9+ TCR-DV2high	E	(90,180]	0.0771	0.000193	0.0846	0.404	0.891	
gdTcells/TCR Vg9+ TCR-DV2high	E	(180,360]	0.0129	0.00135	0.0105	0.0649	0.19	*
gdTcells/TCR Vg9+ TCR-DV2lo	A	(0,14]	0.0158	0.0178	0.0159	0.434	0.687	*
gdTcells/TCR Vg9+ TCR-DV2lo	A	(14,30]	0.0171	0.0135	0.0168	0.323	0.555	*
gdTcells/TCR Vg9+ TCR-DV2lo	A	(30,90]	0.0464	0.0113	0.0509	0.495	0.789	*
gdTcells/TCR Vg9+ TCR-DV2lo	A	(90,180]	0.385	0.0363	0.362	0.214	0.357	
gdTcells/TCR Vg9+ TCR-DV2lo	B	(0,14]	0.00159	0.00627	0.00215	0.653	0.989	**
gdTcells/TCR Vg9+ TCR-DV2lo	B	(14,30]	0.00388	0.000709	0.00652	0.379	0.756	**
gdTcells/TCR Vg9+ TCR-DV2lo	B	(30,90]	0.116	0.00309	0.156	0.296	0.573	
gdTcells/TCR Vg9+ TCR-DV2lo	B	(90,180]	0.678	0.00265	0.728	0.437	0.789	
gdTcells/TCR Vg9+ TCR-DV2lo	B	(180,360]	0.387	0.0672	0.396	0.688	0.913	
gdTcells/TCR Vg9+ TCR-DV2lo	C	(0,14]	0.0456	0.0438	0.0324	0.0756	0.161	*
gdTcells/TCR Vg9+ TCR-DV2lo	C	(14,30]	0.666	0.0161	0.624	1	0.651	
gdTcells/TCR Vg9+ TCR-DV2lo	C	(30,90]	0.593	0.0115	0.609	0.36	0.634	
gdTcells/TCR Vg9+ TCR-DV2lo	C	(90,180]	0.51	0.0725	0.496	0.111	0.209	
gdTcells/TCR Vg9+ TCR-DV2lo	C	(180,360]	0.0778	0.0109	0.0866	0.72	0.936	
gdTcells/TCR Vg9+ TCR-DV2lo	D	(0,14]	0.00272	0.231	0.000993	0.00229	0.00416	**
gdTcells/TCR Vg9+ TCR-DV2lo	D	(14,30]	0.255	0.0393	0.171	0.081	0.186	
gdTcells/TCR Vg9+ TCR-DV2lo	D	(30,90]	0.0836	0.00493	0.0977	0.285	0.584	
gdTcells/TCR Vg9+ TCR-DV2lo	D	(90,180]	0.137	0.0207	0.135	0.296	0.577	
gdTcells/TCR Vg9+ TCR-DV2lo	D	(180,360]	0.393	0.0224	0.387	0.325	0.543	
gdTcells/TCR Vg9+ TCR-DV2lo	E	(0,14]	0.0404	0.0157	0.0265	0.109	0.291	*
gdTcells/TCR Vg9+ TCR-DV2lo	E	(14,30]	0.00758	0.0304	0.0104	0.294	0.555	*
gdTcells/TCR Vg9+ TCR-DV2lo	E	(30,90]	0.0384	0.0792	0.0632	0.0846	0.153	*
gdTcells/TCR Vg9+ TCR-DV2lo	E	(90,180]	0.253	0.0346	0.201	0.101	0.197	
gdTcells/TCR Vg9+ TCR-DV2lo	E	(180,360]	0.623	0.00871	0.648	0.275	0.515	
MAIT	A	(0,14]	0.258	0.101	0.215	0.525	0.362	
MAIT	A	(14,30]	0.429	0.101	0.401	0.629	0.446	
MAIT	A	(30,90]	0.108	0.348	0.116	0.803	0.684	
MAIT	A	(90,180]	0.412	0.181	0.394	0.674	0.52	
MAIT	B	(0,14]	0.569	0.0236	0.692	0.25	0.437	
MAIT	B	(14,30]	0.969	0.0526	0.932	0.602	0.84	
MAIT	B	(30,90]	0.743	0.018	0.798	0.477	0.746	
MAIT	B	(90,180]	0.201	0.126	0.209	0.738	0.938	
MAIT	B	(180,360]	0.512	0.123	0.516	0.949	0.85	
MAIT	C	(0,14]	0.0254	0.00238	0.0343	0.604	0.239	*
MAIT	C	(14,30]	0.378	0.0441	0.539	0.171	0.0774	
MAIT	C	(30,90]	0.875	0.0203	0.949	0.46	0.242	
MAIT	C	(90,180]	0.762	0.000475	0.735	0.0798	0.0109	
MAIT	C	(180,360]	0.571	0.0987	0.693	0.351	0.219	
MAIT	D	(0,14]	3.33E-09	0.017	4.80E-09	0.938	0.532	***
MAIT	D	(14,30]	0.000196	0.144	0.000411	0.552	0.344	***
MAIT	D	(30,90]	0.00933	0.0787	0.00775	0.562	0.356	*
MAIT	D	(90,180]	0.0348	0.0352	0.0335	0.723	0.404	*
MAIT	D	(180,360]	0.164	0.157	0.149	0.604	0.444	
MAIT	E	(0,14]	1.47E-09	0.209	5.08E-09	0.967	0.79	***
MAIT	E	(14,30]	1.97E-11	0.303	3.35E-11	0.878	0.7	***
MAIT	E	(30,90]	2.77E-10	0.167	7.99E-10	0.228	0.331	***

MAIT	E	(90,180]	0.00152	0.142	0.00194	0.659	0.853	**
MAIT	E	(180,360]	0.723	0.0405	0.695	0.374	0.21	
abs_NKT	A	(0,14]	0.356	0.444	0.357	0.795	0.89	
abs_NKT	A	(14,30]	0.145	0.882	0.138	0.565	0.576	
abs_NKT	A	(30,90]	0.759	0.952	0.757	0.817	0.822	
abs_NKT	A	(90,180]	0.46	0.562	0.443	0.458	0.511	
abs_NKT	B	(0,14]	0.652	0.929	0.67	0.949	0.96	
abs_NKT	B	(14,30]	0.031	0.744	0.033	0.894	0.851	*
abs_NKT	B	(30,90]	0.324	0.723	0.285	0.539	0.573	
abs_NKT	B	(90,180]	0.769	0.787	0.767	0.924	0.959	
abs_NKT	B	(180,360]	0.729	0.647	0.729	0.833	0.889	
abs_NKT	C	(0,14]	0.459	0.599	0.528	0.237	0.188	
abs_NKT	C	(14,30]	0.129	0.736	0.0976	0.354	0.315	
abs_NKT	C	(30,90]	0.362	0.599	0.39	0.428	0.369	
abs_NKT	C	(90,180]	0.778	0.318	0.786	0.195	0.126	
abs_NKT	C	(180,360]	0.0843	0.981	0.093	0.872	0.867	
abs_NKT	D	(0,14]	0.902	0.773	0.923	0.661	0.608	
abs_NKT	D	(14,30]	0.0439	0.946	0.0532	0.78	0.763	*
abs_NKT	D	(30,90]	0.109	0.917	0.117	0.786	0.767	
abs_NKT	D	(90,180]	0.138	0.207	0.139	0.232	0.134	
abs_NKT	D	(180,360]	0.0209	0.957	0.0228	0.86	0.865	*
abs_NKT	E	(0,14]	0.0129	0.812	0.0142	0.821	0.771	*
abs_NKT	E	(14,30]	0.221	0.568	0.223	0.816	0.911	
abs_NKT	E	(30,90]	0.392	0.408	0.47	0.494	0.396	
abs_NKT	E	(90,180]	0.739	0.841	0.729	0.765	0.789	
abs_NKT	E	(180,360]	0.965	0.897	0.953	0.831	0.811	
abs_NK	A	(0,14]	0.201	0.326	0.214	0.989	0.884	
abs_NK	A	(14,30]	0.0664	0.695	0.0698	0.907	0.957	
abs_NK	A	(30,90]	0.667	0.591	0.678	0.802	0.732	
abs_NK	A	(90,180]	0.451	0.277	0.457	0.855	0.993	
abs_NK	B	(0,14]	0.245	0.15	0.295	0.552	0.718	
abs_NK	B	(14,30]	0.288	0.0393	0.35	0.416	0.629	
abs_NK	B	(30,90]	0.529	0.218	0.577	0.625	0.77	
abs_NK	B	(90,180]	0.284	0.275	0.299	0.857	0.998	
abs_NK	B	(180,360]	0.0407	0.295	0.0437	0.954	0.911	*
abs_NK	C	(0,14]	0.0299	0.147	0.0308	0.909	0.852	*
abs_NK	C	(14,30]	0.621	0.249	0.644	0.746	0.907	
abs_NK	C	(30,90]	0.0659	0.88	0.0747	0.333	0.312	
abs_NK	C	(90,180]	0.608	0.964	0.595	0.162	0.156	
abs_NK	C	(180,360]	0.937	0.466	0.92	0.733	0.825	
abs_NK	D	(0,14]	0.00286	0.264	0.00273	0.629	0.457	**
abs_NK	D	(14,30]	0.379	0.899	0.461	0.342	0.344	
abs_NK	D	(30,90]	0.0492	0.712	0.0553	0.534	0.572	*
abs_NK	D	(90,180]	0.325	0.264	0.333	0.655	0.483	
abs_NK	D	(180,360]	0.325	0.44	0.332	0.958	0.854	
abs_NK	E	(0,14]	1.13E-05	0.52	2.42E-05	0.848	0.967	***
abs_NK	E	(14,30]	0.0814	0.704	0.0739	0.476	0.518	
abs_NK	E	(30,90]	0.512	0.234	0.655	0.234	0.153	
abs_NK	E	(90,180]	0.658	0.346	0.683	0.767	0.638	
abs_NK	E	(180,360]	0.0891	0.823	0.103	0.35	0.323	
CD19+/CD19 naive	A	(0,14]	0.398	0.49	0.374	0.502	0.57	
CD19+/CD19 naive	A	(14,30]	0.545	0.272	0.511	0.315	0.401	
CD19+/CD19 naive	A	(30,90]	0.46	0.393	0.479	0.444	0.525	
CD19+/CD19 naive	A	(90,180]	0.509	0.767	0.455	0.293	0.308	
CD19+/CD19 naive	B	(0,14]	0.0882	0.977	0.127	0.605	0.603	
CD19+/CD19 naive	B	(14,30]	0.814	0.989	0.871	0.767	0.763	
CD19+/CD19 naive	B	(30,90]	0.862	0.457	0.995	0.375	0.437	
CD19+/CD19 naive	B	(90,180]	0.671	0.41	0.791	0.427	0.505	
CD19+/CD19 naive	B	(180,360]	0.63	0.825	0.627	0.667	0.689	
CD19+/CD19 naive	C	(0,14]	0.00441	0.189	0.00529	0.901	0.687	**
CD19+/CD19 naive	C	(14,30]	0.836	0.374	0.778	0.744	0.62	
CD19+/CD19 naive	C	(30,90]	0.364	0.0887	0.351	0.821	0.593	
CD19+/CD19 naive	C	(90,180]	0.381	0.0655	0.382	0.508	0.287	
CD19+/CD19 naive	C	(180,360]	0.886	0.248	0.931	0.794	0.643	
CD19+/CD19 naive	D	(0,14]	0.00907	0.186	0.0103	0.901	0.683	*
CD19+/CD19 naive	D	(14,30]	0.692	0.192	0.761	0.748	0.532	
CD19+/CD19 naive	D	(30,90]	0.125	0.268	0.113	0.531	0.398	
CD19+/CD19 naive	D	(90,180]	0.709	0.0205	0.67	0.135	0.041	
CD19+/CD19 naive	D	(180,360]	0.788	0.346	0.789	0.907	0.781	
CD19+/CD19 naive	E	(0,14]	0.0284	0.0962	0.0782	0.117	0.0433	*
CD19+/CD19 naive	E	(14,30]	0.631	0.0713	0.457	0.117	0.0442	
CD19+/CD19 naive	E	(30,90]	0.431	0.481	0.45	0.814	0.914	
CD19+/CD19 naive	E	(90,180]	0.172	0.232	0.167	0.854	0.687	
CD19+/CD19 naive	E	(180,360]	0.275	0.196	0.291	0.844	0.669	
CD19+/CD19 naive/Transitional	A	(0,14]	0.852	0.27	0.838	0.702	0.835	
CD19+/CD19 naive/Transitional	A	(14,30]	0.0118	0.586	0.0139	0.113	0.129	*
CD19+/CD19 naive/Transitional	A	(30,90]	0.0921	0.61	0.0771	0.115	0.129	
CD19+/CD19 naive/Transitional	A	(90,180]	0.123	0.932	0.152	0.0766	0.0752	

CD19+/CD19 naive/Transitional	B	(0,14]	0.593	0.533	0.526	0.701	0.622	
CD19+/CD19 naive/Transitional	B	(14,30]	0.0133	0.877	0.00654	0.178	0.164	*
CD19+/CD19 naive/Transitional	B	(30,90]	0.0556	0.906	0.027	0.117	0.116	
CD19+/CD19 naive/Transitional	B	(90,180]	0.128	0.757	0.0558	0.0621	0.0657	
CD19+/CD19 naive/Transitional	B	(180,360]	0.751	0.973	0.775	0.209	0.201	
CD19+/CD19 naive/Transitional	C	(0,14]	0.687	0.254	0.661	0.46	0.605	
CD19+/CD19 naive/Transitional	C	(14,30]	0.793	0.39	0.812	0.79	0.911	
CD19+/CD19 naive/Transitional	C	(30,90]	0.851	0.27	0.887	0.386	0.493	
CD19+/CD19 naive/Transitional	C	(90,180]	0.17	0.134	0.173	0.807	0.573	
CD19+/CD19 naive/Transitional	C	(180,360]	0.425	0.443	0.464	0.572	0.658	
CD19+/CD19 naive/Transitional	D	(0,14]	0.835	0.291	0.834	0.764	0.933	
CD19+/CD19 naive/Transitional	D	(14,30]	0.348	0.354	0.328	0.83	0.668	
CD19+/CD19 naive/Transitional	D	(30,90]	0.0277	0.549	0.0313	0.707	0.786	*
CD19+/CD19 naive/Transitional	D	(90,180]	0.0443	0.151	0.047	0.753	0.523	*
CD19+/CD19 naive/Transitional	D	(180,360]	0.204	0.711	0.207	0.315	0.339	
CD19+/CD19 naive/Transitional	E	(0,14]	0.0151	0.401	0.0236	0.901	0.738	*
CD19+/CD19 naive/Transitional	E	(14,30]	0.191	0.364	0.158	0.47	0.345	
CD19+/CD19 naive/Transitional	E	(30,90]	0.00412	0.275	0.00528	0.71	0.86	**
CD19+/CD19 naive/Transitional	E	(90,180]	0.848	0.494	0.888	0.461	0.531	
CD19+/CD19 naive/Transitional	E	(180,360]	0.934	0.355	0.952	0.594	0.705	
CD19+/IgD-CD27+ memory/CD24+CD38+	A	(0,14]	0.423	0.375	0.439	0.317	0.386	
CD19+/IgD-CD27+ memory/CD24+CD38+	A	(14,30]	0.147	0.566	0.133	0.329	0.37	
CD19+/IgD-CD27+ memory/CD24+CD38+	A	(30,90]	0.161	0.47	0.172	0.427	0.492	
CD19+/IgD-CD27+ memory/CD24+CD38+	A	(90,180]	0.0236	0.15	0.0241	0.56	0.72	*
CD19+/IgD-CD27+ memory/CD24+CD38+	B	(0,14]	0.0106	0.255	0.00815	0.341	0.438	*
CD19+/IgD-CD27+ memory/CD24+CD38+	B	(14,30]	0.277	0.38	0.218	0.348	0.419	
CD19+/IgD-CD27+ memory/CD24+CD38+	B	(30,90]	0.937	0.703	0.811	0.407	0.437	
CD19+/IgD-CD27+ memory/CD24+CD38+	B	(90,180]	0.744	0.962	0.655	0.555	0.554	
CD19+/IgD-CD27+ memory/CD24+CD38+	B	(180,360]	0.625	0.328	0.643	0.308	0.382	
CD19+/IgD-CD27+ memory/CD24+CD38+	C	(0,14]	0.00112	0.84	0.00171	0.112	0.0963	**
CD19+/IgD-CD27+ memory/CD24+CD38+	C	(14,30]	0.259	0.81	0.411	0.0353	0.036	
CD19+/IgD-CD27+ memory/CD24+CD38+	C	(30,90]	0.616	0.563	0.525	0.089	0.0677	
CD19+/IgD-CD27+ memory/CD24+CD38+	C	(90,180]	0.81	0.322	0.809	0.0858	0.0502	
CD19+/IgD-CD27+ memory/CD24+CD38+	C	(180,360]	0.798	0.71	0.686	0.19	0.205	
CD19+/IgD-CD27+ memory/CD24+CD38+	D	(0,14]	0.00137	0.826	0.0016	0.346	0.359	**
CD19+/IgD-CD27+ memory/CD24+CD38+	D	(14,30]	0.00998	0.289	0.0126	0.65	0.821	*
CD19+/IgD-CD27+ memory/CD24+CD38+	D	(30,90]	0.00242	0.73	0.00165	0.14	0.151	**
CD19+/IgD-CD27+ memory/CD24+CD38+	D	(90,180]	0.0867	0.204	0.0517	0.00479	0.00164	
CD19+/IgD-CD27+ memory/CD24+CD38+	D	(180,360]	0.102	0.569	0.1	0.257	0.292	
CD19+/IgD-CD27+ memory/CD24+CD38+	E	(0,14]	0.00081	0.731	0.00206	0.0838	0.0899	**
CD19+/IgD-CD27+ memory/CD24+CD38+	E	(14,30]	0.231	0.854	0.156	0.103	0.0875	
CD19+/IgD-CD27+ memory/CD24+CD38+	E	(30,90]	0.00903	0.154	0.014	0.68	0.495	*
CD19+/IgD-CD27+ memory/CD24+CD38+	E	(90,180]	0.656	0.701	0.665	0.999	0.946	
CD19+/IgD-CD27+ memory/CD24+CD38+	E	(180,360]	0.188	0.553	0.205	0.449	0.506	
Plasmablasts	A	(0,14]	0.000498	0.297	0.00059	0.853	0.991	***
Plasmablasts	A	(14,30]	0.00307	0.514	0.00307	0.652	0.573	**
Plasmablasts	A	(30,90]	0.124	0.299	0.132	0.882	0.748	
Plasmablasts	A	(90,180]	0.0372	0.401	0.0465	0.319	0.253	*
Plasmablasts	B	(0,14]	9.58E-09	0.965	5.16E-08	0.434	0.423	***
Plasmablasts	B	(14,30]	0.000148	0.996	0.00038	0.446	0.439	***
Plasmablasts	B	(30,90]	0.0485	0.271	0.0697	0.735	0.6	*
Plasmablasts	B	(90,180]	0.0402	0.376	0.0541	0.891	0.773	*
Plasmablasts	B	(180,360]	0.0282	0.779	0.0301	0.784	0.747	*
Plasmablasts	C	(0,14]	3.47E-07	0.945	4.77E-07	0.896	0.883	***
Plasmablasts	C	(14,30]	0.0117	0.296	0.00747	0.318	0.227	*
Plasmablasts	C	(30,90]	0.0302	0.153	0.0357	0.41	0.276	*
Plasmablasts	C	(90,180]	0.424	0.0389	0.424	0.705	0.41	
Plasmablasts	C	(180,360]	0.0187	0.33	0.0236	0.767	0.64	*
Plasmablasts	D	(0,14]	3.03E-07	0.263	3.93E-07	0.994	0.818	***
Plasmablasts	D	(14,30]	2.91E-11	0.802	6.86E-11	0.276	0.289	***
Plasmablasts	D	(30,90]	0.00671	0.692	0.00591	0.337	0.367	*
Plasmablasts	D	(90,180]	0.311	0.588	0.299	0.209	0.243	
Plasmablasts	D	(180,360]	0.39	0.556	0.395	0.667	0.736	
Plasmablasts	E	(0,14]	5.26E-09	0.0878	1.09E-09	0.111	0.043	***
Plasmablasts	E	(14,30]	2.04E-19	0.00546	8.85E-20	0.0532	0.00882	***
Plasmablasts	E	(30,90]	2.01E-09	0.667	4.35E-09	0.949	0.886	***
Plasmablasts	E	(90,180]	0.00138	0.974	0.00125	0.436	0.425	**
Plasmablasts	E	(180,360]	0.0858	0.0802	0.0861	0.959	0.716	
CD19+/MZ_like	A	(0,14]	0.498	0.0797	0.44	0.484	0.319	
CD19+/MZ_like	A	(14,30]	0.125	0.109	0.126	0.991	0.799	
CD19+/MZ_like	A	(30,90]	0.481	0.141	0.495	0.913	0.722	
CD19+/MZ_like	A	(90,180]	0.104	0.144	0.0979	0.726	0.555	
CD19+/MZ_like	B	(0,14]	0.0284	0.891	0.0198	0.383	0.364	*
CD19+/MZ_like	B	(14,30]	0.298	0.458	0.189	0.228	0.179	
CD19+/MZ_like	B	(30,90]	0.942	0.293	0.767	0.375	0.284	
CD19+/MZ_like	B	(90,180]	0.589	0.185	0.437	0.373	0.259	
CD19+/MZ_like	B	(180,360]	0.385	0.404	0.4	0.438	0.357	

CD19+/MZ_like	C	(0,14]	0.00545	0.102	0.00702	0.288	0.153	*
CD19+/MZ_like	C	(14,30]	0.555	0.0915	0.319	0.0515	0.0221	
CD19+/MZ_like	C	(30,90]	0.61	0.0542	0.689	0.129	0.0597	
CD19+/MZ_like	C	(90,180]	0.596	0.0769	0.593	0.206	0.0964	
CD19+/MZ_like	C	(180,360]	0.619	0.156	0.496	0.206	0.127	
CD19+/MZ_like	D	(0,14]	0.00182	0.148	0.00212	0.509	0.326	**
CD19+/MZ_like	D	(14,30]	0.062	0.287	0.0773	0.801	0.617	
CD19+/MZ_like	D	(30,90]	0.0168	0.102	0.0113	0.205	0.11	*
CD19+/MZ_like	D	(90,180]	0.1	0.00879	0.0784	0.102	0.0239	
CD19+/MZ_like	D	(180,360]	0.104	0.178	0.0988	0.295	0.197	
CD19+/MZ_like	E	(0,14]	0.00467	0.172	0.0109	0.526	0.323	**
CD19+/MZ_like	E	(14,30]	0.281	0.14	0.215	0.333	0.185	
CD19+/MZ_like	E	(30,90]	0.0212	0.516	0.0324	0.44	0.365	*
CD19+/MZ_like	E	(90,180]	0.154	0.299	0.16	0.829	0.975	
CD19+/MZ_like	E	(180,360]	0.0984	0.279	0.11	0.533	0.41	
pDC	A	(0,14]	0.251	0.625	0.273	0.659	0.713	
pDC	A	(14,30]	0.0156	0.434	0.0186	0.189	0.229	*
pDC	A	(30,90]	0.325	0.896	0.291	0.132	0.132	
pDC	A	(90,180]	0.338	0.834	0.359	0.492	0.508	
pDC	B	(0,14]	0.975	0.97	0.692	0.137	0.13	
pDC	B	(14,30]	0.181	0.973	0.097	0.12	0.117	
pDC	B	(30,90]	0.337	0.15	0.145	0.0116	0.02	
pDC	B	(90,180]	0.703	0.741	0.925	0.0307	0.0326	
pDC	B	(180,360]	0.865	0.9	0.833	0.175	0.163	
pDC	C	(0,14]	0.365	0.64	0.362	0.762	0.833	
pDC	C	(14,30]	0.235	0.361	0.183	0.409	0.314	
pDC	C	(30,90]	0.101	0.908	0.098	0.604	0.613	
pDC	C	(90,180]	0.824	0.856	0.825	0.804	0.829	
pDC	C	(180,360]	0.669	0.461	0.77	0.312	0.25	
pDC	D	(0,14]	0.0128	0.29	0.0116	0.485	0.342	*
pDC	D	(14,30]	0.024	0.751	0.0226	0.655	0.596	*
pDC	D	(30,90]	0.0982	0.371	0.117	0.11	0.0729	
pDC	D	(90,180]	0.0125	0.569	0.0131	0.383	0.31	*
pDC	D	(180,360]	0.0609	0.65	0.0604	0.253	0.217	
pDC	E	(0,14]	0.00255	0.552	0.00123	0.269	0.2	**
pDC	E	(14,30]	1.25E-08	0.492	2.21E-08	0.292	0.217	***
pDC	E	(30,90]	2.00E-06	0.335	4.48E-06	0.0274	0.0157	***
pDC	E	(90,180]	0.011	0.373	0.0115	0.0508	0.0324	*
pDC	E	(180,360]	0.0414	0.56	0.032	0.308	0.256	*
mDC	A	(0,14]	0.472	0.872	0.398	0.223	0.207	
mDC	A	(14,30]	0.0279	0.641	0.021	0.181	0.153	*
mDC	A	(30,90]	0.967	0.843	0.948	0.541	0.514	
mDC	A	(90,180]	0.851	0.261	0.837	0.551	0.672	
mDC	B	(0,14]	0.0186	0.153	0.0186	0.547	0.711	*
mDC	B	(14,30]	0.174	0.619	0.252	0.372	0.41	
mDC	B	(30,90]	0.00705	0.0863	0.0159	0.112	0.183	*
mDC	B	(90,180]	0.0324	0.378	0.0244	0.339	0.411	*
mDC	B	(180,360]	0.566	0.476	0.542	0.199	0.235	
mDC	C	(0,14]	5.32E-05	0.514	6.70E-05	0.81	0.913	***
mDC	C	(14,30]	0.932	0.653	0.666	0.0351	0.0393	
mDC	C	(30,90]	0.111	0.605	0.128	0.0353	0.0261	
mDC	C	(90,180]	0.74	0.394	0.714	0.0064	0.00314	
mDC	C	(180,360]	0.0373	0.326	0.0478	0.385	0.475	*
mDC	D	(0,14]	6.09E-07	0.123	7.75E-07	0.904	0.824	***
mDC	D	(14,30]	0.00074	0.79	0.0012	0.0553	0.0568	**
mDC	D	(30,90]	0.126	0.922	0.148	0.191	0.189	
mDC	D	(90,180]	0.966	0.67	0.97	0.704	0.767	
mDC	D	(180,360]	0.133	0.748	0.132	0.177	0.189	
mDC	E	(0,14]	7.41E-06	0.888	5.42E-05	0.148	0.129	***
mDC	E	(14,30]	0.00412	0.73	0.0037	0.451	0.487	**
mDC	E	(30,90]	0.873	0.538	0.967	0.488	0.413	
mDC	E	(90,180]	0.073	0.49	0.0749	0.756	0.847	
mDC	E	(180,360]	0.502	0.912	0.394	0.104	0.103	
classical monocytes	A	(0,14]	0.154	0.83	0.201	0.0652	0.0668	
classical monocytes	A	(14,30]	0.888	0.63	0.832	0.256	0.283	
classical monocytes	A	(30,90]	0.944	0.838	0.896	0.124	0.127	
classical monocytes	A	(90,180]	0.896	0.981	0.817	0.0649	0.0611	
classical monocytes	B	(0,14]	0.0341	0.866	0.083	0.141	0.129	*
classical monocytes	B	(14,30]	0.644	0.681	0.779	0.488	0.442	
classical monocytes	B	(30,90]	0.263	0.23	0.474	0.029	0.0434	
classical monocytes	B	(90,180]	0.00329	0.442	0.0102	0.0544	0.0682	**
classical monocytes	B	(180,360]	0.767	0.83	0.729	0.124	0.127	
classical monocytes	C	(0,14]	0.111	0.486	0.114	0.961	0.924	
classical monocytes	C	(14,30]	0.727	0.83	0.876	0.269	0.245	
classical monocytes	C	(30,90]	0.841	0.544	0.878	0.432	0.489	
classical monocytes	C	(90,180]	0.502	0.828	0.49	0.0928	0.0782	
classical monocytes	C	(180,360]	0.109	0.872	0.139	0.292	0.298	

classical monocytes	D	(0,14]	0.00931	0.474	0.00756	0.259	0.189	*
classical monocytes	D	(14,30]	0.0653	0.311	0.0133	0.00388	0.00153	
classical monocytes	D	(30,90]	0.86	0.584	0.846	0.71	0.783	
classical monocytes	D	(90,180]	0.276	0.538	0.279	0.102	0.0713	
classical monocytes	D	(180,360]	0.397	0.954	0.387	0.0625	0.0577	
classical monocytes	E	(0,14]	0.115	0.106	0.306	0.203	0.0884	
classical monocytes	E	(14,30]	0.686	0.754	0.663	0.779	0.72	
classical monocytes	E	(30,90]	0.107	0.954	0.0829	0.333	0.32	
classical monocytes	E	(90,180]	0.229	0.926	0.188	0.107	0.0982	
classical monocytes	E	(180,360]	0.216	0.677	0.282	0.0795	0.0643	
CD4activated/naive	A	(0,14]	0.281	0.0116	0.311	0.952	0.724	
CD4activated/naive	A	(14,30]	0.133	0.0083	0.149	0.978	0.678	
CD4activated/naive	A	(30,90]	0.196	0.0284	0.191	0.756	0.503	
CD4activated/naive	A	(90,180]	0.0748	0.0695	0.0817	0.878	0.891	
CD4activated/naive	B	(0,14]	0.00438	0.00398	0.00385	0.882	0.538	**
CD4activated/naive	B	(14,30]	0.00391	0.00119	0.00316	0.822	0.457	**
CD4activated/naive	B	(30,90]	0.109	0.00287	0.0951	0.917	0.561	
CD4activated/naive	B	(90,180]	0.773	0.00467	0.742	0.829	0.803	
CD4activated/naive	C	(0,14]	0.0705	0.122	0.0593	0.259	0.407	
CD4activated/naive	C	(14,30]	0.277	0.0393	0.309	0.796	0.896	
CD4activated/naive	C	(30,90]	0.161	0.0423	0.166	0.966	0.68	
CD4activated/naive	C	(90,180]	0.727	0.0138	0.755	0.97	0.623	
CD4activated/naive	C	(180,360]	0.646	0.0492	0.641	0.982	0.717	
CD4activated/naive	D	(0,14]	0.115	0.031	0.137	0.793	0.801	
CD4activated/naive	D	(14,30]	0.0506	0.0126	0.0949	0.759	0.353	
CD4activated/naive	D	(30,90]	0.245	0.0325	0.25	0.817	0.854	
CD4activated/naive	D	(90,180]	0.594	0.0283	0.593	0.459	0.8	
CD4activated/naive	D	(180,360]	0.61	0.0259	0.554	0.494	0.282	
CD4activated/naive	E	(0,14]	0.215	0.047	0.305	0.926	0.671	
CD4activated/naive	E	(14,30]	0.0198	0.0125	0.0187	0.781	0.38	*
CD4activated/naive	E	(30,90]	0.0388	0.0185	0.0281	0.318	0.142	*
CD4activated/naive	E	(90,180]	0.102	0.072	0.102	0.46	0.28	
CD4activated/naive	E	(180,360]	0.0262	0.0235	0.0398	0.574	0.329	*
CD8activated/naive	A	(0,14]	0.236	1.45E-06	0.271	0.688	0.631	
CD8activated/naive	A	(14,30]	0.203	5.32E-06	0.209	0.369	0.94	
CD8activated/naive	A	(30,90]	0.832	1.52E-05	0.838	0.522	0.898	
CD8activated/naive	A	(90,180]	0.0977	5.11E-05	0.102	0.401	0.894	
CD8activated/naive	B	(0,14]	0.00317	6.04E-08	0.00299	0.667	0.594	**
CD8activated/naive	B	(14,30]	0.0657	6.06E-08	0.0525	0.836	0.462	
CD8activated/naive	B	(30,90]	0.606	5.19E-07	0.654	0.563	0.775	
CD8activated/naive	B	(90,180]	0.498	2.99E-06	0.504	0.444	0.958	
CD8activated/naive	C	(0,14]	0.258	7.52E-07	0.263	0.228	0.943	
CD8activated/naive	C	(14,30]	0.687	4.62E-05	0.697	0.401	0.993	
CD8activated/naive	C	(30,90]	0.794	6.52E-05	0.797	0.439	0.994	
CD8activated/naive	C	(90,180]	0.169	1.27E-08	0.155	0.565	0.406	
CD8activated/naive	C	(180,360]	0.636	0.000128	0.646	0.373	0.847	
CD8activated/naive	D	(0,14]	0.144	7.90E-06	0.136	0.129	0.696	
CD8activated/naive	D	(14,30]	0.000156	0.00344	4.44E-05	0.0176	0.0816	***
CD8activated/naive	D	(30,90]	0.0472	1.35E-05	0.0501	0.444	0.874	*
CD8activated/naive	D	(90,180]	0.427	2.07E-06	0.422	0.0947	0.61	
CD8activated/naive	D	(180,360]	0.276	0.000148	0.288	0.452	0.954	
CD8activated/naive	E	(0,14]	0.252	0.000547	0.115	0.0198	0.136	
CD8activated/naive	E	(14,30]	0.0433	5.51E-06	0.0467	0.182	0.834	*
CD8activated/naive	E	(30,90]	3.18E-05	2.23E-06	3.50E-05	0.511	0.761	***
CD8activated/naive	E	(90,180]	0.211	0.00132	0.21	0.0905	0.264	
CD8activated/naive	E	(180,360]	0.0348	8.79E-06	0.0471	0.749	0.574	*

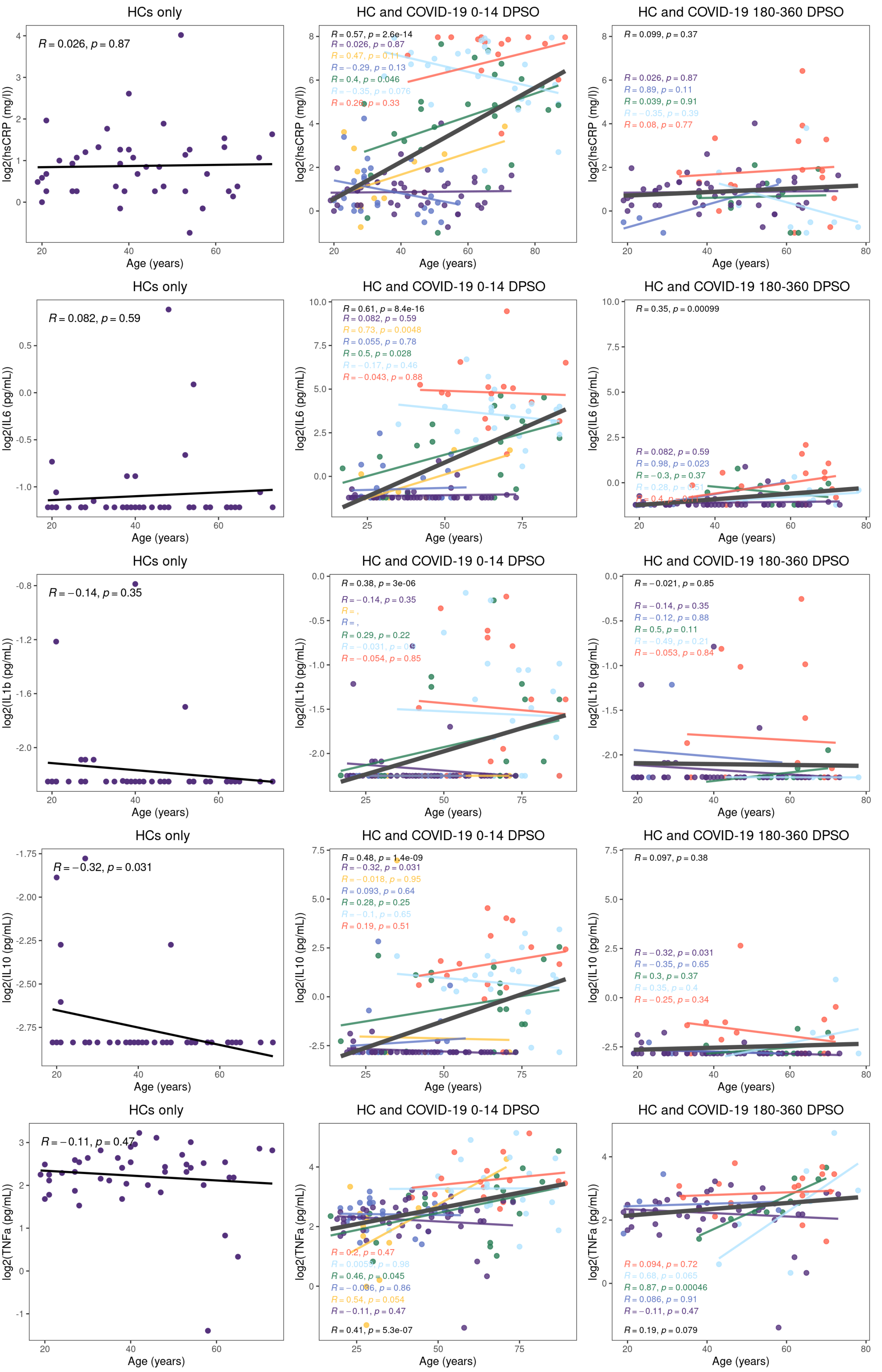

HCs only
R = 0.026, p = 0.87
log2(hsCRP (mg/l))
Age (years)
HC and COVID-19 0-14 DPSO
R = 0.57, p = 2.6e-14
R = 0.026, p = 0.87
R = 0.47, p = 0.11
R = −0.29, p = 0.13
R = 0.4, p = 0.046
R = −0.35, p = 0.076
R = 0.26, p = 0.33
HC and COVID-19 180-360 DPSO
R = 0.099, p = 0.37
R = 0.026, p = 0.87
R = 0.89, p = 0.11
R = 0.039, p = 0.91
R = −0.35, p = 0.39
R = 0.08, p = 0.77
HCs only
R = 0.082, p = 0.59
log2(IL6 (pg/mL))
HC and COVID-19 0-14 DPSO
R = 0.61, p = 8.4e-16
R = 0.082, p = 0.59
R = 0.73, p = 0.0048
R = 0.055, p = 0.78
R = 0.5, p = 0.028
R = −0.17, p = 0.46
R = −0.043, p = 0.88
HC and COVID-19 180-360 DPSO
R = 0.35, p = 0.00099
R = 0.082, p = 0.59
R = 0.98, p = 0.023
R = −0.3, p = 0.37
HCs only
R = −0.14, p = 0.35
log2(IL1b (pg/mL))
HC and COVID-19 0-14 DPSO
R = 0.38, p = 3e-06
R = −0.14, p = 0.35
R = 0.29, p = 0.22
R = −0.054, p = 0.85
HC and COVID-19 180-360 DPSO
R = −0.021, p = 0.85
R = −0.14, p = 0.35
R = −0.12, p = 0.88
R = 0.5, p = 0.11
R = −0.49, p = 0.21
R = −0.053, p = 0.84
HCs only
R = −0.32, p = 0.031
log2(IL10 (pg/mL))
HC and COVID-19 0-14 DPSO
R = 0.48, p = 1.4e-09
R = −0.32, p = 0.031
R = −0.018, p = 0.95
R = 0.093, p = 0.64
R = 0.28, p = 0.25
R = −0.1, p = 0.65
R = 0.19, p = 0.51
HC and COVID-19 180-360 DPSO
R = 0.097, p = 0.38
R = −0.32, p = 0.031
R = −0.35, p = 0.65
R = 0.3, p = 0.37
R = 0.35, p = 0.4
R = −0.25, p = 0.34
HCs only
R = −0.11, p = 0.47
log2(TNFa (pg/mL))
HC and COVID-19 0-14 DPSO
R = 0.2, p = 0.47
R = 0.46, p = 0.045
R = −0.036, p = 0.86
R = 0.54, p = 0.054
R = −0.11, p = 0.47
R = 0.41, p = 5.3e-07
HC and COVID-19 180-360 DPSO
R = 0.094, p = 0.72
R = 0.68, p = 0.065
R = 0.87, p = 0.00046
R = 0.086, p = 0.91
R = −0.11, p = 0.47
R = 0.19, p = 0.079

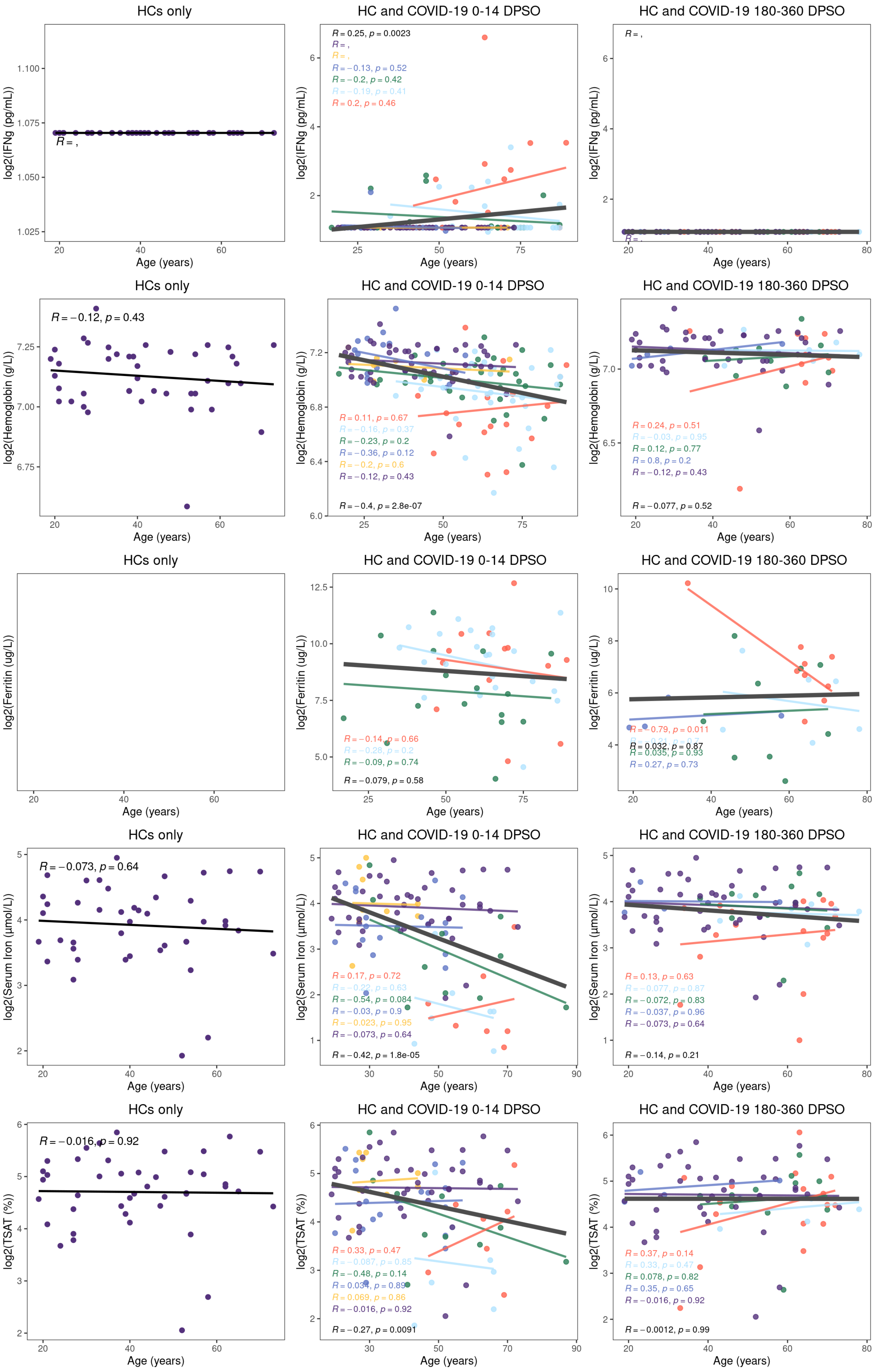

HCs only
HC and COVID-19 0-14 DPSO
HC and COVID-19 180-360 DPSO
log2(IFNg (pg/mL))
R = ,
R = 0.25, p = 0.0023
R = −0.13, p = 0.52
R = −0.2, p = 0.42
R = −0.19, p = 0.41
R = 0.2, p = 0.46
log2(Hemoglobin (g/L))
R = −0.12, p = 0.43
R = 0.11, p = 0.67
R = −0.16, p = 0.37
R = −0.23, p = 0.2
R = −0.36, p = 0.12
R = −0.2, p = 0.6
R = −0.4, p = 2.8e-07
R = 0.24, p = 0.51
R = −0.03, p = 0.95
R = 0.12, p = 0.77
R = 0.8, p = 0.2
R = −0.077, p = 0.52
log2(Ferritin (ug/L))
R = −0.14, p = 0.66
R = −0.28, p = 0.2
R = −0.09, p = 0.74
R = −0.079, p = 0.58
R = −0.79, p = 0.011
R = 0.032, p = 0.87
R = 0.035, p = 0.93
R = 0.27, p = 0.73
log2(Serum Iron (µmol/L))
R = −0.073, p = 0.64
R = 0.17, p = 0.72
R = −0.22, p = 0.63
R = −0.54, p = 0.084
R = −0.03, p = 0.9
R = −0.023, p = 0.95
R = −0.42, p = 1.8e-05
R = 0.13, p = 0.63
R = −0.077, p = 0.87
R = −0.072, p = 0.83
R = −0.037, p = 0.96
R = −0.14, p = 0.21
log2(TSAT (%))
R = −0.016, p = 0.92
R = 0.33, p = 0.47
R = −0.087, p = 0.85
R = −0.48, p = 0.14
R = 0.034, p = 0.89
R = 0.069, p = 0.86
R = −0.27, p = 0.0091
R = 0.37, p = 0.14
R = 0.33, p = 0.47
R = 0.078, p = 0.82
R = 0.35, p = 0.65
R = −0.0012, p = 0.99
Age (years)

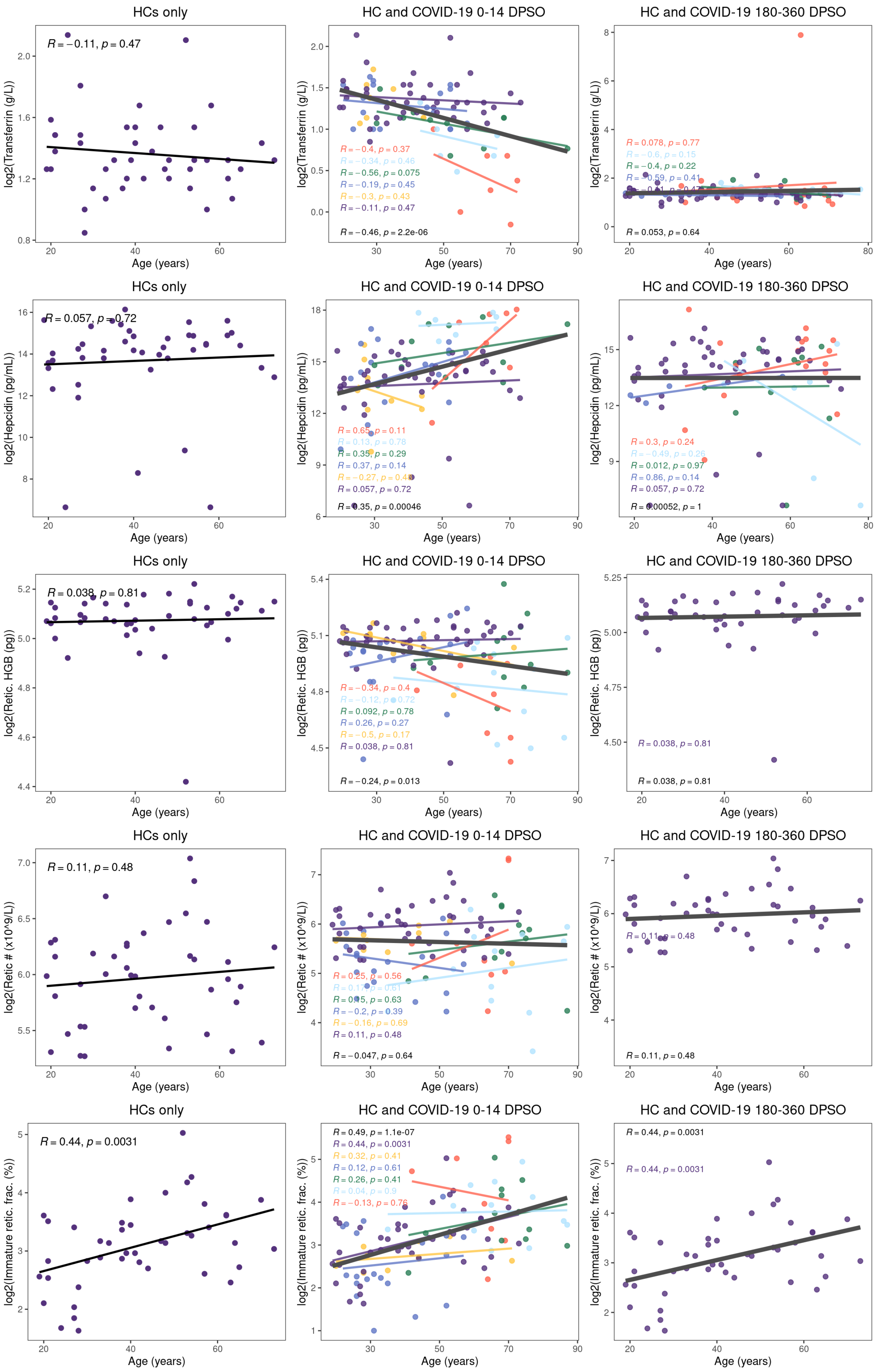
HCs only
R = −0.11, p = 0.47
log2(Transferrin (g/L))
Age (years)
HC and COVID-19 0-14 DPSO
R = −0.4, p = 0.37
R = −0.34, p = 0.46
R = −0.56, p = 0.075
R = −0.19, p = 0.45
R = −0.3, p = 0.43
R = −0.11, p = 0.47
R = −0.46, p = 2.2e-06
log2(Transferrin (g/L))
Age (years)
HC and COVID-19 180-360 DPSO
R = 0.078, p = 0.77
R = −0.6, p = 0.15
R = −0.4, p = 0.22
R = 0.053, p = 0.64
log2(Transferrin (g/L))
Age (years)
HCs only
R = 0.057, p = 0.72
log2(Hepcidin (pg/mL))
Age (years)
HC and COVID-19 0-14 DPSO
R = 0.65, p = 0.11
R = 0.13, p = 0.78
R = 0.35, p = 0.29
R = 0.37, p = 0.14
R = 0.057, p = 0.72
R = 0.35, p = 0.00046
log2(Hepcidin (pg/mL))
Age (years)
HC and COVID-19 180-360 DPSO
R = 0.3, p = 0.24
R = −0.49, p = 0.26
R = 0.012, p = 0.97
R = 0.86, p = 0.14
R = 0.057, p = 0.72
R = 0.00052, p = 1
log2(Hepcidin (pg/mL))
Age (years)
HCs only
R = 0.038, p = 0.81
log2(Retic. HGB (pg))
Age (years)
HC and COVID-19 0-14 DPSO
R = −0.34, p = 0.4
R = −0.12, p = 0.72
R = 0.092, p = 0.78
R = 0.26, p = 0.27
R = −0.5, p = 0.17
R = 0.038, p = 0.81
R = −0.24, p = 0.013
log2(Retic. HGB (pg))
Age (years)
HC and COVID-19 180-360 DPSO
R = 0.038, p = 0.81
R = 0.038, p = 0.81
log2(Retic. HGB (pg))
Age (years)
HCs only
R = 0.11, p = 0.48
log2(Retic # (x10^9/L))
Age (years)
HC and COVID-19 0-14 DPSO
R = 0.25, p = 0.56
R = 0.15, p = 0.63
R = −0.2, p = 0.39
R = −0.16, p = 0.69
R = 0.11, p = 0.48
R = −0.047, p = 0.64
log2(Retic # (x10^9/L))
Age (years)
HC and COVID-19 180-360 DPSO
R = 0.11, p = 0.48
R = 0.11, p = 0.48
log2(Retic # (x10^9/L))
Age (years)
HCs only
R = 0.44, p = 0.0031
log2(Immature retic. frac. (%))
Age (years)
HC and COVID-19 0-14 DPSO
R = 0.49, p = 1.1e-07
R = 0.44, p = 0.0031
R = 0.32, p = 0.41
R = 0.12, p = 0.61
R = 0.26, p = 0.41
R = 0.04, p = 0.9
R = −0.13, p = 0.76
log2(Immature retic. frac. (%))
Age (years)
HC and COVID-19 180-360 DPSO
R = 0.44, p = 0.0031
R = 0.44, p = 0.0031
log2(Immature retic. frac. (%))
Age (years)

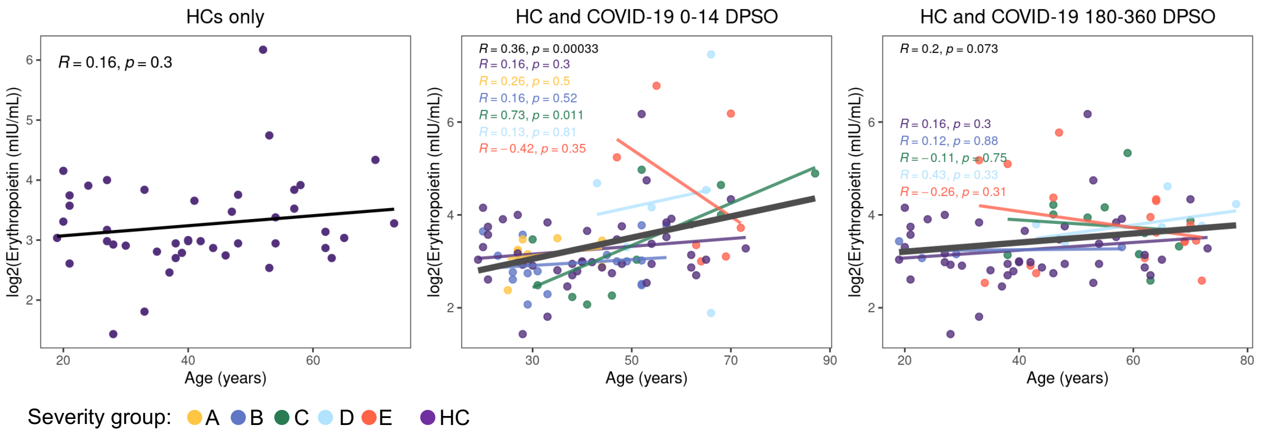

Figure SM1: Pearson's correlation between age and measured clinical parameters in healthy controls (HC; left), within and across COVID-19 severity groups in early (0-14 DPSO; middle) and late (>=180 DPSO; right) disease. Ferritin was not measured in HCs, and reticulocyte parameters were only measured to 180 DPSO, so are lacking data at 270-360 DPSO.

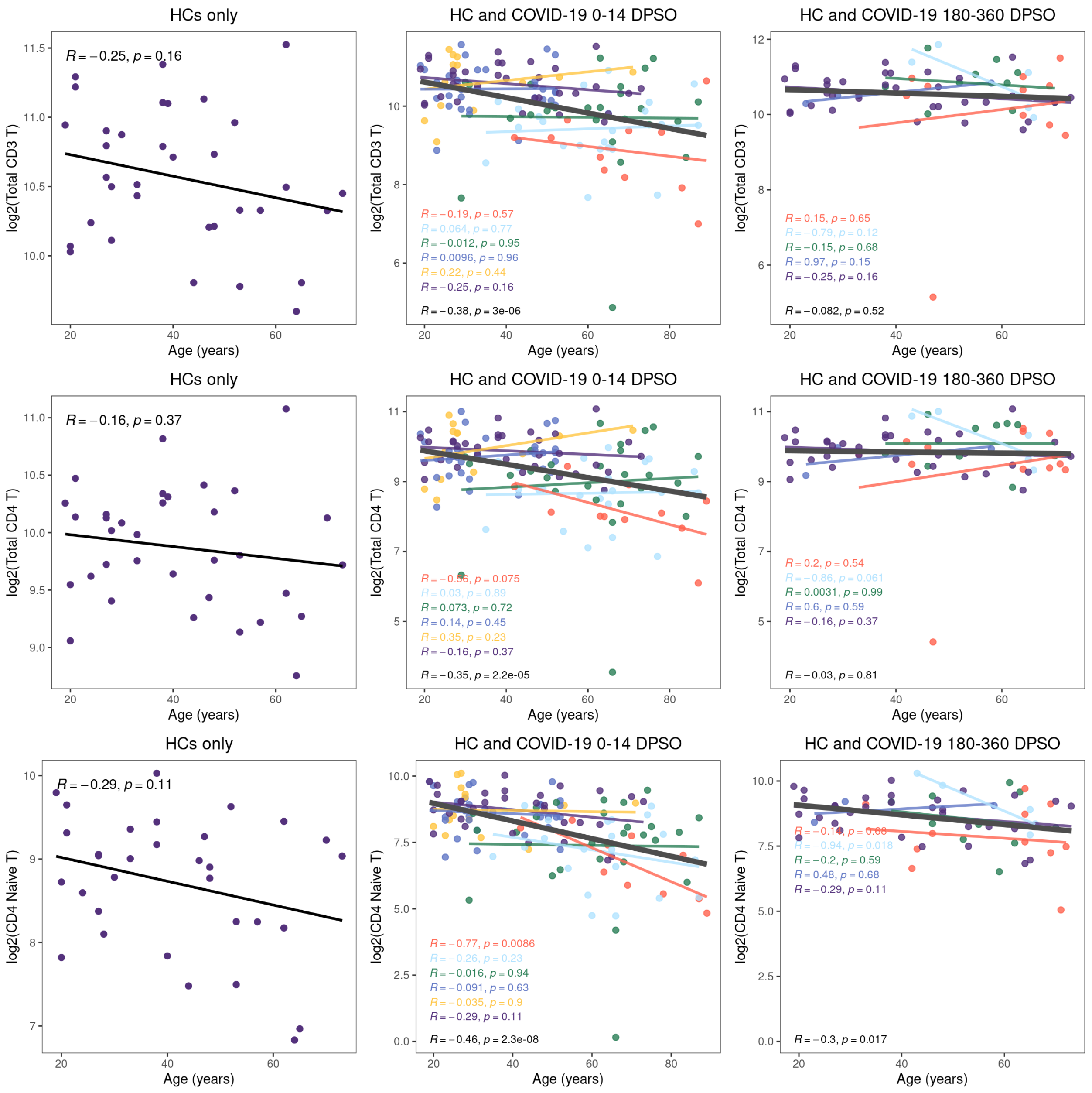

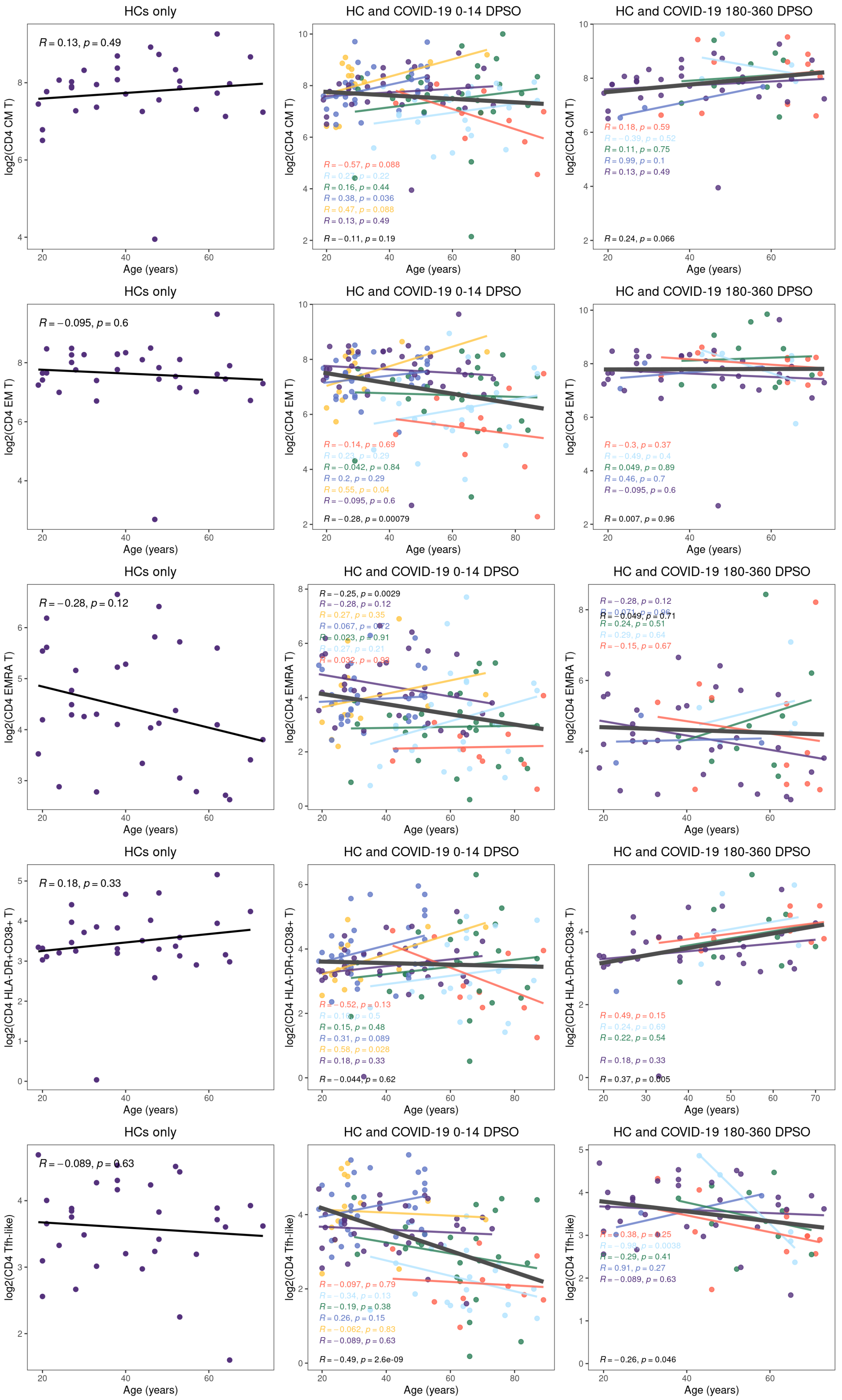

HCs only
HC and COVID-19 0-14 DPSO
HC and COVID-19 180-360 DPSO
log2(CD4 CM T)
log2(CD4 EM T)
log2(CD4 EMRA T)
log2(CD4 HLA-DR+CD38+ T)
log2(CD4 Tfh-like)
Age (years)
R = 0.13, p = 0.49
R = −0.57, p = 0.088
R = 0.16, p = 0.44
R = 0.38, p = 0.036
R = 0.47, p = 0.088
R = 0.13, p = 0.49
R = −0.11, p = 0.19
R = 0.18, p = 0.59
R = −0.39, p = 0.52
R = 0.11, p = 0.75
R = 0.99, p = 0.1
R = 0.13, p = 0.49
R = 0.24, p = 0.066
R = −0.095, p = 0.6
R = −0.14, p = 0.69
R = 0.23, p = 0.29
R = −0.042, p = 0.84
R = 0.2, p = 0.29
R = 0.55, p = 0.04
R = −0.095, p = 0.6
R = −0.28, p = 0.00079
R = −0.3, p = 0.37
R = −0.49, p = 0.4
R = 0.049, p = 0.89
R = 0.46, p = 0.7
R = −0.095, p = 0.6
R = 0.007, p = 0.96
R = −0.28, p = 0.12
R = −0.25, p = 0.0029
R = −0.28, p = 0.12
R = 0.27, p = 0.35
R = 0.067, p = 0.72
R = 0.023, p = 0.91
R = 0.27, p = 0.21
R = 0.032, p = 0.93
R = −0.28, p = 0.12
R = −0.049, p = 0.71
R = 0.24, p = 0.51
R = 0.29, p = 0.64
R = −0.15, p = 0.67
R = 0.18, p = 0.33
R = −0.52, p = 0.13
R = 0.15, p = 0.48
R = 0.31, p = 0.089
R = 0.58, p = 0.028
R = 0.18, p = 0.33
R = −0.044, p = 0.62
R = 0.49, p = 0.15
R = 0.24, p = 0.69
R = 0.22, p = 0.54
R = 0.18, p = 0.33
R = 0.37, p = 0.005
R = −0.089, p = 0.63
R = −0.097, p = 0.79
R = −0.34, p = 0.13
R = −0.19, p = 0.38
R = 0.26, p = 0.15
R = −0.062, p = 0.83
R = −0.089, p = 0.63
R = −0.49, p = 2.6e-09
R = −0.38, p = 0.25
R = −0.98, p = 0.0038
R = −0.29, p = 0.41
R = 0.91, p = 0.27
R = −0.089, p = 0.63
R = −0.26, p = 0.046

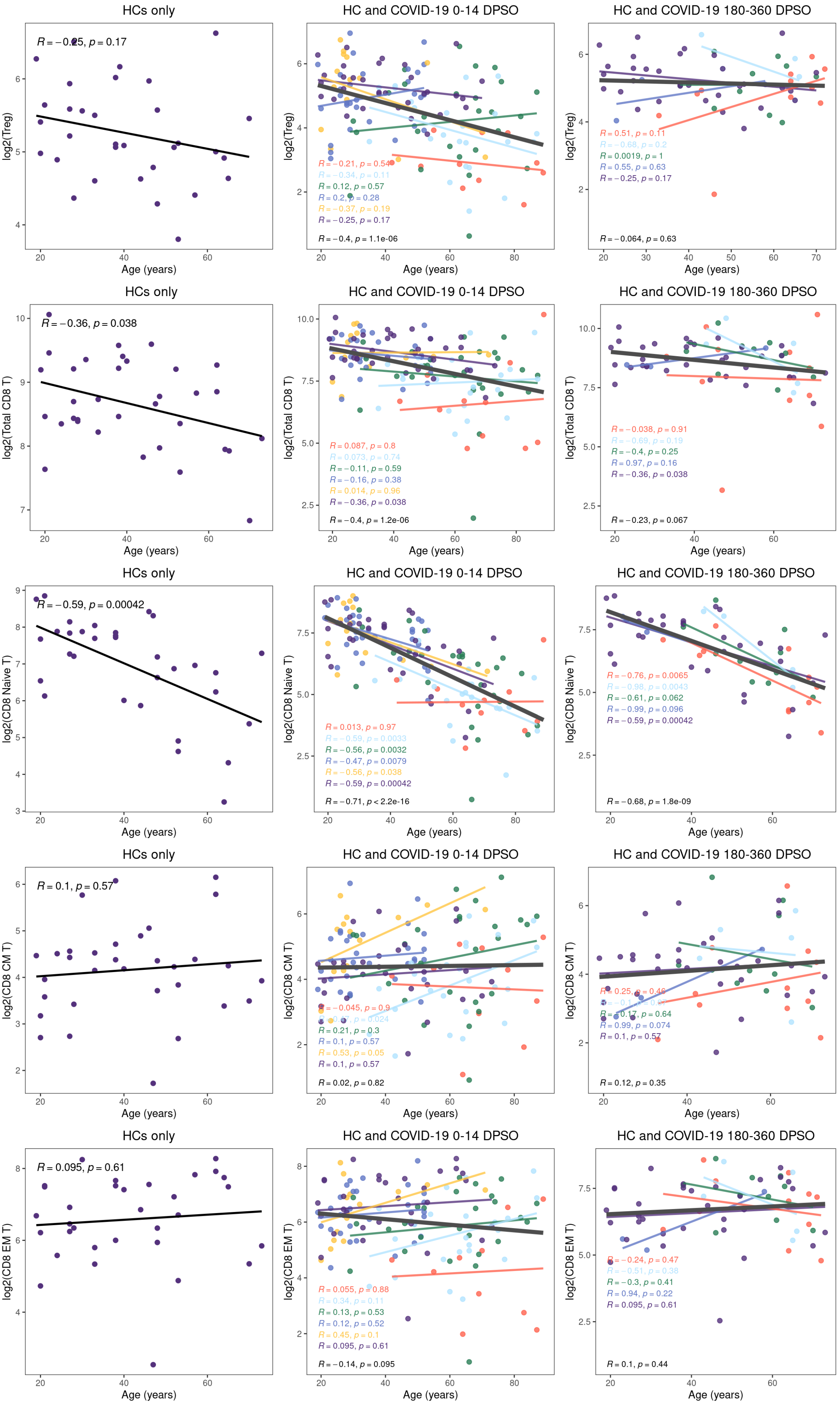
HCs only
R = −0.25, p = 0.17
log2(Treg)
Age (years)
HC and COVID-19 0-14 DPSO
R = −0.21, p = 0.54
R = −0.34, p = 0.11
R = 0.12, p = 0.57
R = 0.2, p = 0.28
R = −0.37, p = 0.19
R = −0.25, p = 0.17
R = −0.4, p = 1.1e-06
log2(Treg)
Age (years)
HC and COVID-19 180-360 DPSO
R = 0.51, p = 0.11
R = −0.68, p = 0.2
R = 0.0019, p = 1
R = 0.55, p = 0.63
R = −0.25, p = 0.17
R = −0.064, p = 0.63
log2(Treg)
Age (years)
HCs only
R = −0.36, p = 0.038
log2(Total CD8 T)
Age (years)
HC and COVID-19 0-14 DPSO
R = 0.087, p = 0.8
R = 0.073, p = 0.74
R = −0.11, p = 0.59
R = −0.16, p = 0.38
R = 0.014, p = 0.96
R = −0.36, p = 0.038
R = −0.4, p = 1.2e-06
log2(Total CD8 T)
Age (years)
HC and COVID-19 180-360 DPSO
R = −0.038, p = 0.91
R = −0.69, p = 0.19
R = −0.4, p = 0.25
R = 0.97, p = 0.16
R = −0.36, p = 0.038
R = −0.23, p = 0.067
log2(Total CD8 T)
Age (years)
HCs only
R = −0.59, p = 0.00042
log2(CD8 Naive T)
Age (years)
HC and COVID-19 0-14 DPSO
R = 0.013, p = 0.97
R = −0.59, p = 0.0033
R = −0.56, p = 0.0032
R = −0.47, p = 0.0079
R = −0.56, p = 0.038
R = −0.59, p = 0.00042
R = −0.71, p < 2.2e-16
log2(CD8 Naive T)
Age (years)
HC and COVID-19 180-360 DPSO
R = −0.76, p = 0.0065
R = −0.98, p = 0.0043
R = −0.61, p = 0.062
R = −0.99, p = 0.096
R = −0.59, p = 0.00042
R = −0.68, p = 1.8e-09
log2(CD8 Naive T)
Age (years)
HCs only
R = 0.1, p = 0.57
log2(CD8 CM T)
Age (years)
HC and COVID-19 0-14 DPSO
R = −0.045, p = 0.9
p = 0.024
R = 0.21, p = 0.3
R = 0.1, p = 0.57
R = 0.53, p = 0.05
R = 0.1, p = 0.57
R = 0.02, p = 0.82
log2(CD8 CM T)
Age (years)
HC and COVID-19 180-360 DPSO
R = 0.25, p = 0.46
R = 0.17, p = 0.64
R = 0.99, p = 0.074
R = 0.1, p = 0.57
R = 0.12, p = 0.35
log2(CD8 CM T)
Age (years)
HCs only
R = 0.095, p = 0.61
log2(CD8 EM T)
Age (years)
HC and COVID-19 0-14 DPSO
R = 0.055, p = 0.88
R = 0.34, p = 0.11
R = 0.13, p = 0.53
R = 0.12, p = 0.52
R = 0.45, p = 0.1
R = 0.095, p = 0.61
R = −0.14, p = 0.095
log2(CD8 EM T)
Age (years)
HC and COVID-19 180-360 DPSO
R = −0.24, p = 0.47
R = −0.51, p = 0.38
R = −0.3, p = 0.41
R = 0.94, p = 0.22
R = 0.095, p = 0.61
R = 0.1, p = 0.44
log2(CD8 EM T)
Age (years)

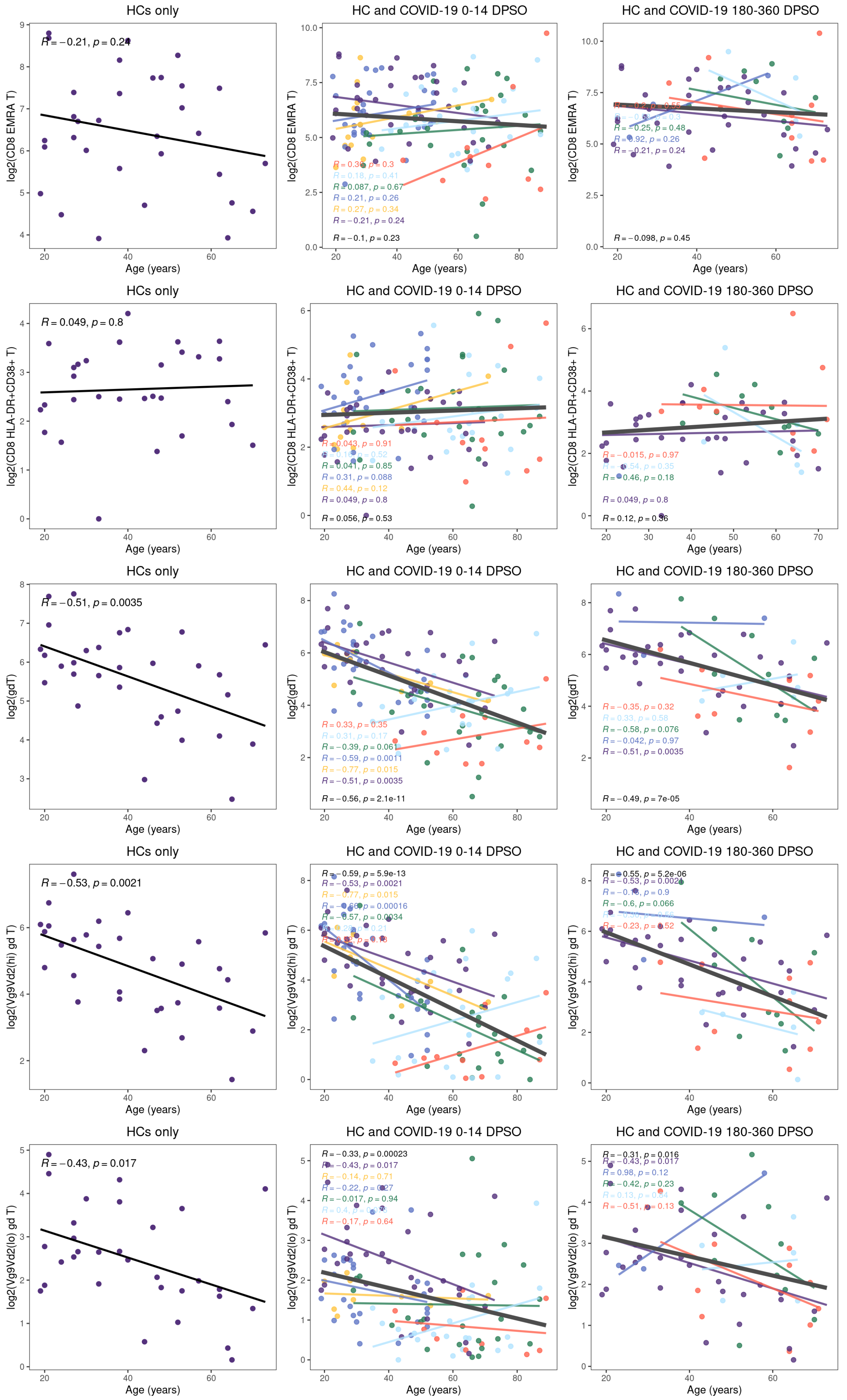

HCs only
HC and COVID-19 0-14 DPSO
HC and COVID-19 180-360 DPSO
log2(CD8 EMRA T)
log2(CD8 HLA-DR+CD38+ T)
log2(gdT)
log2(Vg9Vd2(hi) gd T)
log2(Vg9Vd2(lo) gd T)
Age (years)
R = −0.21, p = 0.24
R = −0.1, p = 0.23
R = −0.098, p = 0.45
R = 0.049, p = 0.8
R = 0.056, p = 0.53
R = 0.12, p = 0.36
R = −0.51, p = 0.0035
R = −0.56, p = 2.1e-11
R = −0.49, p = 7e-05
R = −0.53, p = 0.0021
R = −0.59, p = 5.9e-13
R = −0.55, p = 5.2e-06
R = −0.43, p = 0.017
R = −0.33, p = 0.00023
R = −0.31, p = 0.016

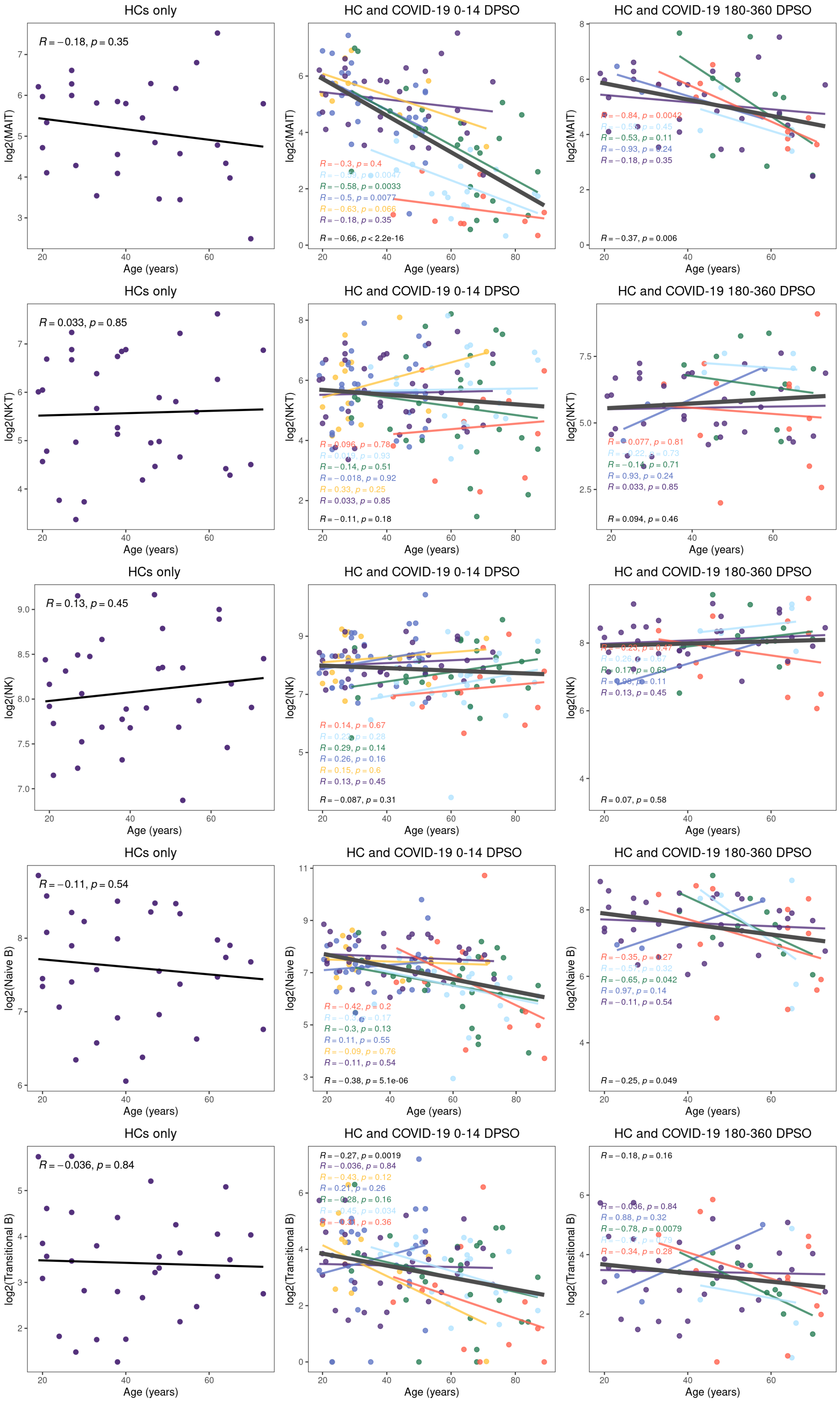

HCs only
HC and COVID-19 0-14 DPSO
HC and COVID-19 180-360 DPSO
R = −0.18, p = 0.35
R = −0.3, p = 0.4
R = −0.58, p = 0.0033
R = −0.5, p = 0.0077
R = −0.63, p = 0.066
R = −0.18, p = 0.35
R = −0.66, p < 2.2e-16
R = −0.84, p = 0.0042
R = −0.53, p = 0.11
R = −0.93, p = 0.24
R = −0.18, p = 0.35
R = −0.37, p = 0.006
log2(MAIT)
Age (years)
R = 0.033, p = 0.85
R = 0.096, p = 0.78
R = 0.019, p = 0.93
R = −0.14, p = 0.51
R = −0.018, p = 0.92
R = 0.33, p = 0.25
R = 0.033, p = 0.85
R = −0.11, p = 0.18
R = 0.077, p = 0.81
R = 0.93, p = 0.24
R = 0.033, p = 0.85
R = 0.094, p = 0.46
log2(NKT)
R = 0.13, p = 0.45
R = 0.14, p = 0.67
R = 0.29, p = 0.14
R = 0.26, p = 0.16
R = 0.15, p = 0.6
R = 0.13, p = 0.45
R = −0.087, p = 0.31
R = 0.13, p = 0.45
R = 0.07, p = 0.58
log2(NK)
R = −0.11, p = 0.54
R = −0.42, p = 0.2
R = −0.3, p = 0.13
R = 0.11, p = 0.55
R = −0.09, p = 0.76
R = −0.11, p = 0.54
R = −0.38, p = 5.1e-06
R = −0.35, p = 0.27
R = −0.65, p = 0.042
R = 0.97, p = 0.14
R = −0.11, p = 0.54
R = −0.25, p = 0.049
log2(Naive B)
R = −0.036, p = 0.84
R = −0.27, p = 0.0019
R = −0.036, p = 0.84
R = −0.43, p = 0.12
R = 0.21, p = 0.26
R = 0.45, p = 0.034
R = −0.18, p = 0.16
R = −0.036, p = 0.84
R = 0.88, p = 0.32
R = −0.78, p = 0.0079
R = −0.34, p = 0.28
log2(Transitional B)

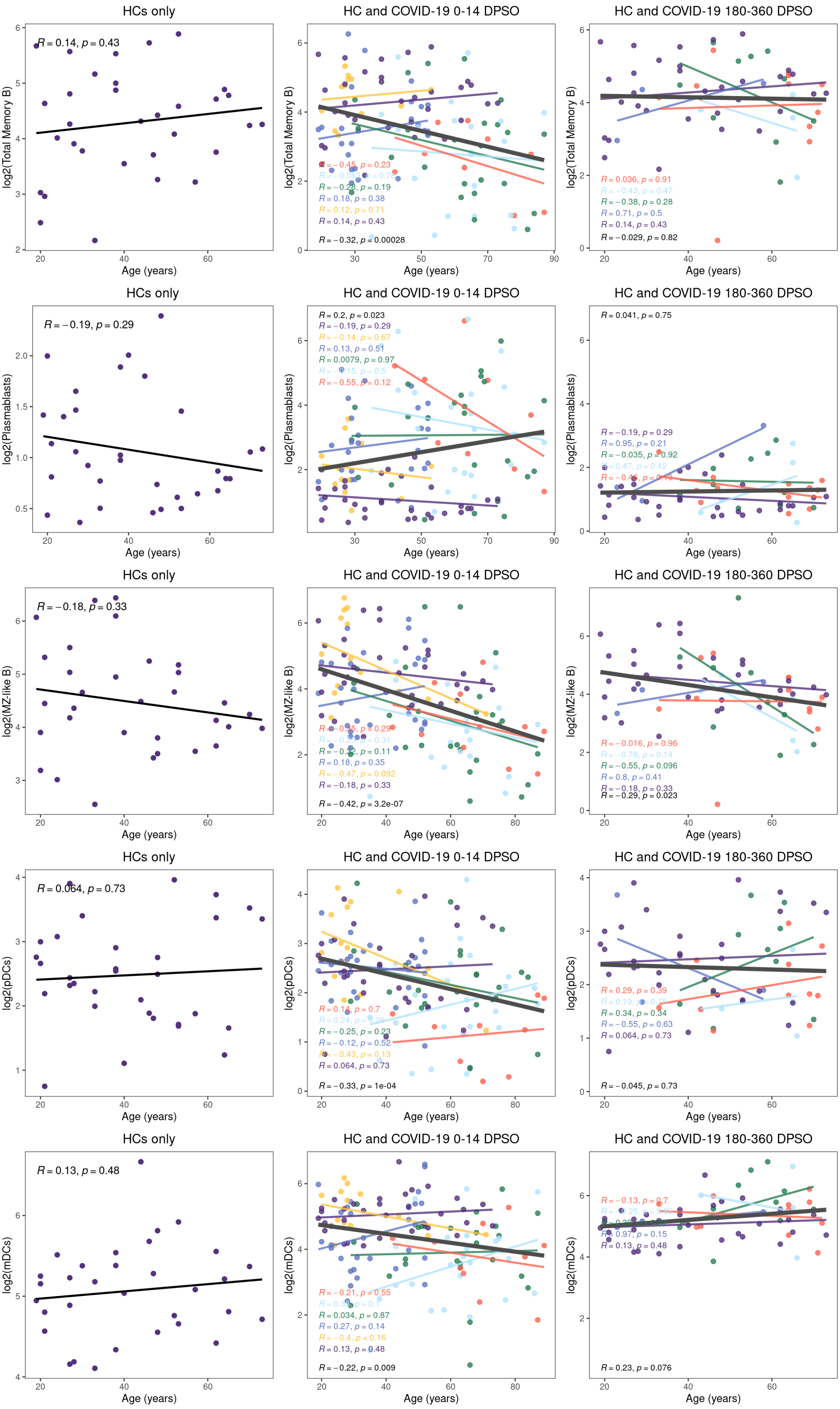

HCs only
HC and COVID-19 0-14 DPSO
HC and COVID-19 180-360 DPSO
log2(Total Memory B)
R = 0.14, p = 0.43
R = −0.45, p = 0.23
R = −0.28, p = 0.19
R = 0.18, p = 0.38
R = 0.12, p = 0.71
R = 0.14, p = 0.43
R = −0.32, p = 0.00028
R = 0.036, p = 0.91
R = −0.43, p = 0.47
R = −0.38, p = 0.28
R = 0.71, p = 0.5
R = 0.14, p = 0.43
R = −0.029, p = 0.82
log2(Plasmablasts)
R = −0.19, p = 0.29
R = 0.2, p = 0.023
R = −0.19, p = 0.29
R = −0.14, p = 0.67
R = 0.13, p = 0.51
R = 0.0079, p = 0.97
R = −0.55, p = 0.12
R = 0.041, p = 0.75
R = −0.19, p = 0.29
R = 0.95, p = 0.21
R = −0.035, p = 0.92
R = 0.47, p = 0.42
log2(MZ-like B)
R = −0.18, p = 0.33
R = −0.32, p = 0.11
R = 0.18, p = 0.35
R = −0.47, p = 0.092
R = −0.18, p = 0.33
R = −0.42, p = 3.2e-07
R = −0.016, p = 0.96
R = −0.76, p = 0.14
R = −0.55, p = 0.096
R = 0.8, p = 0.41
R = −0.18, p = 0.33
R = −0.29, p = 0.023
log2(pDCs)
R = 0.064, p = 0.73
R = 0.14, p = 0.7
R = −0.25, p = 0.23
R = −0.12, p = 0.52
R = −0.43, p = 0.13
R = 0.064, p = 0.73
R = −0.33, p = 1e-04
R = 0.29, p = 0.39
R = 0.34, p = 0.34
R = −0.55, p = 0.63
R = 0.064, p = 0.73
R = −0.045, p = 0.73
log2(mDCs)
R = 0.13, p = 0.48
R = −0.21, p = 0.55
R = 0.034, p = 0.87
R = 0.27, p = 0.14
R = −0.4, p = 0.16
R = 0.13, p = 0.48
R = −0.22, p = 0.009
R = −0.13, p = 0.7
R = 0.97, p = 0.15
R = 0.13, p = 0.48
R = 0.23, p = 0.076
Age (years)

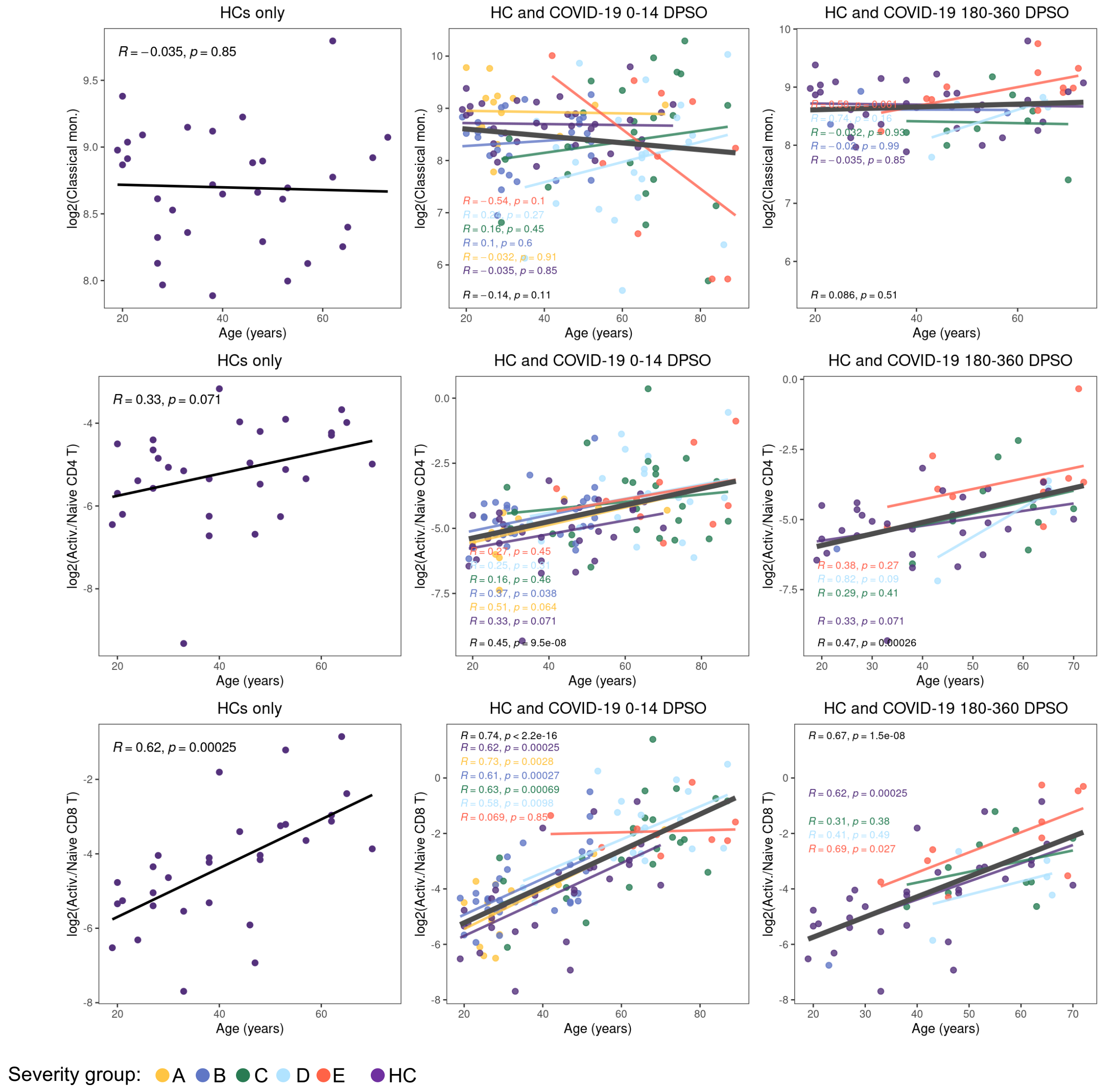

Figure SM2: Pearson’s correlation between age and measured immune cell counts in healthy controls (HC; left), within and across COVID-19 severity groups in early (day 0-14; middle) and late (day >=180; right) disease. DPSO = days post symptom onset.

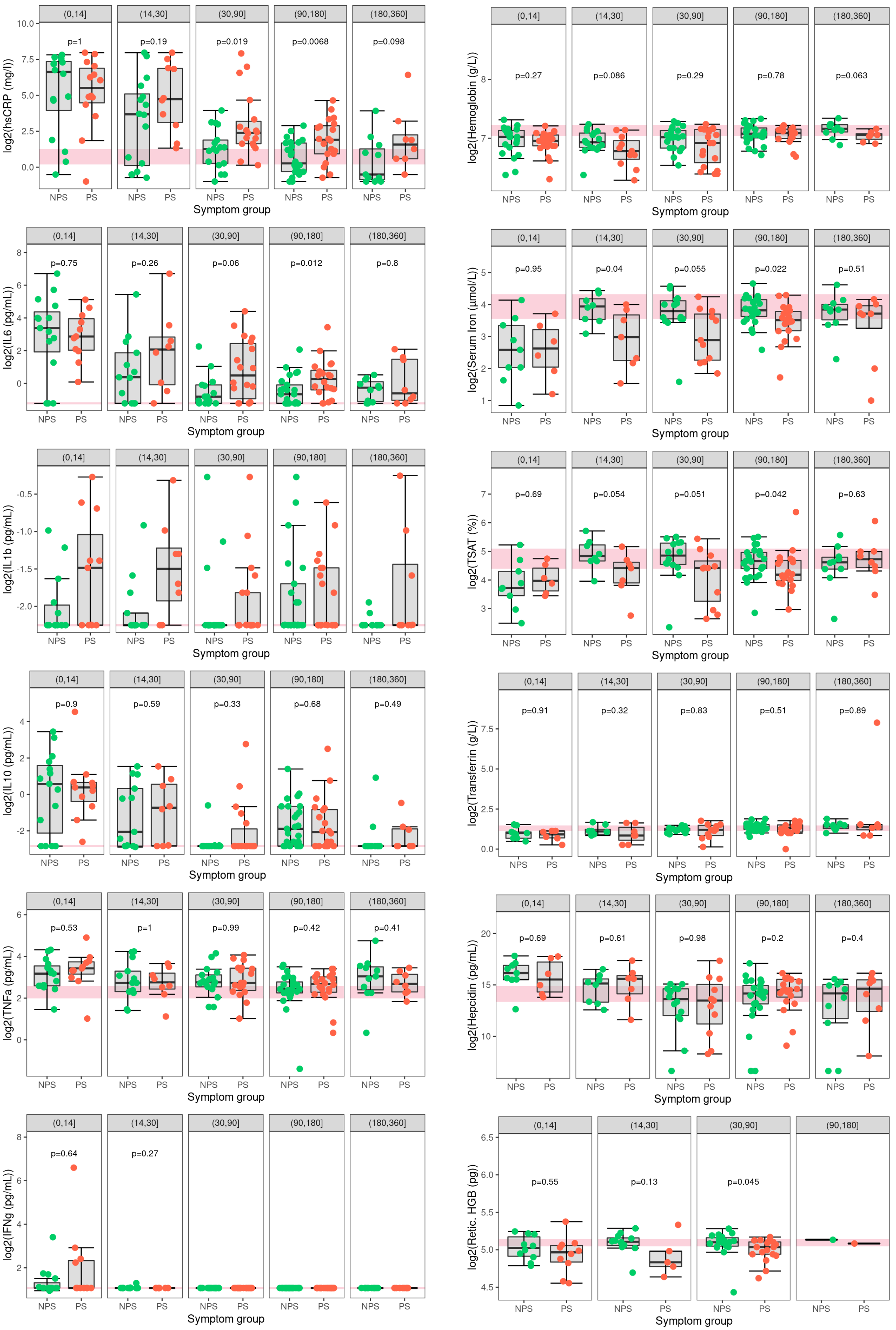
log2(hsCRP (mg/l))
(0,14] (14,30] (30,90] (90,180] (180,360]
p=1 p=0.19 p=0.019 p=0.0068 p=0.098
log2(Hemoglobin (g/L))
p=0.27 p=0.086 p=0.29 p=0.78 p=0.063
log2(IL6 (pg/mL))
p=0.75 p=0.26 p=0.06 p=0.012 p=0.8
log2(Serum Iron (µmol/L))
p=0.95 p=0.04 p=0.055 p=0.022 p=0.51
log2(IL1b (pg/mL))
log2(TSAT (%))
p=0.69 p=0.054 p=0.051 p=0.042 p=0.63
log2(IL10 (pg/mL))
p=0.9 p=0.59 p=0.33 p=0.68 p=0.49
log2(Transferrin (g/L))
p=0.91 p=0.32 p=0.83 p=0.51 p=0.89
log2(TNFa (pg/mL))
p=0.53 p=1 p=0.99 p=0.42 p=0.41
log2(Hepcidin (pg/mL))
p=0.69 p=0.61 p=0.98 p=0.2 p=0.4
log2(IFNg (pg/mL))
p=0.64 p=0.27
log2(Retic. HGB (pg))
(0,14] (14,30] (30,90] (90,180]
p=0.55 p=0.13 p=0.045
NPS PS
Symptom group

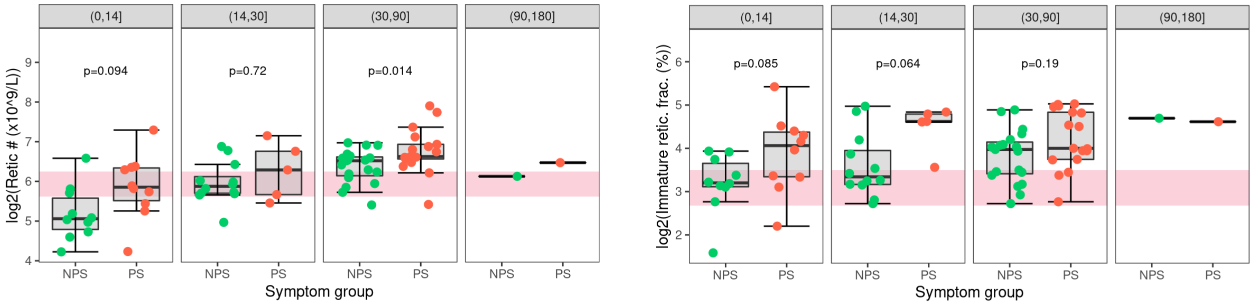

Figure SM3: Comparison of clinical parameters over time in age matched COVID-19 patients >50 years reporting persisting symptoms (PS) or no persisting symptoms (NPS) at 3-5 months post symptom onset. P-values are calculated by Wilcoxon test.

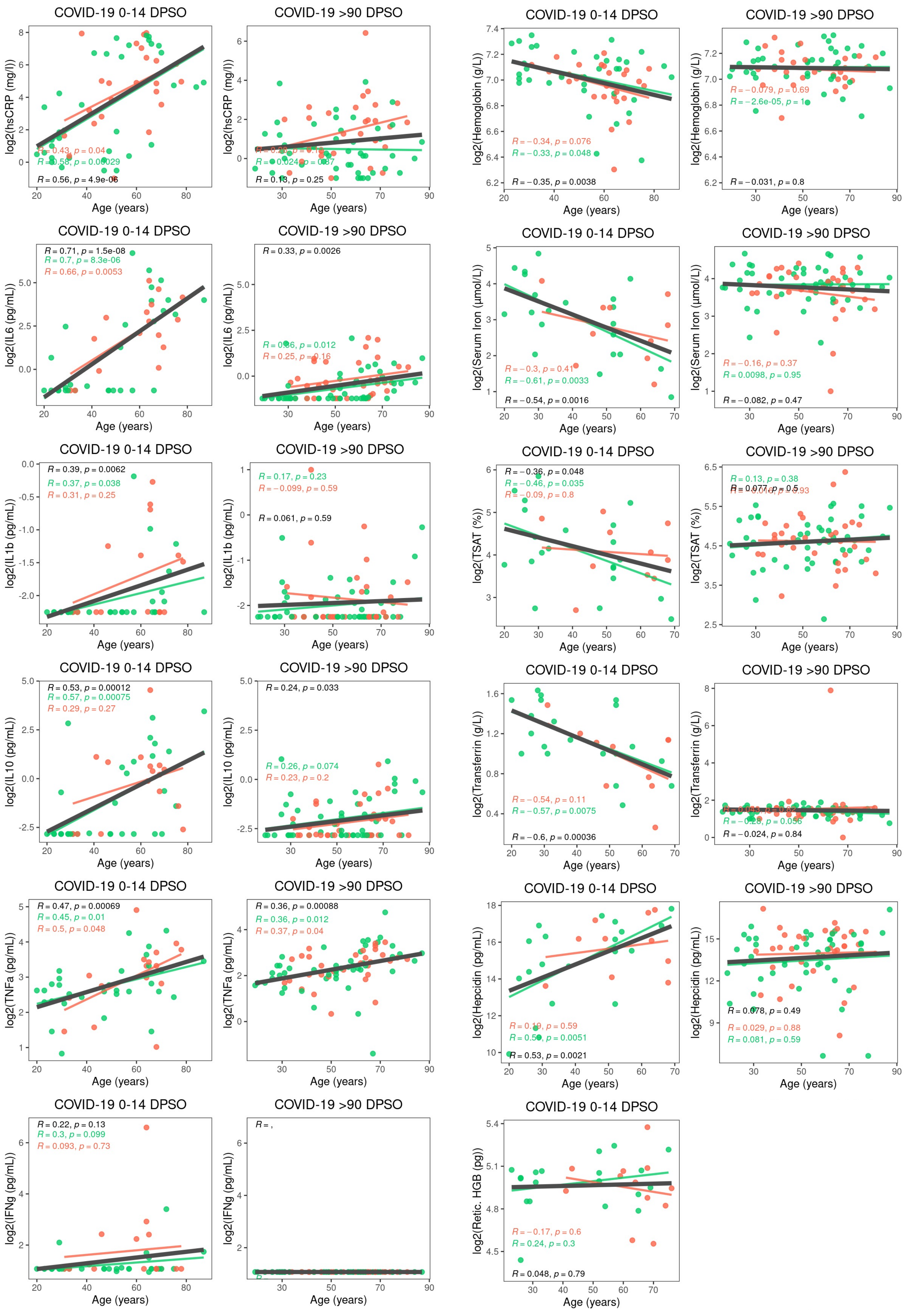
COVID-19 0-14 DPSO
log2(hsCRP (mg/l))
R = 0.43, p = 0.04
R = 0.58, p = 0.00029
R = 0.56, p = 4.9e-06
Age (years)
COVID-19 >90 DPSO
log2(hsCRP (mg/l))
R = 0.13, p = 0.25
Age (years)
COVID-19 0-14 DPSO
log2(Hemoglobin (g/L))
R = −0.34, p = 0.076
R = −0.33, p = 0.048
R = −0.35, p = 0.0038
Age (years)
COVID-19 >90 DPSO
log2(Hemoglobin (g/L))
R = −0.079, p = 0.69
R = −2.6e-05, p = 1
R = −0.031, p = 0.8
Age (years)
COVID-19 0-14 DPSO
log2(IL6 (pg/mL))
R = 0.71, p = 1.5e-08
R = 0.7, p = 8.3e-06
R = 0.66, p = 0.0053
Age (years)
COVID-19 >90 DPSO
log2(IL6 (pg/mL))
R = 0.33, p = 0.0026
R = 0.36, p = 0.012
R = 0.25, p = 0.16
Age (years)
COVID-19 0-14 DPSO
log2(Serum Iron (µmol/L))
R = −0.3, p = 0.41
R = −0.61, p = 0.0033
R = −0.54, p = 0.0016
Age (years)
COVID-19 >90 DPSO
log2(Serum Iron (µmol/L))
R = −0.16, p = 0.37
R = 0.0098, p = 0.95
R = −0.082, p = 0.47
Age (years)
COVID-19 0-14 DPSO
log2(IL1b (pg/mL))
R = 0.39, p = 0.0062
R = 0.37, p = 0.038
R = 0.31, p = 0.25
Age (years)
COVID-19 >90 DPSO
log2(IL1b (pg/mL))
R = 0.17, p = 0.23
R = −0.099, p = 0.59
R = 0.061, p = 0.59
Age (years)
COVID-19 0-14 DPSO
log2(TSAT (%))
R = −0.36, p = 0.048
R = −0.46, p = 0.035
R = −0.09, p = 0.8
Age (years)
COVID-19 >90 DPSO
log2(TSAT (%))
R = 0.13, p = 0.38
R = 0.077, p = 0.5
Age (years)
COVID-19 0-14 DPSO
log2(IL10 (pg/mL))
R = 0.53, p = 0.00012
R = 0.57, p = 0.00075
R = 0.29, p = 0.27
Age (years)
COVID-19 >90 DPSO
log2(IL10 (pg/mL))
R = 0.24, p = 0.033
R = 0.26, p = 0.074
R = 0.23, p = 0.2
Age (years)
COVID-19 0-14 DPSO
log2(Transferin (g/L))
R = −0.54, p = 0.11
R = −0.57, p = 0.0075
R = −0.6, p = 0.00036
Age (years)
COVID-19 >90 DPSO
log2(Transferin (g/L))
R = −0.024, p = 0.84
Age (years)
COVID-19 0-14 DPSO
log2(TNFa (pg/mL))
R = 0.47, p = 0.00069
R = 0.45, p = 0.01
R = 0.5, p = 0.048
Age (years)
COVID-19 >90 DPSO
log2(TNFa (pg/mL))
R = 0.36, p = 0.00088
R = 0.36, p = 0.012
R = 0.37, p = 0.04
Age (years)
COVID-19 0-14 DPSO
log2(Hepcidin (pg/mL))
R = 0.53, p = 0.0021
Age (years)
COVID-19 >90 DPSO
log2(Hepcidin (pg/mL))
R = 0.078, p = 0.49
R = 0.029, p = 0.88
R = 0.081, p = 0.59
Age (years)
COVID-19 0-14 DPSO
log2(IFNg (pg/mL))
R = 0.22, p = 0.13
R = 0.3, p = 0.099
R = 0.093, p = 0.73
Age (years)
COVID-19 >90 DPSO
log2(IFNg (pg/mL))
R = ,
Age (years)
COVID-19 0-14 DPSO
log2(Retic. HGB (pg))
R = −0.17, p = 0.6
R = 0.24, p = 0.3
R = 0.048, p = 0.79
Age (years)

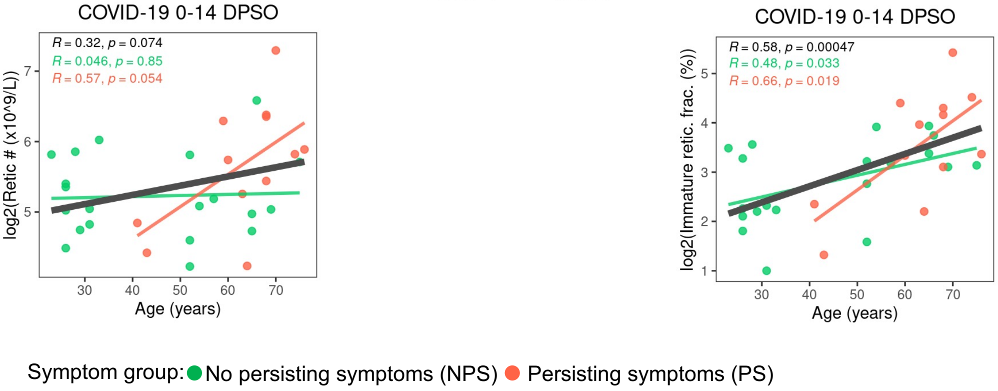

Figure SM4: Pearson's correlation between age and measured clinical parameters in COVID-19 patients during early (day 0-14; left) and late (day > 90; right) disease. Points are colored by PASC symptom group as based on Q1 questionnaires (collected at 3-5 months). Reticulocyte parameters were not measured at late timepoints, so data is lacking. DPSO = days post symptom onset.

Supplementary Note

COVID FU Screening Tool V9

Dates of admission:	ICU: YES / NO	IF YES, FOR ICU FU
Pneumonia *(check discharge summary or Chest XRay reports):* **YES/NO**		
Known COVID complications identified during admission:		

Do you feel you have made a full physical and mental recovery from COVID 19? Yes/No
If not, what % of your usual function are currently at?

The following questions refer to **NEW** symptoms since COVID, or a significant change from state.

Question	0	1	2	3	4	5	**Action**
**Q1 Dyspnoea**<br>Modified MRC Dyspnoea scale	Extremely short of breath<br>Unable to leave the house, or breathless on dressing	Severely short of breath	Markedly short of breath<br>Walks slower than most people of the same age because of dyspnoea OR has to stop for breath when walking at own pace	Moderately short of breath<br>E.g. when hurrying or walking up a slight hill	Mildly short of breath<br>Slightly more breathless than usual on physical exertion	No shortness of breath<br>Or same as previous baseline	**If 2 or less, clinic with Respiratory Medicine**
**Q2 Cough**	Cough interfering with daily life and sleep all the time	Cough interfering with daily life and sleep most of the time	Cough interfering with daily life and sleep a good bit of the time	Cough interfering with daily life and sleep some of the time	Cough interfering with daily life and sleep occasionally	No cough<br>Or same as previous baseline	**If 2 or less, clinic with Respiratory Medicine**

**Q3 Chest pain** on exertion, palpitations or swollen ankles?	Yes	No	**If YES, clinic with Cardiology**
**Q4 A fever** that has persistent for 2 months or more?	Yes	No	**If YES, clinic with Infectious diseases**
**Q5 Any new swelling** in one leg or sudden onset worsening shortness of breath with chest pain?	Yes	No	**If YES, discuss with Haematology**
**Q6 Any new skin rashes** or sores since COVID which have not gone away?	Yes	No	**If YES, discuss with Dermatology**
**Q7** a) Have you or your family noticed any changes to your voice such as difficulty being heard, altered quality of the voice, your voice tiring by the end of the day or an inability to alter the pitch of your voice?	Yes	No	**If YES to any, refer to ENT Joint COVID Clinic**
b) Are you having difficulties eating, drinking or swallowing such as coughing, choking or avoiding any food or drinks?	Yes	No	

Not for use

Question	0	1	2	3	4	5	Action
c) Do you have any CONSTANT noisy breathing or whistling from your throat?					Yes / No		
d) Has your sense of smell deteriorated or disappeared altogether?					Yes / No		
**Q8 A**re you finding it difficult to gain or maintain your weight, or are you find your appetite is still poor?					Yes / No		**If YES, refer to Dietician**
**Q9 New neurology** symptoms in one or more limbs	Extreme neurological deficit<br>Complete paralysis of one or more limbs	Severe neurological deficit<br>Weakness or numbness in one or more limbs causing significant limitation in day to day life e.g. in mobilizing, ADLs etc	Significant neurological deficit<br>Weakness and numbness in one or more limbs noticeable in day to day life<br>New difficulties with balance or vision	Moderate neurological deficit<br>Mild weakness or numbness in a single limb, with or without pain and tingling	Mild neurological deficit<br>E.g. peripheral tingling in fingers and toes, no weakness or numbness	No new neurological symptoms	**If 4 or less, clinic with Neurology**
**Q10 Any new pain in one or more part of the body?**	Extreme pain<br>Interfering with sleep and almost all activities. Pain medications have little or no effect	Severe pain<br>Not controlled by medication or interventions, interferes with daily activities every day	Significant pain<br>Medications/interventions partially effective, interferes with activities most days	Moderate pain<br>Helped by medication, occasionally interferes with activities	Mild pain<br>Mild pain, well controlled	No pain	**If 2 or less, consider Rehab Med input**
**Q11 Physical problems** with generalized muscle weakness, balance or range of movement of joints	Extreme<br>E.g. bed bound	Severe problems<br>e.g. generalized weakness, able to sit in a chair but not able to mobilise independently	Severe problems- able to mobilise short distances with aids such as stick or frame	Moderate problems- able to mobilise around the house but not manage stairs or walk outside	Mild problems-<br>Able to walk around and manage day to day activities but not back to usual baseline	Back to normal	**Trigger for Physio assessment:**<br>**Any change from baseline score and patient feels overall lack of progress**

Not for use

COVID FU Screening Tool V9

Question	0	1	2	3	4	5	Action
**Q12 Fatigue**	Extreme fatigue<br>Spends most of the day in bed or chair	Severe fatigue<br>Fatigue impacts severely on daily activities, requires several rest periods during the day	Marked fatigue<br>Fatigue impacts significantly on daily activities, needs a rest during the day	Moderate fatigue<br>Fatigue requires modification of some usual activities e.g. working part time, limited exercise but able to carry out basic daily activities	Mild<br>Able to carry out normal activities (including work) but tired at the end of the day	No fatigue<br>Normal stamina	**If 3 or less, consider Rehab Med input**
**Q13 Cognition**<br>Memory, concentration and thinking skills	Unconscious	Awake but drowsy	Severe cognitive impairment<br>Markedly confused	Moderate cognitive impairment<br>Not fully orientated<br>Severe memory problems	Fully orientated but some higher-level problems e.g. memory, attention and/or higher-level functioning	Normal cognition	**If 3 or less consider Rehab Med input**
**Q14 Care needs** for basic daily activities	Totally dependent<br>2 or more carers throughout 24hr period	Severe dependence<br>1 carer throughout 24hr period<br>E.g. needing help to get from bed to chair	Marked dependence<br>1 carer required, unable to live unsupported<br>E.g. needing help with toileting	Moderate dependence<br>1-2 carers required on a visiting basis	Mild dependence, incidental help or support for extended activities, once/day or less	Fully independent	**If 3 or less, consider Rehab Med input**
**Q15 Mental Health**	Pre-existing mental illness with ongoing secondary mental health input	Severe mental health problems (e.g. depression, anxiety) preventing any engagement in daily activities	Marked anxiety/mood/stress problems that impact significantly on daily function and ability to engage in rehab	Moderate anxiety/mood issues with some impact on function, requiring intervention	Mild anxiety or mood issues which do not impact on daily function but require further support	No mental health issues	**Identify patients with a score less than 5, exact referral process to be confirmed**
**Q16 Family/friends**		Family and/or friends distressed by events of patient's admission			Family/friends distressed and needing an opportunity to discuss what happened in clinic setting	No concerns from family	**If 1, should be invited to attend a MDT FU Clinic**

Not for use

Q17 Do you have any other concerns about your recovery from COVID 19? **Yes / No**

Q18 Are you coping with your current situation? **Yes / No –** If NO, prioritise appointment

Q19 Do you already have clinic appointments planned for this or other chronic medical problems? **Yes / No** If YES, no need for re-referral to same specialty unless new or worsening symptom

Q20 Have you had a Chest X-Ray or Chest CT scan since you left hospital, or do you have an appointment for one? **Yes/No**

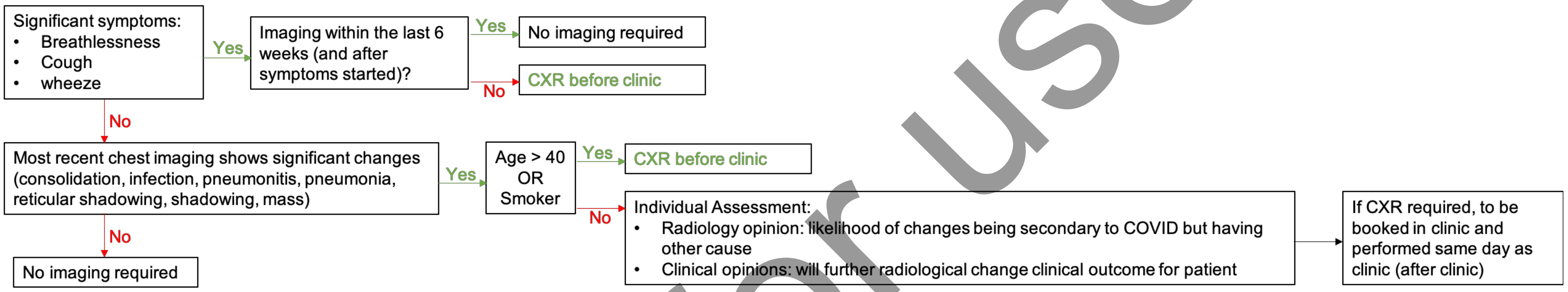

Q21 Would you be happy to attend the follow up clinic, either in person or virtually, if we send you an appointment? **Yes / No**

Q22 Would you be happy to be approached about helping with research to improve our understanding of COVID 19? - **Yes / No**

Not for use
