# Supplementary material for: Iron dysregulation and inflammatory stress erythropoiesis associates with long-term outcome of COVID-19
Source: Nat Immunol. 2024 Mar 1;25(3):471–82. doi: 10.1038/s41590-024-01754-8 (PMC10907301; doi:10.1038/s41590-024-01754-8)
Supplement: Supplementary file 2 — Reporting Summary [file 41590_2024_1754_MOESM2_ESM.pdf]

nature portfolio

Corresponding author(s): Aimee L. Hanson

Last updated by author(s): 15/10/23

Reporting Summary

Nature Portfolio wishes to improve the reproducibility of the work that we publish. This form provides structure for consistency and transparency in reporting. For further information on Nature Portfolio policies, see our Editorial Policies and the Editorial Policy Checklist.

Statistics

For all statistical analyses, confirm that the following items are present in the figure legend, table legend, main text, or Methods section.

n/a	Confirmed	
☐	☒	The exact sample size (*n*) for each experimental group/condition, given as a discrete number and unit of measurement
☐	☒	A statement on whether measurements were taken from distinct samples or whether the same sample was measured repeatedly
☐	☒	The statistical test(s) used AND whether they are one- or two-sided<br>*Only common tests should be described solely by name; describe more complex techniques in the Methods section.*
☐	☒	A description of all covariates tested
☐	☒	A description of any assumptions or corrections, such as tests of normality and adjustment for multiple comparisons
☐	☒	A full description of the statistical parameters including central tendency (e.g. means) or other basic estimates (e.g. regression coefficient) AND variation (e.g. standard deviation) or associated estimates of uncertainty (e.g. confidence intervals)
☐	☒	For null hypothesis testing, the test statistic (e.g. *F*, *t*, *r*) with confidence intervals, effect sizes, degrees of freedom and *P* value noted<br>*Give P values as exact values whenever suitable.*
☒	☐	For Bayesian analysis, information on the choice of priors and Markov chain Monte Carlo settings
☒	☐	For hierarchical and complex designs, identification of the appropriate level for tests and full reporting of outcomes
☐	☒	Estimates of effect sizes (e.g. Cohen's *d*, Pearson's *r*), indicating how they were calculated

Our web collection on statistics for biologists contains articles on many of the points above.

Software and code

Policy information about availability of computer code

Data collection	Flow cytometry data was gated using FlowJo v10.2. RNA sequencing data preprocessing was performed using a standard pipeline on a compute cluster. Briefly, sequencing read quality was assessed using FastQC v0.11.8 (Babraham Bioinformatics, UK), with trimming of SMARTer adaptors and poor-quality terminal bases (Phred score threshold <24) with Trim Galore v.0.6.4 (Babraham Bioinformatics, UK). Ribosomal rRNA contamination was removed using BBSplit (BBMap v.38.67) and clean reads aligned to the human reference genome GRCh38 using HISAT2 v2.1.0. Alignment .bam files were merged and read count matrix generated using the function featureCounts from the R package Rsubread (v.2.0.1). Count data was stored in a DGEList object with accompanying gene annotations and patient metadata for downstream handling (EdgeR v.3.28.1).
Data analysis	All statistical analysis was performed using R v3.6.0 with base and publicly available analysis packages: limma v3.42.2, edgeR v3.28.1, seurat v4.2.1 (in R v4.2.2 for preliminary data processing), dsb v1.0.2, mixOmics v6.10.9. Conversion between gene identifiers for RNASeq data analysis was facilitated by AnnotationDbi v1.48.0.

For manuscripts utilizing custom algorithms or software that are central to the research but not yet described in published literature, software must be made available to editors and reviewers. We strongly encourage code deposition in a community repository (e.g. GitHub). See the Nature Portfolio guidelines for submitting code & software for further information.

Data

Policy information about availability of data

All manuscripts must include a data availability statement. This statement should provide the following information, where applicable:

- Accession codes, unique identifiers, or web links for publicly available datasets
- A description of any restrictions on data availability
- For clinical datasets or third party data, please ensure that the statement adheres to our policy

All datasets used in the generation of presented figures, including cell counts, serum measures, Sysmex hematology data, PAXGene whole-blood RNASeq gene expression counts, patient metadata and PASC group assignments can be downloaded from the Zenodo repository DOI: 10.5281/zenodo.10161238. Whole blood RNA-seq data are available through the European Genome-phenome Archive (EGA, ID: EGAS00001005332). CITE-seq processed data are available to download from Array Express using accession number E-MTAB-10026. Published CITE-seq data from Schulte-Schrepping et al., used for replication analysis, are available through the EGA (ID: EGAS00001004571). HALLMARK and WP_FERROPTOSIS gene sets are accessible through the MSigDB database: https://www.gsea-msigdb.org/gsea/msigdb/. The IRE gene set ("IRE_HQ") is available as supplement in the article by Hin et al., DOI: 10.3233/jad-210200, the iron-starvation gene set was taken from Table 3 ("genes upregulated in iron-free medium") in the associated publication by Chicault et al., DOI: 10.1152/physiolgenomics.00297.2005. Gene sets are also available in Supplementary Tables 2-4.

Human research participants

Policy information about studies involving human research participants and Sex and Gender in Research.

Reporting on sex and gender	Self reported biological sex information was collected on all participants. Males and females were analysed together throughout with correction for sex as a covariate in RNASeq analyses.
Population characteristics	Human participants recruited for this study in Cambridge, UK, were of both male and female sex and spanned an age range from ~20-90 years. Ethnicity was not a grounds for exclusion, however the majority of the cohort identified as "white", reflective of the predominance of this group in the UK population. COVID patients were confirmed SARS-CoV-2 positive via PCR testing at recruitment, healthy controls were confirmed SARS-CoV-2 negative or recruited prior to the outbreak of the SARS-CoV-2 virus. Hospitalisation or maximal oxygenation requirements were taken from available clinical records or reported by participants at the time of recruitment, and used to stratify patients based on disease severity as specified in the Methods.
Recruitment	Study participants were recruited from patients attending Addenbrooke's and Royal Papworth Hospitals in Cambridge, or Peterborough Foundation Trust, with a PCR test confirmed diagnosis of COVID-19, together with health care works attending the staff COVID-19 screening programme at Addenbrooke's Hospital. Controls were recruited from among screened staff members testing negative for SARS-CoV-2 infection, to supplement sampling from previously collected control recruited as part of the "Genetic variation AND Altered Leucocyte Function in health and disease" (GANDALF) study (below). Recruitment of inpatients at Addenbrooke's Hospital and Health Care Workers was undertaken by the NIHR Cambridge Clinical Research Facility outreach team and the NIHR BioResource research nurse team. Due to the bias towards older males in hospitalised COVID-19 groups, our moderate-severe disease severity groups were skewed towards this demographic. Milder disease patients were more predominantly younger females, the predominant demographic of health care workers sampled. We attempted to recruit controls matched to the full age range and gender split of the patient cohort where possible. Age and sex were corrected for in statistical analyses were they were found to be significant confounding factors. No statistical methods were used to pre-determine sample sizes, and recruitment was based on access to and availability of participants during national lockdown.
Ethics oversight	Ethics approval was obtained from the East of England – Cambridge Central Research Ethics Committee (''NIHR BioResource'' REC ref 17/EE/0025, and ''Genetic variation AND Altered Leucocyte Function in health and disease - GANDALF'' REC ref 08/H0308/176). All participants provided informed consent.

Note that full information on the approval of the study protocol must also be provided in the manuscript.

Field-specific reporting

Please select the one below that is the best fit for your research. If you are not sure, read the appropriate sections before making your selection.

☒ Life sciences ☐ Behavioural & social sciences ☐ Ecological, evolutionary & environmental sciences

For a reference copy of the document with all sections, see nature.com/documents/nr-reporting-summary-flat.pdf

Life sciences study design

All studies must disclose on these points even when the disclosure is negative.

Sample size	Samples were collected in a time-sensitive manner during the early days of the first COVID-19 outbreak, with new patient recruitment halted when numbers were deemed sufficient for statistical analysis (a n>= 18 for each classified severity group). Repeated sampling was performed

for as long as participants where in hospital, or upon follow-up appointment. To maximise statistical power, samples were analysed in time windows post COVID-19 symptom onset, spanning two weeks to three months in length, retaining the earliest sampling timepoint per participant within these windows. Where available samples fell below n=3 for a given severity group within a given time window, data was not analysed to avoid spurious interpretation.

Data exclusions	Following clinician review, 6 originally recruited cases were considered not classifiable, due to complex concomitant pathologies that coexisted with COVID-19 and dominated the clinical picture, confounding the interpretation of clinical outcome. These cases were not included in any analyses. Samples collected from participants at any timepoint following vaccination with a SARS-CoV-2 specific mRNA vaccine, or from individuals where vaccine status could not be ascertained following roll-out of UK COVID-19 vaccination progammes in Cambridge in December 2020, were excluded. This was following preliminary analysis identifying an influence of vaccination on immune cell and cytokine parameters. Nineteen samples with fewer than 2,000,000 assigned reads, and one sample with an abnormal read distribution were excluded from RNASeq analysis following standard quality filtering.
Replication	The extensive longitudinal nature of this study made it difficult to find comparable COVID-19 cohorts with which to replicate analysis of late stage disease parameters (beyond one month post symptom onset). We did however seek to replicate the single-cell investigation of altered iron homeostatsis in blood cell populations in early disease. Analysis of publicly available data from Schulte-Schrepping et al. (Cell 2020) confirmed the predominant expression of iron homeostasis genes in monocyte subpopulations, and the decreased number of cells in these populations in the periphery of COVID-19 patients (Supp Fig 13C).
Randomization	Samples were randomised by the primary outcome variable (severity group) across all conducted assays. To test for and correct potential drift in instrumentation readings over time in a study spanning one year in duration, technical replicates or standards were included in all biological assays. Compensation controls were used to test for drift in marker fluorescence over time in flow-cytometry data, and gating rigorously checked across early and late timepoint samples by three independent analysts. All hospital run assays included internal controls. Healthy controls and asymptomatic (group A) patients (who showed no evidence of differential expression relative to healthy controls at any timepoint) were used to test for batch effects in temporally processed and sequenced PAXgene extracted RNA. Batch was included as a covariate in the statistical analysis of RNASeq data.
Blinding	No blinding was performed as careful clinical curation of severity groups was required for the study design.

Reporting for specific materials, systems and methods

We require information from authors about some types of materials, experimental systems and methods used in many studies. Here, indicate whether each material, system or method listed is relevant to your study. If you are not sure if a list item applies to your research, read the appropriate section before selecting a response.

Materials & experimental systems

n/a	Involved in the study	
☐	☒	Antibodies
☒	☐	Eukaryotic cell lines
☒	☐	Palaeontology and archaeology
☒	☐	Animals and other organisms
☒	☐	Clinical data
☒	☐	Dual use research of concern

Methods

n/a	Involved in the study	
☒	☐	ChIP-seq
☐	☒	Flow cytometry
☒	☐	MRI-based neuroimaging

Antibodies

Antibodies used	All antibodies used in this study, supplier names and product codes are provided in the CellPress Star Methods section of the publication that first describes this cohort (Bergamaschi, L et al. 2021, Immunity).
Validation	The immunophenotyping flow cytometry panels used in this study were adapted from rigorously validated panels previously developed by members of the NIHR Cambridge BRC Cell Phenotyping Hub for immune phenotyping in complex human immune-mediated diseases.

Flow Cytometry

Plots

Confirm that:

☐ The axis labels state the marker and fluorochrome used (e.g. CD4-FITC).

☐ The axis scales are clearly visible. Include numbers along axes only for bottom left plot of group (a 'group' is an analysis of identical markers).

☐ All plots are contour plots with outliers or pseudocolor plots.

☒ A numerical value for number of cells or percentage (with statistics) is provided.

Methodology

Sample preparation	Each participant provided 27 mL of peripheral venous blood collected into 9 mL sodium citrate tube. Peripheral blood mononuclear cells (PBMCs) were isolated using Leucosep tubes (Greiner Bio-One) with Histopaque 1077 (Sigma) by centrifugation at 800x g for 15 min at room temperature. PBMCs at the interface were collected, rinsed twice with autoMACS running buffer (Miltenyi Biotech) and cryopreserved in FBS with 10% DMSO. All samples were processed within 4 h of collection.
Instrument	5-laser BD Symphony X-50 flow cytometer
Software	FlowJo v10.2
Cell population abundance	For direct enumeration of T, B and NK cells, an aliquot of whole blood (50 ml) was added to BD TruCount tubes with 20ml- BD Mul- titest 6-color TBNK reagent (BD Biosciences) and processed as per the manufacturer's instructions.<br>Samples were gated in FlowJo v10.2 according to the schema set out in Bergamaschi, L et al. 2021 (Immunity) Data S4. The number of cells falling within each gate was recorded. For analysis, these were expressed as an absolute concentration of cells per ml, calculated using the proportions of daughter populations present within the parent population determined using the BD TruCountsystem.
Gating strategy	A detailed gating schema is set out in Bergamaschi, L et al. 2021 (Immunity) Data S4

☒ Tick this box to confirm that a figure exemplifying the gating strategy is provided in the Supplementary Information.
